# MEK1 drives oncogenic signaling and interacts with PARP1 for genomic and metabolic homeostasis in malignant pleural mesothelioma

**DOI:** 10.1038/s41420-023-01307-2

**Published:** 2023-02-10

**Authors:** Haitang Yang, Yanyun Gao, Duo Xu, Ke Xu, Shun-Qing Liang, Zhang Yang, Amina Scherz, Sean R. R. Hall, Stefan Forster, Sabina Berezowska, Feng Yao, Adrian F. Ochsenbein, Thomas M. Marti, Gregor J. Kocher, Ralph A. Schmid, Patrick Dorn, Ren-Wang Peng

**Affiliations:** 1 Department of Thoracic Surgery, Shanghai Chest Hospital, Shanghai Jiao Tong University, 200030 Shanghai, China; 2 Department of General Thoracic Surgery, Department of BioMedical Research (DBMR), Inselspital, Bern University Hospital, University of Bern, 3008 Bern, Switzerland; 3 Department of Medical Oncology, Inselspital, Bern University Hospital, University of Bern, 3008 Bern, Switzerland; 4 Department of Laboratory Medicine and Pathology, Institute of Pathology, Lausanne University Hospital and University of Lausanne, Lausanne, Switzerland; 5 Present Address: University of Massachusetts Medical School, Worcester, MA 01605 USA

**Keywords:** Targeted therapies, Drug development

## Abstract

Malignant pleural mesothelioma (MPM) is a lethal malignancy etiologically caused by asbestos exposure, for which there are few effective treatment options. Although asbestos carcinogenesis is associated with reactive oxygen species (ROS), the bona fide oncogenic signaling pathways that regulate ROS homeostasis and bypass ROS-evoked apoptosis in MPM are poorly understood. In this study, we demonstrate that the mitogen-activated protein kinase (MAPK) pathway RAS-RAF-MEK-ERK is hyperactive and a molecular driver of MPM, independent of histological subtypes and genetic heterogeneity. Suppression of MAPK signaling by clinically approved MEK inhibitors (MEKi) elicits PARP1 to protect MPM cells from the cytotoxic effects of MAPK pathway blockage. Mechanistically, MEKi induces impairment of homologous recombination (HR) repair proficiency and mitochondrial metabolic activity, which is counterbalanced by pleiotropic PARP1. Consequently, the combination of MEK with PARP inhibitors enhances apoptotic cell death in vitro and in vivo that occurs through coordinated upregulation of cytotoxic ROS in MPM cells, suggesting a mechanism-based, readily translatable strategy to treat this daunting disease. Collectively, our studies uncover a previously unrecognized scenario that hyperactivation of the MAPK pathway is an essential feature of MPM and provide unprecedented evidence that MAPK signaling cooperates with PARP1 to homeostatically maintain ROS levels and escape ROS-mediated apoptosis.

## Background

Malignant pleural mesothelioma (MPM) is a deadly cancer caused by asbestos exposure. To date, there are no targeted therapies and few effective treatment options, with chemotherapy being the standard of care for most MPM patients with advanced disease that only modestly improves clinical outcomes [1–3]. Combined immunotherapy targeting the immune checkpoints programmed cell death 1 (PD-1) and cytotoxic T-lymphocyte antigen 4 (CTLA-4) has recently been approved as first-line treatment, but this new modality has shown promising clinical efficacy in only a small proportion of patients [1, 2]. Therefore, the search for new therapeutic targets and strategies for MPM remains an unmet goal.

Mesotheliomas have three phenotypically distinct histologic subtypes: epithelioid (50 to 60% of cases), sarcomatoid (10% of cases), and biphasic (30 to 40% of cases), a mosaic of epithelioid and sarcomatoid subtypes. However, this classification provides little insight into molecular features and treatment options for discrete subclasses [1–5]. Systematic genomic studies have revealed that the mutational landscape of MPM is dominated by the loss of function alterations in tumor suppressor genes (TSGs), e.g., *BAP1*, *CDKN2A*, *NF2* [6–8]. Notably, studies using genetically engineered mouse models have shown that simultaneous inactivation of multiple TSGs is required to trigger sporadic MPM in mice [9, 10], leaving open question of which signaling pathway is essential for the disease development.

RAS and mitogen-activated protein kinase (MAPK) cascade RAF-MEK-ERK (RAS/MAPK pathway) is one of the most prevalent oncogenic signaling pathways dysregulated and hyperactivated in human cancers, primarily by genetic alterations in RAS and RAF [11, 12]. Although activating mutations along the MAPK pathway are absent or rare [6, 7], it is noteworthy that the pathogenic potential of asbestos, an indisputable precipitant of mesothelioma [13–15], is associated with stimulation of MAPK signaling [16] and that MPM cell lines exhibit activation of the RAS/MAPK pathway [17]. Recent studies examining large cohorts or using alternative approaches show that sporadic and recurrent gain-of-function mutations in RTKs (e.g., EGFR) and RAS (e.g., KRAS) affect a significant proportion of MPM patients [17–21]. Collectively, these observations suggest that the RAS/MAPK pathway may play a role in MPM that has not been adequately investigated.

Strategies targeting the MAPK pathway have been intensively pursued in RAS- and RAF-driven cancers [11, 12]. However, MAPK-targeted therapies, such as the clinically approved MEK inhibitor (MEKi) trametinib, are only marginally effective due to various protective mechanisms, *de novo* and/or acquired during treatment [11, 12]. Accordingly, additional targets are needed to maximize MEKi activity. Poly (adenosine diphosphate–ribose) polymerase 1 (PARP1) is a key component of single-strand break (SSB) repair machinery and a synthetic lethal target in tumors defective in homologous recombination (HR)-dependent DNA damage repair (DDR) [22]. Mechanistically, blocking PARP1 activity induces SSBs, which are converted to double-stranded breaks (DSB) during DNA replication and leads to synthetic lethality in HR-deficient cancer cells. Previous studies have also shown that PARP inhibitors are a potential targeted therapy for MPM [23, 24]. While *BRAC1/2* mutations are rare [6, 7, 25], BRCA1-associated protein 1 (encoded by *BAP1*) is altered in a substantial subset of MPM patients [6–8]. Despite a possible role of BAP1 in HR [25–27], it is still unclear whether *BAP1* mutations cause PARP1 dependency in MPM [24, 28, 29]. In addition to its canonical function in DNA damage repair (DDR), there is growing evidence that PARP1 is also involved in other biological processes such as oxidative stress response and mitochondrial homeostasis [30, 31], as well as regulation of cell metabolism [32]. Intriguingly, asbestos carcinogenesis is associated with mutagenic reactive oxygen species (ROS) that promote chronic inflammation and DNA damage [13–15]. Because excess ROS are harmful, cancer cells must leverage ROS levels to favor tumor progression but avoid ROS-driven cell death [33, 34]. In this context, we and others have shown that autophagy, a recycling process that removes oxidized cellular components and regulates cellular ROS content, plays a critical role in MPM [35, 36]. Although PARP1 may regulate autophagy [37], it is unclear whether and how PARP1 is involved in ROS metabolism and the prevention of ROS-dependent cell death in MPM.

Here, we perform integrated analyses of multi-omics datasets of patient tumors and report the unexpected finding that the RAS/MAPK signaling pathway is hyperactive and a molecular driver of MPM. Furthermore, we show that inhibition of the pathway by MEKi elicits a protective mechanism involving the pleiotropic PARP1, and that the combination of PARPi and MEKi enhances MPM cell death in vitro and in vivo. Mechanistically, we show that concurrent PARPi treatment amplifies MEKi-induced HR deficiency and metabolic dysfunction, and that MEKi/PARPi cytotoxicity is due to the coordinated production of excess ROS. Taken together, our studies demonstrate that hyperactivation of the RAS/MAPK pathway is a cardinal feature of MPM and reveals a novel mechanism that maintains ROS homeostasis and escapes ROS-driven apoptosis in MPM.

## Results

### The MAPK pathway is hyperactive and a molecular driver of MPM cells

**Fig. 1:**
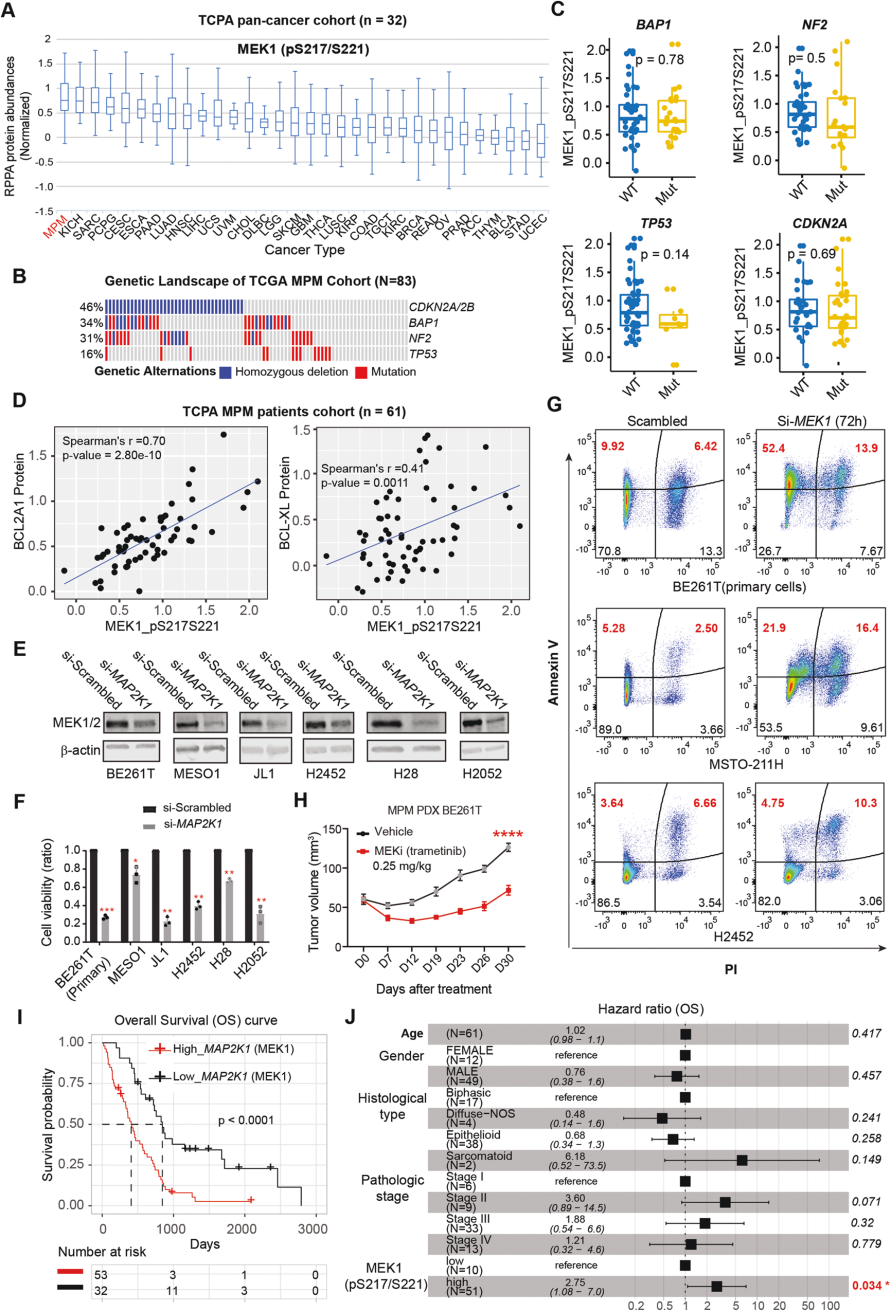
The MAPK pathway is hyperactive and an oncogenic dependency in MPM. **A** p-MEK1 (Ser217/221) protein levels in The Cancer Genome Atlas (TCGA) pan-cancer cohort (*n* = 32). The protein expression profile [reverse-phase protein array (RPPA)] was downloaded from The Cancer Proteome Atlas (TCPA) portal (https://tcpaportal.org/). The x-axis represents the cancer types and is ordered by mean p-MEK1 (Ser217/221) protein level. **B** Genetic alterations of tumor suppressor genes (TSG) in the TCGA cohort of malignant pleural mesothelioma (MPM) patients (*n* = 83). Data were downloaded from the cBioPortal for Cancer Genomics (https://www.cbioportal.org/). The blue color represents homozygous deletion of the indicated genes, while the red color indicates point mutations. **C** Histogram plots showing the difference in p-MEK1 protein levels between MPM samples from a cohort of patients in TCGA stratified by the indicated mutations. Notably, the p-MEK1 protein levels are independent of the mutational status of TSGs (*BAP1*, *NF2*, *TP53*, and *CDKN2A*). The p-value was determined by Welch’s *t*-test. **D** p-MEK1 (Ser217/221) protein level is positively correlated with anti-apoptotic proteins (BCL2A1, BCL-XL) in MPM patients. Proteomic data of patient MPM tumors were downloaded from the TCPA database. **E** Immunoblots of a panel of MPM cells treated with siRNA-based *MAP2K1* (encoding MEK1) knockdown. **F** Viability assay of the indicated MPM cells transfected (72 h) with *MAP2K1*-specific or control siRNAs. *p < 0.05, **p < 0.01, ***p < 0.001 by Welch’s *t*-test (*n* = 3). In MESO-1 cells, both *MAP2K1* and *MAP2K2* were knocked down by siRNAs. **G** Flow cytometry-based apoptotic analysis of MPM cells after 72 h transfection with MEK1-specific siRNAs (si-MEK1). The Q1 and Q2 populations (in red) are considered apoptotic cells. The percentage of early and late apoptotic cells, defined by Annexin V^+^/PI^-^ and Annexin V^+^/PI^+^ populations, respectively, were highlighted in red. **H** In vivo efficacy of trametinib (0.25 mg/kg) in patient-derived xenograft (PDX) BE261T (5 mice/group). Data were shown as mean ± SEM, with ****p < 0.0001 by two-way ANOVA. **I**, **J** Kaplan–Meier univariate survival (**H**) and multivariate Cox regression (**I**) analyses of the TCGA cohort of MPM patients. Patients are dichotomized by the optimal cutoff value of *MAP2K1* (*n* = 85) or the p-MEK1 (*n* = 61) level across all patients, with survival curves and cumulative hazard rates analyzed and plotted using the R ‘survival’ and ‘survminer’ packages. The *p*-value was calculated using the log-rank test.

To identify cellular processes representing selective vulnerabilities in MPM, we interrogated the TCPA reverse-phase protein array (RPPA) dataset that quantitatively profiled 220 cancer-related proteins across a large cohort of patients [38]. Among the pan-cancers (*n* = 32) studied, MPM tumors from a cohort of 61 patients (*n* = 61) show the highest average protein level of p-MEK1 (Ser217/221), a marker of RAS/MAPK pathway activity (Fig. 1A), although its distribution in MPM samples is heterogeneous, suggesting that MEK1 activation occurs at least in at least a subset of MPM tumors. Of note, the high p-MEK1 level in MPM tumors is independent of their alterations in TSGs (e.g., *CDKN2A*, *BAP1*, *NF2*, *TP53*) (Fig. 1B, C). Interestingly, p-mTOR (Ser2448), a marker of mTOR pathway activity, is expressed at a low average level in MPM compared with other cancers (Fig. S1A), whereas p-RICTOR (Thr1135), an mTORC2 inhibitor and part of a feedback mechanism that negatively regulates AKT/mTORC1, is expressed at the second highest level in the pan-cancer cohort, just after sarcoma (SARC) (Fig. S1B). This observation is consistent with previous studies indicating that deregulation of mTOR signaling occurs primarily in the sarcomatoid subtype of MPM [6, 7] and that mTOR inhibitors had limited clinical activity in an unselected cohort of MPM patients [39].

Further analysis of RPPA proteomic data of MPM patients (*n* = 61) revealed that p-MEK1 protein level correlates strongly with many other cancer proteins that regulate malignant processes and are important hallmarks of cancer (Table S4). In particular, BCL2A1 (BCL-2-related protein A1) and BCL-XL (anti-apoptosis), SNAI1, Fibronectin, and N-Cadherin [epithelial-to-mesenchymal transition (EMT)], RAD51, MRE11, and p-CHK2 (Thr68) in HR repair are significantly positively correlated with p-MEK1 (Fig. 1D; Fig. S1C, D). Knockdown of *MAP2K1* (encoding MEK1) alone or in combination with *MAP2K2* (MEK2) significantly, although to varying degrees, inhibited proliferation of a panel of histologically and genetically different MPM cells and BE261T, a primary MPM cell culture [36, 40] (Fig. 1E, F; Table S1), paralleled by the induction of apoptotic cell death and cell-cycle arrest (Fig. 1G; Fig. S1E**)**. In support of the in vitro results, MEK inhibition with trametinib substantially suppressed tumor growth in MESO-1 and patient-derived xenograft (PDX) models without apparent toxicities in mice (Fig. 1H; Fig. S1F, G). The clinical relevance of our findings was assessed in the TCGA cohort of MPM patients: high MEK activity (high *MAP2K1 mRNA* or p-MEK1 protein level) is strongly associated with poor patient survival regardless of MPM histology and stages (Fig. 1I, J). These findings uncover a previously unrecognized scenario in which MAPK signaling is hyperactive and affords an important cancer dependency in MPM cells.

### Molecular mechanisms underlying MAPK hyperactivation in MPM

**Fig. 2:**
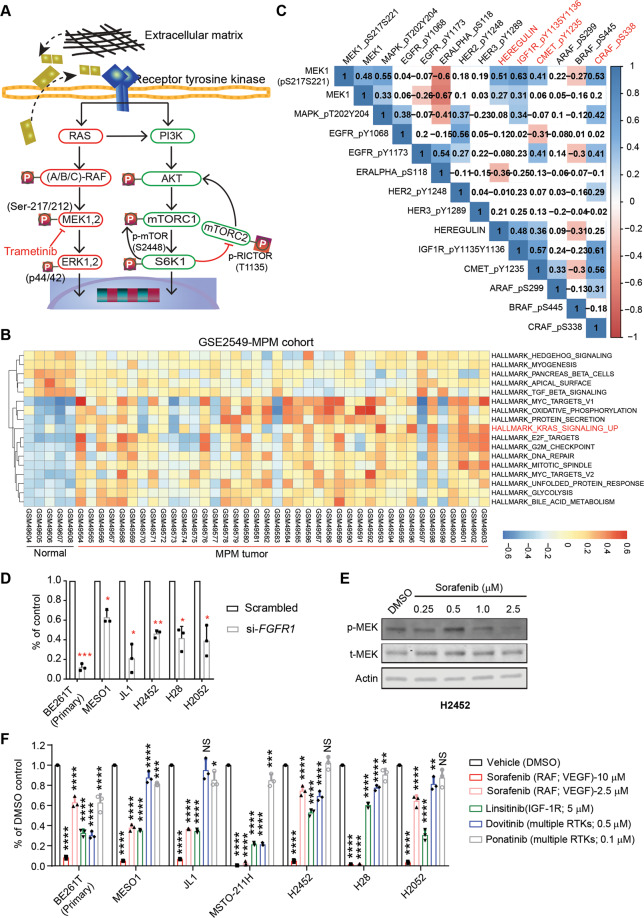
Molecular mechanisms underlying MAPK hyperactivation in MPM. **A** Schematic of the RAS-RAF-MEK-ERK and the mTOR pathway downstream of RTK signaling. **B** Gene set variation analysis (GSVA) shows deregulation of the Hallmark modules in MPM tumors compared with normal pleural tissue. The transcriptomic data of MPM samples (GSE2549 dataset) were downloaded from the Gene Expression Omnibus (GEO). Note that the KRAS_signaling_UP signature is significantly enriched in MPM tumors vs. normal pleural tissue. **C** The RTK pathway proteins (p-IGF1R, p-CMET, HEREGULIN) and the MAPK pathway proteins (p-CRAF, p-MAPK/p-ERK) correlate strongly and positively with p-MEK1 (Ser217/221) in MPM. The numbers in the correlogram indicate the correlation coefficient (Spearman), with significant (*P* < 0.05) positive and negative correlations shown in blue and red, respectively. The color intensity is proportional to the correlation coefficient, with a non-significant (*P* > 0.05) correlation in the blank background. **D** Viability assay of the indicated MPM cells transfected (72 h) with *FGFR1*-specific or control siRNAs. *p < 0.05, **p < 0.01, ***p < 0.001 by Welch’s *t*-test (*n* = 3). **E** Immunoblots of H2452 cells treated for 12 h with the B/C-RAF inhibitor sorafenib. Note that 2.5 µM sorafenib effectively blocked MEK signaling (decrease in p-MEK). **F** Viability assay of the indicated MPM cells treated for 96 h with clinically relevant doses (maximal plasma concentration in patients) of the indicated inhibitors. Data are shown as mean ± s.d. (*n* = 3). *p < 0.05, **p < 0.01, ***p < 0.001, ****p < 0.0001 by Welch’s *t*-test.

We next investigated the molecular basis of MAPK hyperactivation in MPM. As mutations in RAS and RAF, a common mechanism abnormally activating MEK [11, 12], are infrequent in MPM [6, 7], non-mutational mechanisms may play a key role in MEK hyperactivation (Fig. 2A). Notably, gene set variation analysis (GSVA) of the GEO data (GSE2549) showed significant enrichment of the KRAS_signaling_UP signature (genes upregulated by KRAS activation) in patient MPMs compared with normal pleural tissue (Fig. 2B). In MPM patients, p-MEK1 protein level correlates positively and strongly with the upstream p-CRAF (Ser338) and downstream p-ERK1/2 (MAPK_pT202Y204), and vice versa (Fig. 2C). The interplay between MEK1 and CRAF rather than A/B-RAF is reminiscent of the well-defined scenario in RAS-driven cancer that CRAF (RAF1) transduces signals from RTKs to MEK [41]. These observations reveal a signaling axis through CRAF-MEK-ERK in MPM and suggest that MPM tumors, like RAS-driven cancer, are characterized by hyperactive RAS/MAPK signaling.

Pathway enrichment analysis indicated that p-MEK1 protein level in MPM patients is most significantly correlated RTK signaling (Fig. S2A), with p-IGF1R (Tyr1135/1136), p-MET (Tyr1235), and HEREGULIN (a cognate ligand of ERBB3/4) strongly and positively correlated with CRAF, p-MEK1, and p-ERK1/2 (Fig. 2C). Consistently, several RTKs and their ligands, e.g., FGFR1/FGF and insulin growth factor (IGF) signaling (i.e., IGF2R, IGF1, IGFBP), are overexpressed (adjusted *p*-value < 0.05) in MPM tumors compared with normal pleura in distinct clinical cohorts (Fig. S2B). Since not all RTKs were covered by RPPA study [38], we examined Cancer Dependency Map Project, which systematically analyzed genetic vulnerabilities of cancer cells including MPM cell lines (*n* = 18), and identified *FGFR1* as an essential gene for MPM (Fig. S2C, D). We confirmed that MPM cells depend exquisitely on FGFR1 for survival, as its depletion significantly impairs MPM proliferation (Fig. 2D). Supporting the functional importance of RTK signaling for MPM, analysis of drug sensitivity data in GDSC showed that MPM cells (*n* = 14) were generally sensitive (low IC_50_ Z-score) to pan- or specific RTK inhibitors, e.g., ponatinib (FGFR, PDGFR, VEGFR), GSK1904529A (IGF1R), PD173074 (FGFR), albeit to varying degrees (Fig. S3).

To further validate the role of RTKs and CRAF in MAPK hyperactivation, we treated MPM cells with clinically relevant doses [maximal plasma concentrations (C_max_)] of the CRAF inhibitor sorafenib (C_max_: 20.1 µM), IGF-1R inhibitor linsitinib (C_max_: 6.857 µM), and the FGFR inhibitor dovitinib (C_max_: 0.471 µM) and ponatinib (C_max_: 0.137 µM). At the dose of ½C_max_, sorafenib effectively decreased p-MEK (Fig. 2E) and potently inhibited MPM proliferation (Fig. 2F), as did linsitinib, while dovitinib and ponatinib exhibited cell-specific efficacy (Fig. 2F). Thus, CRAF and RTKs (e.g., IGF-1R, FGFR1) are important upstream factors for MEK activation in MPM. Supporting our results, MPM cells are highly secretory and autocrine/paracrine-mediated deregulation of RTK signaling prevails in MPM [42, 43].

### MEK inhibition elicits genomic susceptibility to PARP1 blockage

**Fig. 3:**
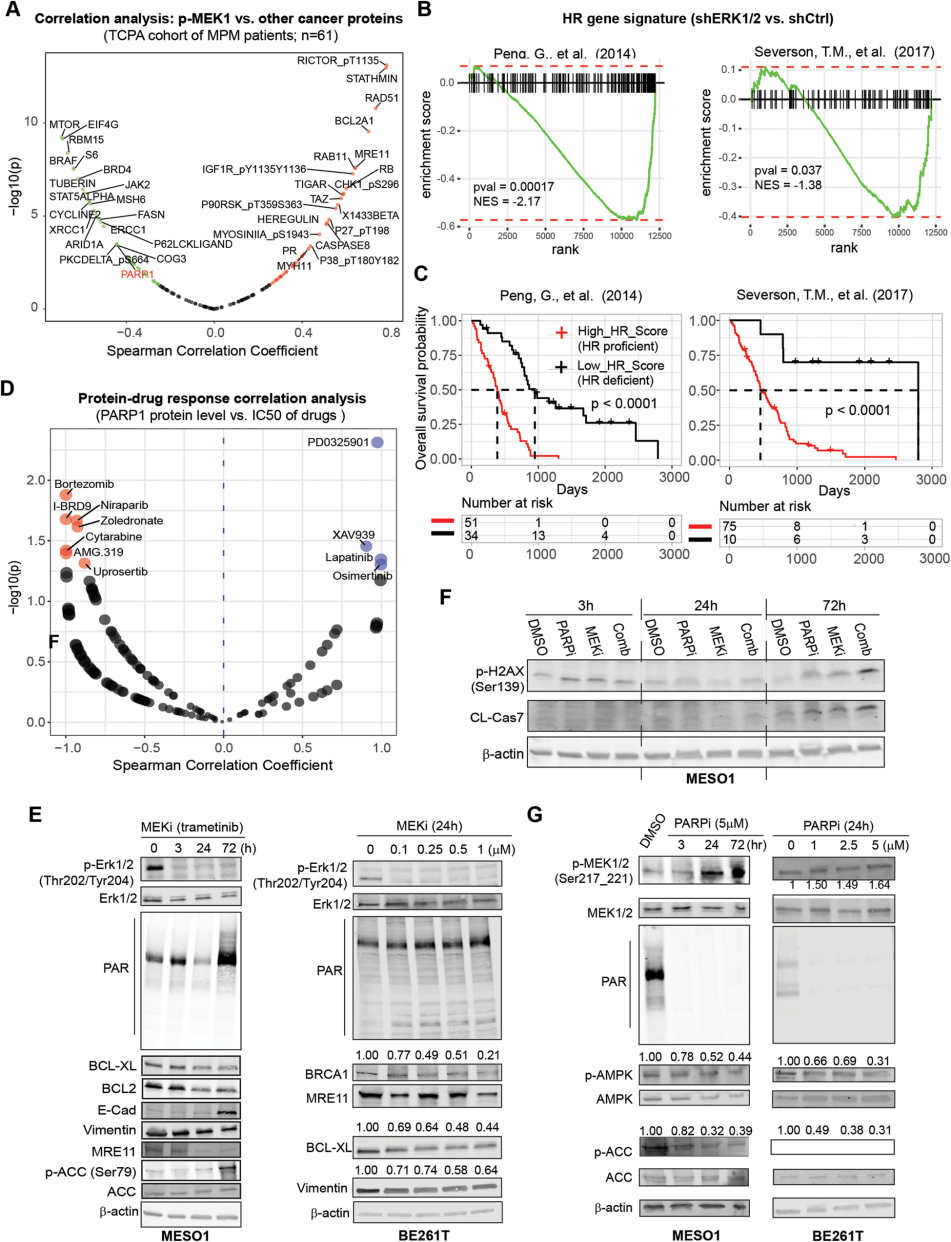
MEK inhibition impairs HR and evokes protective PARP in MPM cells. **A** Proteins and phosphoproteins whose expression is significantly correlated with p-MEK1 (Ser217/221) protein level in MPM. The green dots indicate the proteins significantly (*p* < 0.05) negatively correlated with p-MEK1, and the red significantly positively correlated with p-MEK1. The proteins with a correlation threshold (Spearman’s correlation coefficient >0.4 or <−0.4) are marked. RPPA proteomic data of MPM patients were downloaded from TCPA. **B** Gene set enrichment analysis (GSEA) revealed significant downregulation of homologous recombination (HR) gene signature in ERK1/2-depleted MPM cells. The GSE21750 dataset from the Gene Expression Omnibus (GEO) portal was used for the analysis, with pre-defined HR gene signatures based on Peng et al. (2014) and Severson et al. (2017). **C** Kaplan–Meier univariate survival analyses of MPM patients (*n* = 87). Algorithm-based HR signatures are based on the indicated studies. MPM patients are stratified by the optimal cutoff value of the HR score across all patients using the surv_cutpoint function in the R ‘maxstat’ package. The *p*-value is calculated by the log-rank test. **D** PARP1 protein level predicts MEKi sensitivity. The blue dots are inhibitors whose IC_50_ values are significantly (*p* < 0.05) positively correlated with PARP1 levels, and the red ones are significantly (*p* < 0.05) negatively correlated with PARP1. The correlation analysis was based on the drug sensitivity profile of thoracic cancer cells (*n* = 32, including 3 MPM cell lines and 29 lung cancer cell lines) in GDSC. **E** Immunoblots of MESO-1 and BE261T cells treated with MEKi (trametinib; 0.5 μM) for the indicated time (MESO1) or for 24 h with different concentrations (BE261T). Protein quantification was shown above the protein bands. **F** Immunoblots of MESO-1 cells treated with trametinib (MEKi; 0.5 µM) and olaparib (PARPi; 5 µM), alone and in combination for the indicated time. **G** Immunoblots of BE261T and MESO-1 cells treated with olaparib (PARPi) for the indicated time. Protein quantification was shown above the bands.

MEKi monotherapy is ineffective or short-lived in RAS/MAPK-driven cancer [11, 12]. Analogously, trametinib alone delayed but failed to arrest MPM growth in PDX models (Fig. 1H), suggesting the presence of protective mechanisms that limit MEKi efficacy. To identify potentially defective pathways and vulnerabilities in MEKi-treated MPM cells, we analyzed RPPA proteomic data of MPM patients [38] and transcriptomes of MPM cells (i.e., GSE21750) [44]. p-MEK1 protein level in MPM patients positively correlates with HR pathway proteins (RAD51, MRE11) but negatively with SSB pathway proteins (PARP1, ERCC1, XRCC1, MSH6) (Fig. 3A; Fig. S1D). Using predefined HR gene sets [45, 46], we found that inhibition of RAS/MAPK signaling in MPM cells by genetic knockdown of ERK1/2 profoundly suppressed the transcriptional signatures of HR (Fig. 3B), which are predictive markers of poor prognosis in MPM patients (Fig. 3C). These results suggest that the RAS/MAPK pathway promotes the transcription of HR genes in MPM and MEKi-treated MPM cells possess HR defects.

The opposing nature of HR and SSB in terms of their relationship to p-MEK1 in MPM (Fig. 3A; Fig. S1D) also suggests that SSB repair pathway may compensate MEKi-induced HR defects. We therefore examined drug response data in GDSC and found that the sensitivity (IC_50_) of thoracic cancer cells (including MPM cells) to the MEK inhibitor PD0325901 is most strongly and inversely associated with protein levels of PARP1, a key factor in SSB pathway: cancer cells with high levels of PARP1 are more resistant to MEKi (Fig. 3D). Indeed, MPM cells (MESO-1, BE261T) treated with trametinib (MEKi) showed an overt decrease in HR proteins (BRCA1, MRE11) but marked increase in poly-ADP-ribosylation (PAR), the product of PARP and a biomarker of sensitivity to PARPi (Fig. 3E; Fig. S4A). As in MPM and reinforcing the analogy of MPM to RAS/MAPK-driven cancer, MEKi has been shown to attenuate HR activity in RAS- and RAF-mutant melanomas [47]. Notably, MEKi promoted apoptosis (decrease in BCL-XL and BCL2) and reverted EMT (decrease in the mesenchymal marker Vimentin, increase in the epithelial marker E-Cadherin) in MESO-1 cells (Fig. 3E), in line with our genetic study (Fig. 1F, G) and in support of our finding that MEK hyperactivation is a molecular driver of MPM. Importantly, PARPi exacerbated MEKi-induced HR defects and apoptosis in MESO-1 cells, as indicated by a time-dependent increase in γ-H2AX and caspase 7 cleavage (Fig. 3F). Therefore, MEKi induces impairment of HR proficiency, which is compounded by concomitant PARPi treatment.

**Fig. 4:**
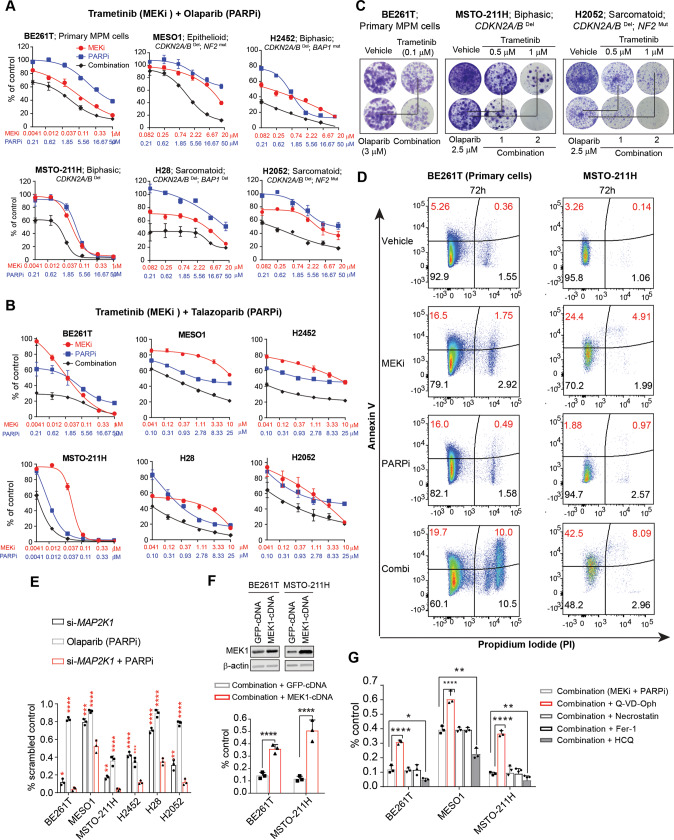
The combination of MEKi/PARPi enhances the apoptotic death of MPM cells. **A** Dose-response curves of MPM cells treated for 72 h with trametinib (MEKi) and olaparib (PARPi), alone or in combination. Data are shown as mean ± s.d. (*n* = 3). **B** Dose-response curves of the indicated MPM cells treated with trametinib and talazoparib (PARPi). Data are shown as mean ± s.d. (*n* = 3). **C** Clonogenic assay of MPM cells (BE261T, MSTO-211H, H2052) treated with trametinib and olaparib, alone or in combination (1–2 weeks). **D** Apoptotic assay of BE261T and MSTO-211H cells treated with MEKi (trametinib; 0.5 μM) and PARPi (olaparib; 5 μM), alone and in combination for 72 h. The percentage of early and late apoptotic cells, defined by Annexin V^+^/PI^-^ and Annexin V^+^/PI^+^ populations, respectively, were highlighted in red. **E** MEK1 knockdown (si-*MAP2K1*) sensitizes MPM cells to PARPi (olaparib). MPM cells transfected with si-scrambled or si-*MAP2K1* (48 h post-transfection) were treated with MEKi/PARPi (trametinib, 0.1 μM; olaparib, 5 μM) for additional 72 h and subjected to viability assay. Scrambled siRNAs were used as control. *p < 0.05, **p < 0.01, ***p < 0.001, ****p < 0.0001 by Welch’s *t*-test (compared with si-*MAP2K1* + PARPi). **F** MEK1 overexpression compromises MEKi/PARPi efficacy. MPM cells transfected with control (GFP) or MEK1-GFP vectors were subjected to immunoblot analysis 48 h post-transfection or viability assay after being treated for 72 h with the MEKi/PARPi combination (trametinib, 0.1 μM; olaparib, 5 μM). ****p < 0.0001 by Welch’s *t*-test. **G** Viability assay of MPM cells treated with trametinib (0.1 µM) and olaparib (5 µM) (combination), in the absence or presence of the indicated inhibitors for 96 h. Q-VD-Oph (20 μM), pan-caspase inhibitor; necrostatin-1 (10 μM), necroptosis inhibitor; Fer-1 (2 μM), ferroptosis inhibitor; HCQ (5 μM), autophagy inhibitor. *p < 0.05, **p < 0.01, ***p < 0.001, ****p < 0.0001 by one-way ANOVA.

Olaparib (PARPi) not only reduced PARP activity (decreased PAR) but also activated MEK (increased p-MEK1) in MPM cells (Fig. 3G), suggesting that MEK and PARP reciprocate and that a combination of MEKi and PARPi may be promising to combat MPM. Indeed, MEKi/PARPi showed synergistic anti-proliferative effects (CI ≤ 1) in MPM cells differing in histology and genetic background (Fig. 4A–C; Fig. S4B, C) and induced significantly higher apoptosis than single agents (Fig. 4D; Fig. 3F). Silencing of MEK1 enhanced PARPi efficacy (Fig. 4E), whereas MEK1 overexpression attenuated MEKi/PARPi cytotoxicity in MPM cells (Fig. 4F). The anti-MPM effect of MEKi/PARPi was impaired by Q-VD-Oph, a pan-apoptosis inhibitor, but potentiated by autophagy inhibition (hydroxychloroquine, HCQ), while blocking ferroptosis (Fer-1) and necrosis (Necrostatin) had no obvious effects (Fig. 4G). Thus, MEKi/PARPi drives apoptotic death of MPM cells and autophagy limits their efficacy. In contrast, MEKi/PARPi showed no synergism in fibroblast cells from normal human lung (Fig. S4D). Collectively, these results indicate that MEKi elicits HR defects and renders genomic susceptibility of MPM cells to PARP blockage.

### PARPi exacerbates MEKi-induced metabolic stress in MPM cells

**Fig. 5:**
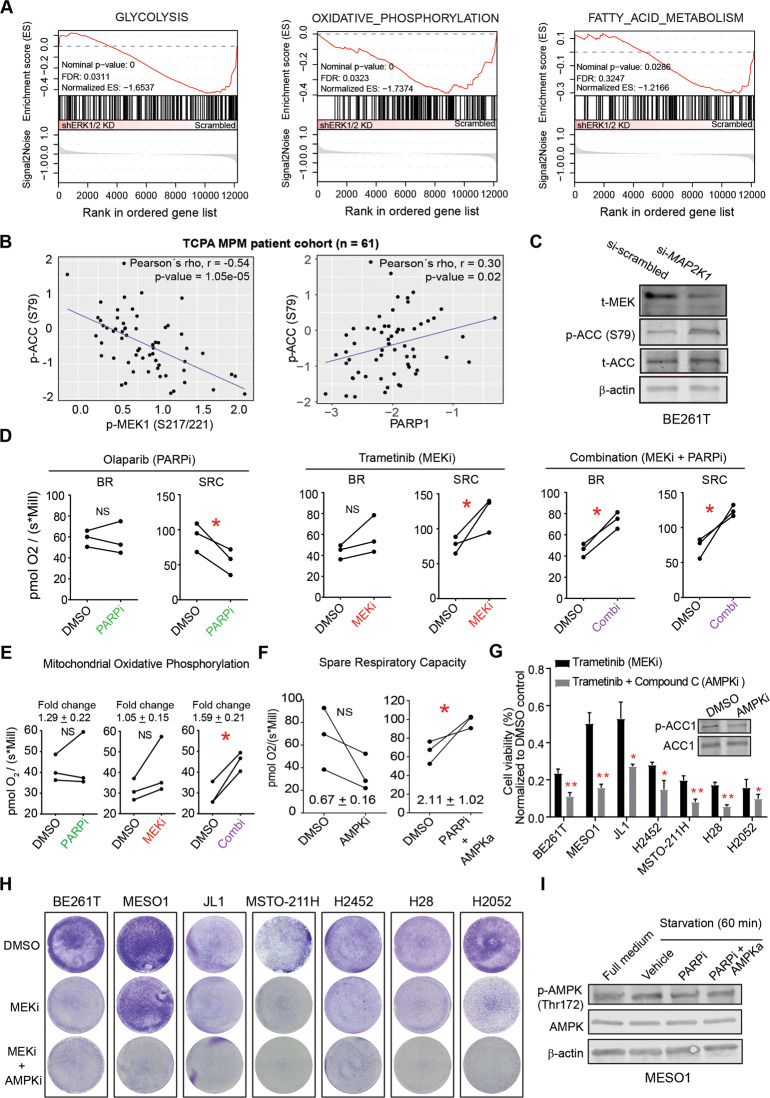
PARP-AMPK-regulated SRC protects MEKi-induced metabolic stress. **A** Gene set enrichment analysis (GSEA) showed that downregulation of ERK1/2 significantly decreased transcriptional signatures of glycolysis, oxidative phosphorylation, and fatty acid metabolism. The GEO dataset GSE21750 was used for GSEA. **B** p-ACC (Ser79) protein level negatively correlates with p-MEK1 but positively with PARP1 in MPM patients. The protein expression data [reverse-phase protein array (RPPA)] were downloaded from The Cancer Proteome Atlas (TCPA) portal (https://tcpaportal.org/). **C** Immunoblots of BE261T cells transfected (72 h) with si-*MAP2K1* or scrambled siRNAs. **D** Respirometric measure (*n* = 3) of basal respiration (BR) and spare respiration capacity (SRC) in MESO1 cells treated with MEKi (0.5 μM trametinib) and PARPi (5 μM olaparib) for 72 h. *p < 0.05 by paired *t*-test. **E** Quantification of mitochondrial oxidative phosphorylation (OXPHOS) in MESO-1 cells treated with DMSO, PARPi, MEKi, and the combination. *p < 0.05 by paired *t*-test (*n* = 3). **F** Quantification of spare respiratory capacity (SRC) in MESO-1 cells treated with DMSO, AMPK inhibitor (AMPKi), and the combination of PARPi and AMPK activator (AMPKa). *p < 0.05 by paired *t*-test (*n* = 3). **G**, **H** Viability (72 h; G) and clonogenic (1 week; H) assay of MPM cells treated with MEKi (0.5 μM), in the presence or absence of compound C (5 μM). Shown in the insert (**G**) are immunoblots of BE261T cells treated with DMSO or compound C for 12 h. *p < 0.05, **p < 0.01, by Welch’s *t*-test. **I** Immunoblots of MESO1 cells starved (60 min) and co-treated (60 min) with compound C (AMPKi; 5 µM), AICAR (AMPKa; 1 mM), and olaparib (PARPi; 5 µM). Quantification of phospho-proteins was normalized to its total protein and the β-actin.

PARP1 is pleiotropic, with functions beyond DDR [32], which prompted us to investigate whether other mechanisms contribute to the observed MEKi/PARPi synergy (Fig. 4; Fig. S4). ERK1/2 silencing in MPM cells suppressed not only HR but also metabolic gene signatures, especially glycolysis, oxidative phosphorylation (OXPHOS), and fatty acid metabolism (Fig. 5A), pointing to a role of RAS/MAPK signaling in MPM metabolism. Supporting this notion, p-MEK1 protein level and phosphorylated acetyl-CoA carboxylase (p-ACC, Ser79) correlate negatively in MPM tumors (Fig. 5B), which was confirmed in MESO-1 cells: pharmacological and genetic inhibition of MEK increased p-ACC (Fig. 3E; Fig. 5C). In contrast to p-MEK1, PARP1 protein level positively correlates with p-ACC (Ser79) in MPM patients (Fig. 5B), and PARPi decreased p-ACC in MESO-1 and BE261T cells (Fig. 3G). Thus, MEK and PARP1 play opposite roles in regulating ACC, further supporting the finding that PARP1 and MEK interact reciprocally in MPM (Fig. 3).

ACC is a key enzyme in fatty acid synthesis and catalyzes a rate-limiting step of this pathway by irreversibly carboxylating acetyl-CoA to generate malonyl-CoA, a building block of fatty acids. ACC phosphorylation at Ser79 inactivates the enzymatic activity of the protein, which is mediated by AMP-activated protein kinase (AMPK). AMPK is a metabolic stress signal transducer activated during energy crises such as starvation or an elevated AMP/ATP ratio and plays a critical role in stress response and adaptation by shutting down energy-intensive anabolic metabolism (e.g., lipid synthesis) and activating autophagy [48]. Of note, PARPi reduced AMPK activity (p-AMPK decrease) (Fig. 3E, G), confirming that PARP1 regulates AMPK activity. These results suggest that MEKi also induces metabolic stress in MPM and initiates a homeostatic mechanism involving the PARP1-AMPK axis.

### PARP1-regulated mitochondrial SRC counteracts MEKi-induced metabolic stress

We wondered whether the mitochondria are involved in MEKi-induced stress response given their central role in metabolic homeostasis [49]. High-resolution respirometry showed that MEKi or PARPi alone only slightly affected basal oxygen consumption rate (OCR), termed basal respiration (BR), in MPM cells, whereas the drug combination significantly increased BR (Fig. 5D; Fig. S5A), as did oxidative phosphorylation (OXPHOS) that was significantly upregulated only by MEKi/PARPi combination (Fig. 5E). Importantly, mitochondrial spare respiratory capacity (SRC), a functional trait that promotes cell survival by avoiding ATP crisis [50], was significantly decreased by PARPi but upregulated by MEKi and the combination (Fig. 5D), pointing to a role of PARP1 in regulating mitochondrial SRC.

We next investigated whether PARP1 regulates mitochondrial SRC through the AMPK. AMPK inhibition by compound C (AMPKi) decreased SRC (Fig. 5F; Fig. S5B) and largely mimicked the action of PARPi in MPM cells, as AMPKi decreased p-ACC and enhanced the anti-proliferative effect of MEKi (trametinib) in MPM cells, despite the neglectable effects of AMPKi alone on total ACC protein or MPM proliferation (Fig. 5G, H; Fig. S5C). In contrast, activating AMPK by AICAR (AMPKa) overcame the inhibitory effect of PARPi on SRC (Fig. 5D), such that concurrent AMPKa/PARPi treatment increased SRC in MPM cells (Fig. 5F; Fig. S5D). Finally, olaparib (PARPi) mitigated starvation-induced AMPK activation (increase in p-AMPK), a well-known mechanism in response to energy stress, but AICAR (AMPKa) reverted the inhibitory effect of PARPi by restoring p-AMPK expression (Fig. 5I). These results suggest that the PARP1-AMPK axis regulates mitochondrial SRC and plays a key role in the surveillance of MEKi-induced metabolic stress in MPM.

### MEKi/PARPi cytotoxicity converges in the coordinated production of excess ROS

**Fig. 6:**
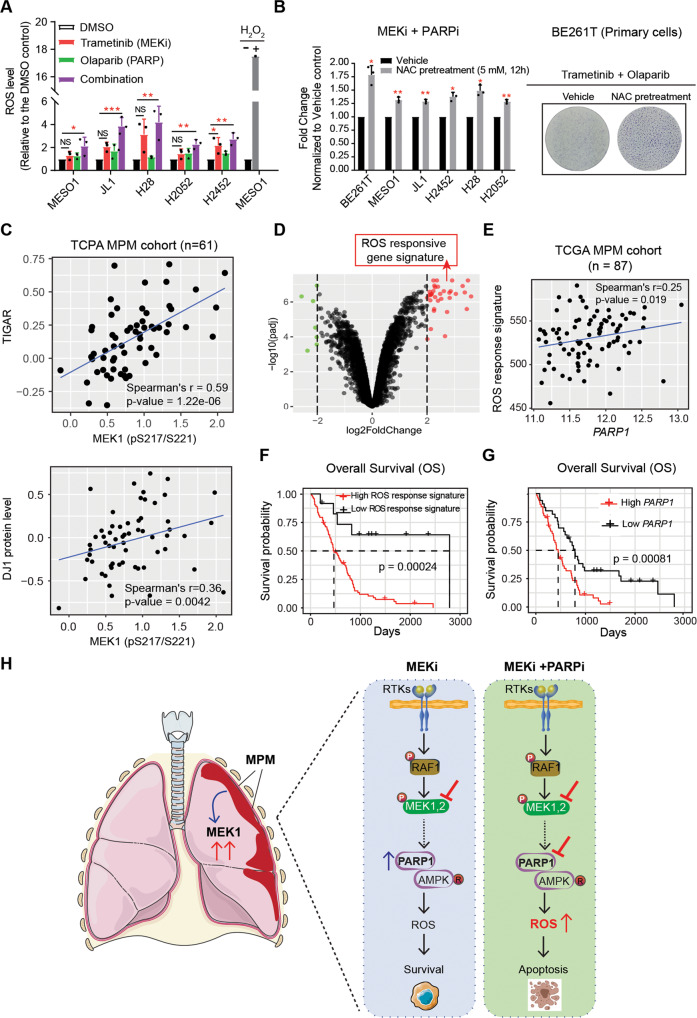
MEKi/PAPRi cytotoxicity converges in the upregulation of excess ROS. **A** Intracellular reactive oxygen species (ROS) levels of MPM cells treated for 72 h with DMSO (vehicle), PARPi (5 µM), and MEKi (0.5 µM), alone and in combination. Data were normalized to vehicle control and shown as mean ± SEM. *p < 0.05, **p < 0.01, ***p < 0.001 by Welch’s *t*-test (comparisons between treatment groups and vehicle control). MESO-1 cells treated with H_2_O_2_ (1 mM; 30 min) were used as the positive control. **B** *N*‑acetylcysteine (NAC) attenuates MEKi/PARPi cytotoxicity. Viability assay of MPM cells pre-incubated (12 h) with DMSO (vehicle) or NAC (5 mM) followed by further treatment (96 h) with trametinib (0.5 µM) and olaparib (5 µM). Data are shown as fold changes, with the viability of the cells treated with trametinib/olaparib/DMSO (vehicle) set as 1. *p < 0.05, **p < 0.01 by Welch’s *t*-test (*n* = 3). Representative clonogenic assay of BE261T cells treated with MEKi (0.1 µM) and PAPRi (5 µM) with or without NAC (5 mM) was shown to the right. **C** p-MEK1 (Ser217/221) protein level is significantly positively correlated with TIGAR and DJ1. The protein expression profile [reverse-phase protein array (RPPA)] was downloaded from The Cancer Proteome Atlas (TCPA) portal (https://tcpaportal.org/). **D** Volcano plots showing the genes downregulated [adjusted *p*-value (padj) <0.05 & log_2_ FC < -2] (in green) and upregulated (padj < 0.05 & log_2_ FC > 2) (in red) by H_2_O_2_, with the H_2_O_2_-upregulated genes determined as ROS responsive signature. The transcriptome dataset (GSE32335) of H_2_O_2_-treated cells was downloaded from the GEO. **E** *PARP1* mRNA levels are significantly positively correlated with the ROS-responsive gene signature in MPM tumors. **F**, **G** High levels of *PARP1* and ROS-responsive gene signature are significantly associated with poor survival in MPM patients. The *P* value is calculated by using the log-rank test. **H** Working model illustrating the results of this study. MEK hyperactivation is the molecular driver and a therapeutic target in MPM. MEKi evokes genomic and metabolic stress and triggers a protective mechanism involving the pleiotropic PARP1. A combination of MEK and PARP inhibitors leads to synergistic upregulation of ROS, which translates into incurable genomic and metabolic perturbations and subsequently apoptotic cell death.

Our findings that PARP1 counteracts MEKi-induced genomic and metabolic stress suggest that MEKi/PARPi cytotoxicity in MPM may result from sustained stress stimuli that cannot be remediated. As MAPK signaling regulates MPM metabolism (Fig. 5A) and MEKi/PARPi increased mitochondrial oxidative phosphorylation (Fig. 5E), a major source of intracellular ROS, we asked whether the combination treatment coordinately affects ROS production. MEKi (0.5 µM) or PARPi (5 µM) showed no or marginal effects on ROS levels, but the drug combination significantly increased ROS in all MPM cells tested (Fig. 6A). This increase was accompanied by the accumulation of DNA damage (γ-H2AX) (Fig. 3F) and induction of apoptosis (Fig. 4D). Importantly, the addition of N-acetyl cysteine (NAC), a ROS scavenger, attenuated MEKi/PARPi effects on MPM cells (Fig. 6B), confirming that ROS upregulation plays a causative role in MEKi/PARPi cytotoxicity against MPM cells.

The p-MEK1 protein level in MPM patients correlated positively with TIGAR and, to a lesser degree, with DJ1 (Fig. 6C), key factors with well-documented functions in metabolic stress response and ROS protection [51, 52], supporting a role for MEK and PARP1 in surveillance of metabolic ROS. Moreover, the ROS-responsive gene signature [genes upregulated by H_2_O_2_ (log_2_ FC > 2, adjusted p-value < 0.05); Table S5] determined from the transcriptome of H_2_O_2_-treated cancer cells (GSE32335) contains TIGAR and correlates positively with *PARP1* transcription in MPM tumors (Fig. 6D, E). Both ROS gene set and *PARP1* mRNA levels are of prognostic value in MPM and allow stratification of subgroups of patients with poor survival (Fig. 6F, G). These results suggest that MEK and PARP1 homeostatically maintain ROS levels and that MEKi/PARPi disrupts the surveillance mechanism, leads to an excess of cytotoxic ROS, and drives apoptosis of MPM cells (Fig. 6H).

### MEKi/PARPi enhances MPM cell death in vivo

**Fig. 7:**
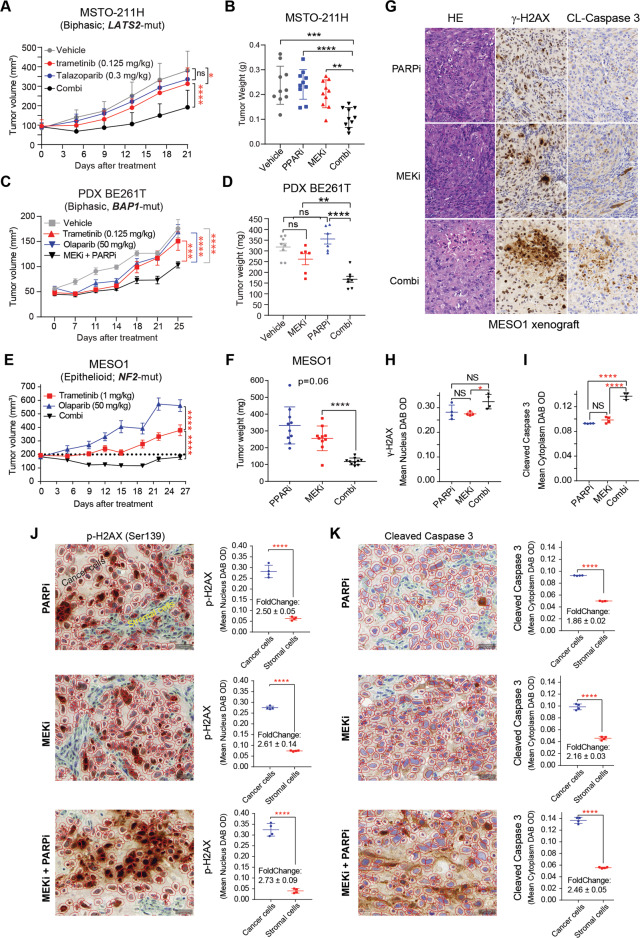
Combined MEKi/PARPi enhances DNA damage and apoptotic death of MPM cells in vivo. **A–F** Growth curves (**A**, **C**, **E**) and tumor weights (**B**, **D**, **F**) of MSTO-211H and MESO-1 xenografts and a patient-derived xenograft (PDX, BE261T) treated with MEKi (trametinib), PARPi (talazoparib or olaparib), alone and in combination. Data are presented as mean ± SEM. *p < 0.05, ****p < 0.0001 by two-way ANOVA. NS non-significant. Note that the efficacy of trametinib (1 mg/kg) as monotherapy has been assessed in MESO-1 xenografts (Fig. S1F), so the vehicle was not repeated here according to the 3 R principle of animal experimentation. **G–I** H&E and IHC (γ-H2AX and Caspase-3) of residual tumors from MESO1 xenografts (**G**), with quantifications of γ-H2AX and Caspase-3 levels shown (**H**, **I**). *p < 0.05, ****p < 0.0001 by two-way ANOVA. Original overall magnification, × 50. **J**, **K** IHC staining and quantification of γH2AX (**J**) and cleaved Caspase 3 (**K**) in residual MESO-1 xenografts after being treated with PARPi, MEKi, and the combination. Four tumors/group were randomly chosen for the analysis, with the quantification of γ-H2AX (nucleus OD value) and cleaved caspase 3 (cytoplasm OD value) based on the whole tissue slide. Representative images (200x) showing the artificial intelligence-based detection of cancer cells and the stroma marked with red and blue circles, respectively, by the QuPath software implementation. ****p < 0.0001 by Welch’s *t*-test.

Finally, we examined the effect of MEKi/PARPi in vivo. FDA-approved trametinib (MEKi) plus olaparib or talazoparib (PARPi) showed significantly higher anti-tumor efficacy than the individual drugs in MESO-1, MSTO-211, H2452 xenografts, and BE261T PDX models (Fig. 7A–F; Fig. S6A–F). Importantly, the residual tumors of the combination group exhibited significantly higher levels of DNA damage (γ-H2AX) and apoptosis (Caspase-3) than the monotherapy groups (Fig. 7G–I), and apoptotic death occurred mainly in the cancer cells rather than in the stroma (Fig. 7J, K). These results support our in vitro data and confirm that the cytotoxicity of MEKi/PARPi in MPM cells is mediated by the induction of apoptosis.

## Discussion

In this study, we provide novel evidence that the RAS/MAPK pathway is hyperactive and an important molecular driver of MPM that transcends tumor histology and genetic heterogeneity. We further show that inhibition of RAS/MAPK signaling by MEKi evokes genomic and metabolic stresses that are protected by pleiotropic PARP1. Finally, we show that a combination of MEKi/PARPi promotes MPM cell death in vitro and in vivo through coordinated suppression of HR repair capacity and mitochondrial metabolic activity resulting from an abnormal production of cytotoxic ROS, validating a mechanism-driven therapeutic strategy for this disease. Because MEKi and PARPi are clinically approved drugs, the rational drug combination warrants clinical investigation in MPM patients for whom there are very few effective therapeutic options. Taken together, our studies provide unprecedented evidence that oncogenic hyperactivation of the RAS/MAPK pathway is a key feature of MPM and that MAPK and PARP1 work together to maintain ROS homeostasis and escape ROS-induced apoptosis in this disease.

MPM features a difficult-to-target genome with a dominant prevalence of loss of function in TSGs [1–3]. The RAS/MAPK pathway, dysregulated in ~40% of human cancers [11, 12], is infrequently genetically altered in MPM [6, 7], which has made this pathway inaccessible for functional studies [1–3]. We now show that RAS/MAPK pathway activity (characterized by p-MEK1) is high in MPM patients and represents an oncogenic dependency of all MPM cells we tested, assigning this pathway a previously unrecognized function in MPM. In support of our findings, asbestos has been reported to activate MAPK signaling and MPM cells have high MAPK activity [16, 17]. Recent studies reveal that recurrent mutations in RTKs and KRAS are more common in MPM patients than previously reported [18–20]. Moreover, Merlin (encoded by *NF2*), a RAS antagonist, is inactivated in a substantial subset of MPM [6, 7], which activates RAS/MAPK signaling. Finally, we show that several RTK, particularly IGF-1R and FGFR1, also contributes to MAPK hyperactivation in MPM.

Our results uncover a reciprocal interplay of MEK and PARP1 and validate that combined MEKi/PARPi is a rational strategy to combat the disease. This finding is supported by a preponderance of the evidence: i) p-MEK1 and PARP1 protein levels correlate in MPM patients; ii) MEKi activates PARP1 and vice versa in MPM cells; and iii) MEKi sensitizes MPM cells to PARPi in vitro and in vivo. Thus, blocking MEK activity unmasks collateral susceptibility and affords a therapeutic window for PARPi in MPM. This finding is reminiscent of the recent report that PARPi co-opts MEK as a resistance mechanism in *KRAS*-mutant ovarian, lung and colon cancer [53], further underscoring the similarity between MPM and *RAS*-driven cancer. Together, our data and the literature suggest that the efficacy of MEKi/PARPi combination therapy is closely related to RAS-MAPK activity, regardless of tumor type.

We show, for the first time, that MEKi/PARPi act synergistically in MPM through coordinate suppression of ROS homeostasis, whereby PARP1-AMPK-regulated mitochondrial SRC plays a critical role in response to MEKi-induced metabolic stress. This mechanistic finding in MPM is consistent with the growing body of evidence that oncogenic RAS/MAPK signaling rewires metabolic pathways to meet the energetic and biosynthetic demands of cancer cells and that ROS is an important metabolic manifestation of KRAS-mediated tumorigenicity [34] and asbestos carcinogenesis in MPM [13–15]. Because excess ROS is harmful, cancer cells need to maintain ROS levels in the range that promotes tumor growth but prevents cell death. Indeed, KRAS-driven cancer has evolved autophagy as an interception mechanism to keep ROS in check [54, 55]. Most importantly, we and others have recently shown that autophagy plays a critical role in asbestos-mediated transformation and apoptosis evasion in MPM [35, 36]. Here, we extend these previous findings by uncovering that RAS/MAPK and PARP1 exert a previously unrecognized function in maintaining ROS homeostasis and preventing ROS-mediated apoptosis in MPM. This is consistent with recent observations that oncogenic MAPK signaling, together with autophagy, regulates ROS in RAS/RAF-driven lung and pancreatic carcinomas [56, 57]. Interestingly, our results link the role of MEK and PARP1 in ROS surveillance to TIGAR and DJ-1, which are known to control mitochondrial ROS and oxidative stress through interaction with NRF2 [58, 59]. These findings warrant future studies to elucidate the functional interplay between RAS/MAPK signaling, the NRF2/TIGAR/DJ-1 network, PARP1, and autophagy in MPM.

Overall, our studies demonstrate that oncogenic activation of RAS/MAPK signaling is a key feature of MPM, independent of tumor heterogeneity, and that MAPK signaling and PARP1 homeostatically regulate ROS and promote evasion of ROS-mediated apoptosis, providing a novel therapeutic rationale for the treatment of the disease.

This study has some limitations. For example, some correlative analyses lack experimental validation and the mechanistic studies relied on only two MPM cell lines. The relationship between MEKi and HR defect was not explored in detail in the MPM model, although this issue is not the focus of the current study and there is sufficient evidence for this relationship in other cancers [47, 53].

## Materials And Methods

### Cell culture and reagents

MPM cell lines H28 (NCI-H28), H2452 (NCI-H2452), H2052 (NCI-H2052) and lung fibroblasts hFb16Lu (CCD-16Lu) were obtained from ATCC (American Type Culture Collection, Manassas, VA, USA), MESO-1 (ACC-MESO-1) from RIKEN Cell Bank (Ibaraki, Japan), MSTO-211H and JL-1 cells from German Collection of Microorganisms and Cell Cultures (Brunswick, Germany). The primary BE261T cells derived from surgically resected MPM tumors of a 67-year-old male were described previously [5, 36, 40]. Cells were authenticated by DNA fingerprinting and cultured in RPMI-1640 (Sigma-Aldrich) plus 10% FBS (Life Technologies) and 1% penicillin/streptomycin (Sigma-Aldrich) at 37°C in a humid incubator with 5% CO_2_. Cell lines and chemical inhibitors are listed in Table S1, S2.

### Cell viability assay, drug synergy analysis and clonogenic assay

Cells seeded in 96-well plates (1000–1500 cells/well) were treated with drugs 24 h later and assayed by Acid Phosphatase Assay Kit (ab83367; Abcam). Drug synergy was determined by CompuSyn software (www.combosyn.com) to calculate combination index (CI) and fraction affected (Fa), with CI < 1.0 indicating synergy, as previously described [60, 61]. For the clonogenic assay, cells seeded (10^3^–10^4^ cells/well) at 6-well plates were treated for 72 h and cultured in drug-free medium for additional 2–3 weeks depending on growth rates. Surviving cells were fixed by methanol (1%) and formaldehyde (1%) and visualized by crystal violet (0.5% in 25% methanol).

### Immunoblotting and immunohistochemistry (IHC)

Protein lysates prepared in RIPA buffer (Cell Signaling Technology) containing protease and phosphatase inhibitors (Santa Cruz) were separated by SDS-PAGE (4561033; Bio-Rad), and blotted with primary antibodies (Table S3) and anti-rabbit (926–32211) or anti-mouse (926–68020) secondary antibodies (Promega). Protein visualization and quantification were performed by Odyssey Infrared Imaging System (Li-COR Biosciences) and Image Studio Lite Ver 5.2. For IHC, formalin-fixed, paraffin-embedded (FFPE) tissues were stained with hematoxylin/eosin and p-H2AX (9718; CST) and cleaved caspase 3 (9664; CST) antibodies and visualized by the Bond Polymer Refine Detection kit (Leica Biosystems).

Whole slide images were acquired using PANNORAMIC® whole slide scanners and processed using CaseViewer (3DHISTECH Ltd.). The staining intensities of cancer and stromal cells in the images of complete tissue sections (from randomly selected four tumors in each treatment group) were automatically analyzed and quantified using QuPath software (version 0.2.0) by extracting the DAB channel intensity of p-H2AX (Nucleus OD value) and cleaved caspase 3 (Cytoplasm OD value) for each section [62–64]. For the classification of cancer and stromal cells, multiple training regions representing typical morphologies of cancer and stromal cells are first annotated. On this basis, the unique parameters for each cell type were established, which were then applied to the whole slide images.

### Gene silencing and ectopic overexpression

Cells grown at 50–70% confluence were transfected with Lipofectamine™ 2000 (11668027; Invitrogen™) according to the manufacturer’s protocol. *MAP2K1* and *MAP2K2* were knocked down by specific pooled siRNA duplexes (SR321446 and SR321447; OriGene Technologies), with a control siRNA Duplex used as negative control.

For MEK overexpression, MPM cells seeded in 6-well plates were cultured to 50–70% confluence before transfection with MEK1-GFP (Addgene #14746) and GFP control (Addgene #60360]) plasmids using Lipofectamine™ LTX Reagent with PLUS™ Reagent (Invitrogen™, #15338100) according to the manufacturer’s instructions.

### Flow cytometry (FC)-based apoptosis, cell cycle, and γ-H2AX quantification

MPM cells were treated for 72 h with vehicle, MEKi, PARPi, or in combination. After treatment, cells were harvested, suspended in binding buffer, and stained with the Annexin V Apoptosis Detection Kit-FITC (Cat. #88-8005; Thermo Fisher Scientific, Waltham, MA, USA) according to the manufacturer’s instructions.

For the combined cell cycle and gH2AX (γ-H2AX) assay, 1–2 million cells were used for each treatment group, and cells treated with 1 mM H_2_O_2_ (37 °C, 1 h) served as positive control. In brief, after drug treatment, cells were washed with phosphate-buffered saline (PBS), fixed with IC fixation buffer (Thermo Fisher Scientific #00-8222-49), and permeabilized with PBS containing 0.1% Triton X 100 and 1% FBS. Prior to intracellular staining, the permeabilized cells were incubated for 5 min at RT in 200 µL of PBS containing 10% FBS and 0.25% Fc Receptor Binding Inhibitor Functional Grade Monoclonal Antibody (14-9161-73) and washed in 1% FBS containing PBS. Intracellular staining with PerCP-eFluor 710-conjugated anti-p-H2AX (Ser139) was performed in the dark at RT for 1–2 hours or overnight at 4 °C on a rotating wheel (3 rpm). Cells were then washed twice with 2% FBS containing PBS and resuspended in the same buffer containing 0.5 µg/ml DAPI.

For cell cycle analysis only, a protocol available at https://flowcytometry-embl.de/wp-content/uploads/2016/12/DAPI-staining-.pdf was used. Fluorescence intensity was measured on an LSRII upgraded flow cytometer (BD Bioscience) and analyzed using FlowJo V10.6.2 (Tree Star, Inc. (Ashland, OR, USA).

### Intracellular ROS quantification

Intracellular ROS was measured using an OxiSelect™ Intracellular ROS Assay Kit (Catalog No. STA-342; CELL BIOLABS, INC.). Cells were seeded in triplicate at 1500–2000 cells/well (96-well plates) and drugged 24 h later with various inhibitors or vehicle control for 72 h before being subjected to ROS measurement. H_2_O_2_ treatment was used as a positive control.

### High-resolution respirometry by OROBOROS

2.1 × 10^6^ cells (10^6^/ml) treated with the indicated drugs were subjected to mitochondrial function analysis using a high-resolution Respirometry instrument (O2k-Core: Oxygraph-2k; Catalog No. 10000-02) as we previously reported [65].

### Analysis of dataset (TCGA, TCPA, DepMap, and GEO), GSEA, gene ontology terms and ROS-responsive gene signature

Level 3 and/or 4 transcriptomic and reverse-phase protein array (RPPA) data of cancer patients were obtained from The Cancer Genome Atlas (TCGA) cBioPortal (https://www.cbioportal.org/) and The Cancer Proteome Atlas (TCPA) [38], respectively. Specifically, while the level 3 data are normalized data, level 4 data are those with batch effects removed and therefore particularly useful for comparison across different cancer types (detailed instructions: https://tcpaportal.org/tcpa/faq.html). Normalization of RPPA data was processed as follows: 1) calculate the median of each protein across all samples; 2) subtract the median (from step 1) from values of each protein in all samples; 3) calculate the median of all proteins in each sample; 4) subtract the median (from step 3) from values of each sample.

R packages “limma” and “edgeR” were used to normalize transcriptomic data and identify differentially expressed genes, respectively. Gene-level transcription estimates were shown in log2(x + 1) transformed RSEM (RNA-Seq by Expectation-Maximization) normalized count. The gene expression and corresponding survival data were extracted for correlation and prognostic analysis using the corresponding packages in R (version 3.6.0) (´corrplot´ and ´Hmisc´ packages for correlation analysis; ‘maxstat’, ‘survival’ and ‘survminer’ packages for prognostic analysis). Analysis of the enriched biological pathway, process and molecular function from Gene Ontology, as well as gene interaction map, were performed using ClusterProfiler package in R [60, 63, 66–68]. The data of cancer genomic vulnerabilities were obtained from the Cancer Dependency Map Project [69]. Transcriptomic data of MPM samples (GSE12345; GSE51024; GSE2549; GSE21750) were downloaded from the Gene Expression Omnibus (GEO) database [70]. Gene set enrichment analysis (GSEA) was performed using GSEA and R software [71].

### Gene signatures

HR signatures were derived (or curated) from transcriptomic data from previous studies [45, 46]. In both papers, the BRACness signature was generated using the differential whole-genome gene expression data between breast tumors with deficient and wildtype BRCA1/2. The sum of these genes was then used to calculate HR score for each tumor type in TCGA pan-cancer cohort after scaling with the Apply function of the R package. Likewise, a ROS-responsive gene signature was generated based on transcriptomic data from H_2_O_2_-treated cancer cells (GSE32335). Clustered genes [determined by log_2_ FoldChage (FC) > 2, adjusted.p-value < 0.05] were included to define the ROS responsive gene signature.

### Animal studies

Mouse studies were conducted in age- and gender-matched NSG (NOD-*SCID IL2Rγ*^*null*^) in accordance with Institutional Animal Care and Ethical Committee-approved animal guidelines and protocols. Tumor cells (3 × 10^6^) mixed with Matrigel (Cat. # 356231; Corning) were subcutaneously inoculated in the left and right flanks to establish xenografts as we previously described [36, 40, 61]. Treatment was initiated when tumors reached 100-200 mm^3^, with trametinib (P.O.; daily) and olaparib (i.p. daily) dissolved in PBS containing 4% DMSO and 10% 2-hydroxy-propyl-β-cyclodextrin and administered sequentially. Tumor volume was calculated by the formula: (D x d^2^)/2, where “D” refers to the long tumor diameter and “d” refers to the short tumor diameter.

### Statistical analysis

Statistical analyses were performed using one-way/two-way analysis of variance (ANOVA), Bonferroni’s multiple comparison test, and Student’s *t*-test using GraphPad Prism 7 unless otherwise indicated. Data were shown as mean ± s.d. of at least three biological replicates (n). Sample size was not pre-determined by statistical methods but rather based on preliminary experiments. Group allocation was performed randomly, with all samples that met proper experimental conditions included in the analysis. The correlation coefficient (Spearman) was determined by using R (version 3.6.0). For survival analysis, patients were stratified by ‘high’ and ‘low’ expression, which is determined by the optimal cutoff value. *P* < 0.05 was considered statistically significant.

## Supplementary information

Supplemental Material

Original Data File

Authorship change agreement

## Data Availability

All data generated in this study are included in this article and the supplementary material.
