# Supplementary material for: MEK1 drives oncogenic signaling and interacts with PARP1 for genomic and metabolic homeostasis in malignant pleural mesothelioma
Source: Cell Death Discov. 2023 Feb 10;9:55. doi: 10.1038/s41420-023-01307-2 (PMC9918536; doi:10.1038/s41420-023-01307-2)
Supplement: Supplementary file 1 — Supplemental Material [file 41420_2023_1307_MOESM1_ESM.pdf]

Supplementary Material

MEK1 drives oncogenic signaling and interacts with PARP1 for genomic and metabolic homeostasis in malignant pleural mesothelioma

Haitang Yang[1,2,3†] Yanyun Gao[1,2†], Duo Xu[1,2], Ke Xu[3], Shun-Qing Liang[1,2¶], Zhang Yang[1,2], Amina Scherz[4], Sean R.R. Hall[1,2], Stefan Forster[2,4,] Sabina Berezowska[5], Feng Yao[3], Adrian F. Ochsenbein[2,4], Thomas M. Marti[1,2], Gregor J. Kocher[1,2], Ralph A. Schmid[1,2*], Patrick Dorn[1,2*], Ren-Wang Peng[1,2*]

This PDF files include:

Supplementary Figures S1-S6

Supplementary Tables:

Table S1. Cell lines used in this study

Table S2. Compounds used in this study

Table S3. Antibodies used in this study

Table S4. p-MEK1 (Ser217/221) correlated proteins in MPM

Table S5. ROS responsive gene signature

Fig. S1

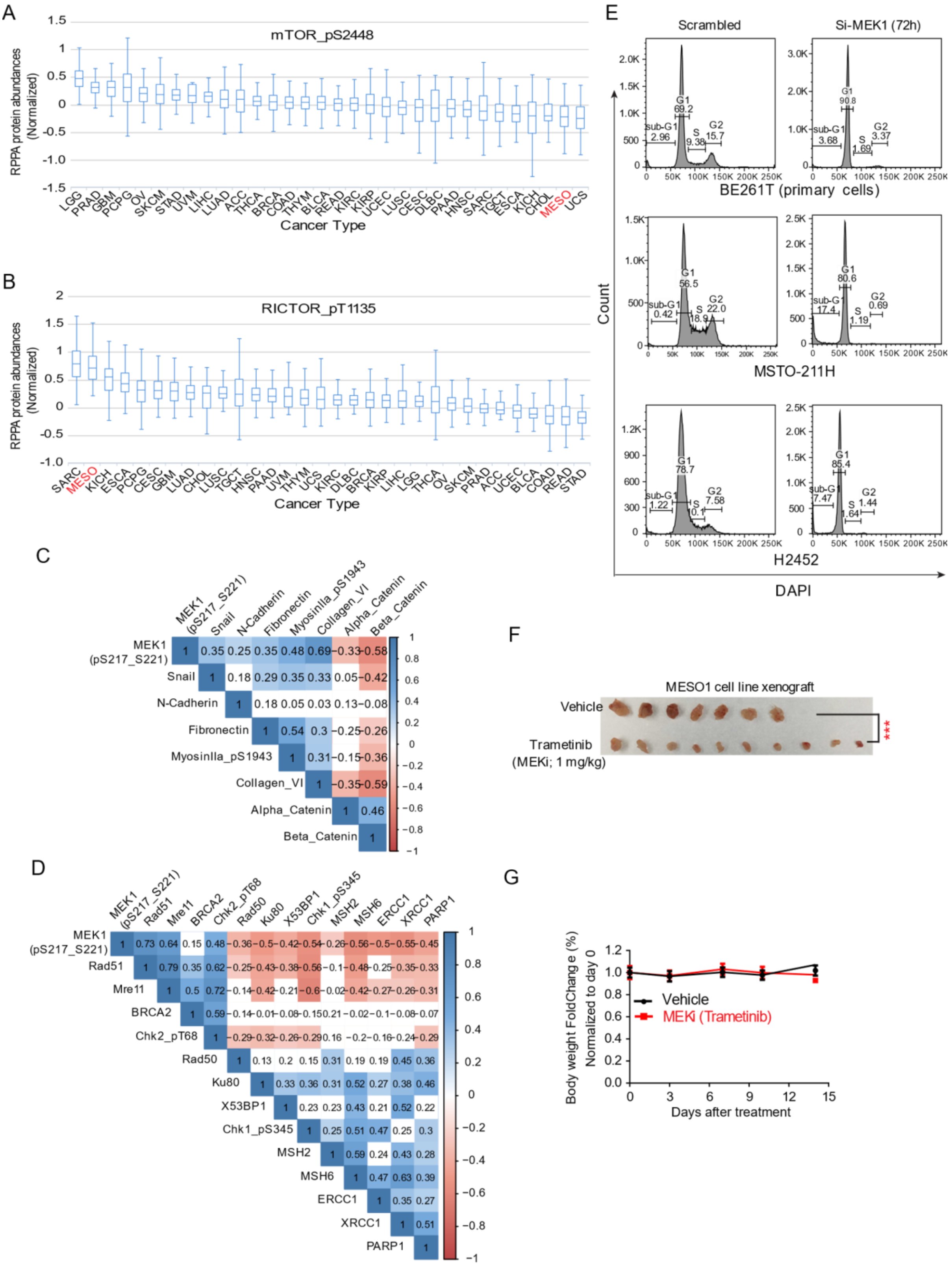
A
mTOR_pS2448
RPPA protein abundances (Normalized)
Cancer Type
B
RICTOR_pT1135
RPPA protein abundances (Normalized)
Cancer Type
C
D
E
Scrambled
Si-MEK1 (72h)
BE261T (primary cells)
MSTO-211H
H2452
Count
DAPI
F
MESO1 cell line xenograft
Vehicle
Trametinib (MEKi; 1 mg/kg)
G
Body weight FoldChange (%) Normalized to day 0
Vehicle
MEKi (Trametinib)
Days after treatment

Fig. S1. MEK is hyperactive and a molecular driver in MPM.

A, B, p-mTOR (Ser2448) and p-RICTOR (Thr1135) protein levels in patient tumors. Proteomic data are downloaded from the Cancer Proteome Atlas (TCPA) database.

C-D, p-MEK1 (Ser217/221) protein level in patient MPM tumors significantly correlates with cancer-related proteins involved in epithelial-to-mesenchymal transition (C) and DNA damage repair (D). The numbers in the correlogram indicate the correlation coefficient (Spearman). Significant positive (in blue) and negative (in red) correlations are shown, with the color intensity proportional to the correlation coefficient. Non-significant correlation is in blank background. Proteomic data of patient MPM tumors (n=61) are downloaded from the TCPA database.

E, Flow cytometry (FC)-based cell-cycle analyses of MPM cells after 72h transfection with MEK1-targeting siRNAs (si-MEK1).

F-G, Tumor size (F) and mouse body weight (G) of MESO1 xenografts treated with trametinib (MEKi; 0125 mg/kg) for the indicated time. ***p<0.001 by Welch´s test.

Fig. S2

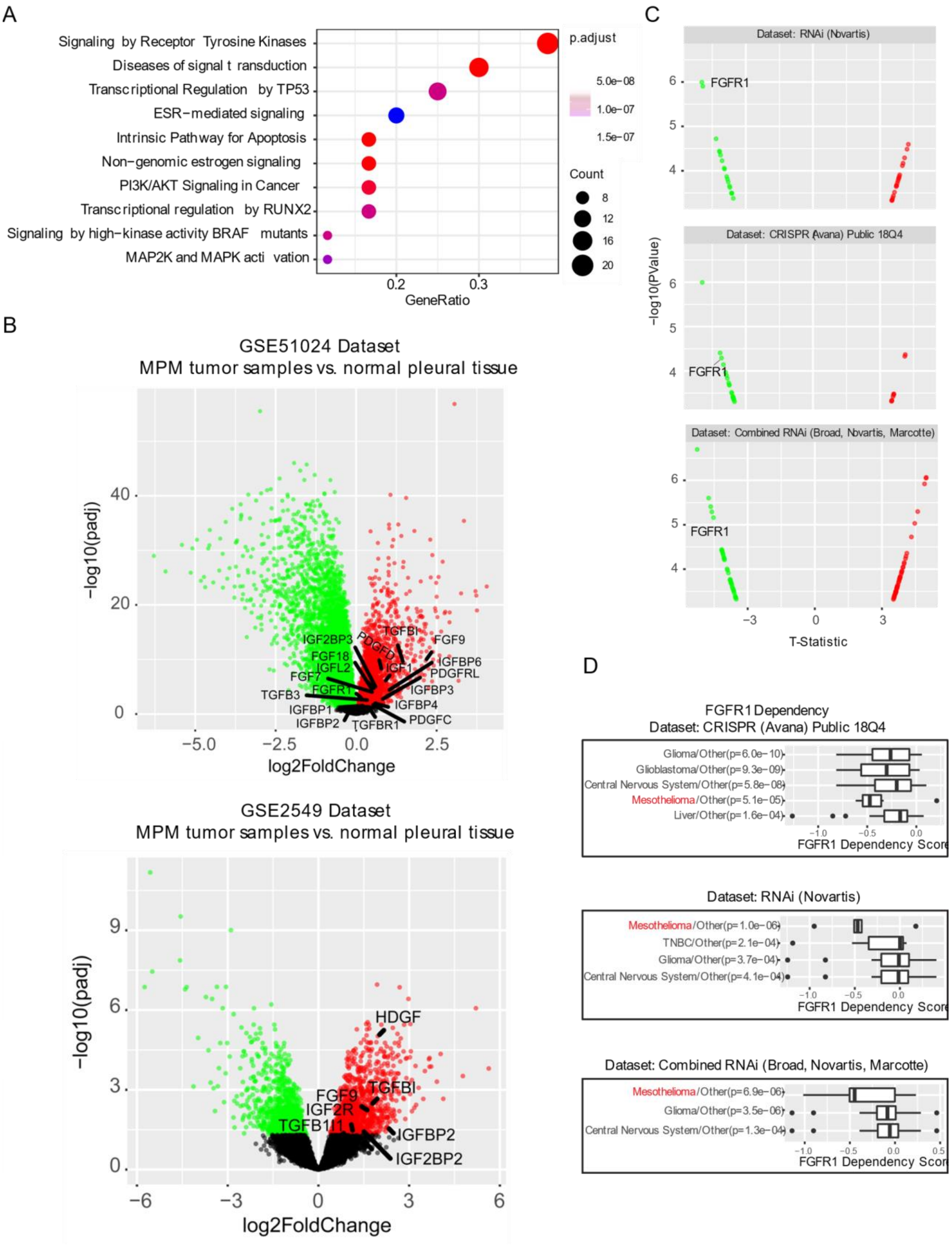
A
Signaling by Receptor Tyrosine Kinases
Diseases of signal t ransduction
Transcriptional Regulation by TP53
ESR−mediated signaling
Intrinsic Pathway for Apoptosis
Non−genomic estrogen signaling
PI3K/AKT Signaling in Cancer
Transcriptional regulation by RUNX2
Signaling by high−kinase activity BRAF mutants
MAP2K and MAPK acti vation
GeneRatio
p.adjust
5.0e−08
1.0e−07
1.5e−07
Count
8
12
16
20
B
GSE51024 Dataset
MPM tumor samples vs. normal pleural tissue
−log10(padj)
log2FoldChange
IGF2BP3
PDGFD
TGFBI
FGF9
FGF18
IGFL2
FGF7
IGF1
IGFBP6
PDGFRL
TGFB3
FGFR1
IGFBP3
IGFBP1
IGFBP4
IGFBP2
TGFBR1
PDGFC
GSE2549 Dataset
MPM tumor samples vs. normal pleural tissue
−log10(padj)
log2FoldChange
HDGF
FGF9
TGFBI
IGF2R
TGFB1I1
IGFBP2
IGF2BP2
C
Dataset: RNAi (Novartis)
FGFR1
Dataset: CRISPR (Avana) Public 18Q4
FGFR1
Dataset: Combined RNAi (Broad, Novartis, Marcotte)
FGFR1
−log10(PValue)
T-Statistic
D
FGFR1 Dependency
Dataset: CRISPR (Avana) Public 18Q4
Glioma/Other(p=6.0e−10)
Glioblastoma/Other(p=9.3e−09)
Central Nervous System/Other(p=5.8e−08)
Mesothelioma/Other(p=5.1e−05)
Liver/Other(p=1.6e−04)
FGFR1 Dependency Score
Dataset: RNAi (Novartis)
Mesothelioma/Other(p=1.0e−06)
TNBC/Other(p=2.1e−04)
Glioma/Other(p=3.7e−04)
Central Nervous System/Other(p=4.1e−04)
FGFR1 Dependency Score
Dataset: Combined RNAi (Broad, Novartis, Marcotte)
Mesothelioma/Other(p=6.9e−06)
Glioma/Other(p=3.5e−06)
Central Nervous System/Other(p=1.3e−04)
FGFR1 Dependency Score

Fig. S2. Functional dependence of MPM cells on RTK signaling.

A, Enriched signaling pathways that significantly (p-value < 0.05) positively correlate with p-MEK1 (S217/S221) protein level in patient MPM tumors. Pathway enrichment analysis is based on proteomic data of a cohort of MPM patients (n=61) in TCPA.

B, Transcriptomic profiling of MPM specimens versus normal pleural tissues. Data analysis is based on GSE51024 and GSE2549. The X-axis is the fold-change (log2) of individual genes in tumors compared to matched normal pleura. The genes significantly downregulated [adjusted p-value (padj) < 0.05)] and upregulated in the tumor are marked in green and red, respectively. Note that multiple RTKs and their ligands are significantly upregulated in MPM.

C, **D**, FGFR1 is an oncogenic dependency of MPM. The negative score indicates reduced cell proliferation of MPM cells (n=18) upon genetic inhibition of FGFR1 (C). FGFR1 dependency is based on genome-wide CRISPR and RNA interference of the Cancer Dependency Map Project, with MPM being one of the top enriched lineages (p-values < 0.05) of the pan-cancer cells (D).

Fig. S3

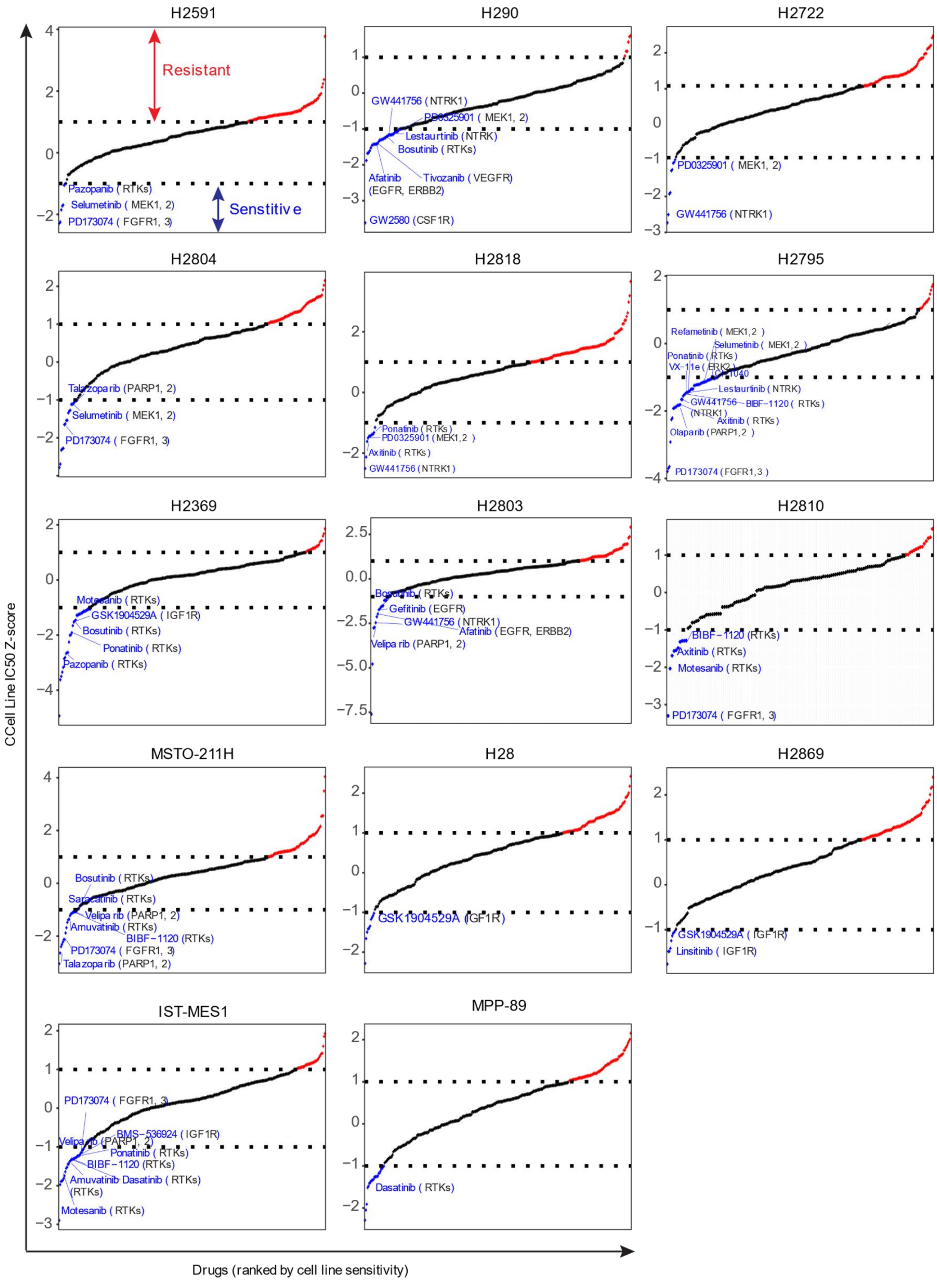
H2591
Resistant
Sensitive
Pazopanib (RTKs)
Selumetinib (MEK1, 2)
PD173074 (FGFR1, 3)
H290
GW441756 (NTRK1)
PD0325901 (MEK1, 2)
Lestaurtinib (NTRK)
Bosutinib (RTKs)
Afatinib (EGFR, ERBB2)
Tivozanib (VEGFR)
GW2580 (CSF1R)
H2722
PD0325901 (MEK1, 2)
GW441756 (NTRK1)
H2804
Talazoparib (PARP1, 2)
Selumetinib (MEK1, 2)
PD173074 (FGFR1, 3)
H2818
Ponatinib (RTKs)
PD0325901 (MEK1,2)
Axitinib (RTKs)
GW441756 (NTRK1)
H2795
Refametinib (MEK1,2)
Selumetinib (MEK1,2)
Ponatinib (RTKs)
VX-11e (ERK2)
Lestaurtinib (NTRK)
GW441756 (NTRK1)
BIBF-1120 (RTKs)
Axitinib (RTKs)
Olaparib (PARP1,2)
PD173074 (FGFR1,3)
H2369
Motesanib (RTKs)
GSK1904529A (IGF1R)
Bosutinib (RTKs)
Ponatinib (RTKs)
Pazopanib (RTKs)
H2803
Bosutinib (RTKs)
Gefitinib (EGFR)
GW441756 (NTRK1)
Afatinib (EGFR, ERBB2)
Veliparib (PARP1, 2)
H2810
BIBF-1120 (RTKs)
Axitinib (RTKs)
Motesanib (RTKs)
PD173074 (FGFR1, 3)
MSTO-211H
Bosutinib (RTKs)
Saracatinib (RTKs)
Veliparib (PARP1, 2)
Amuvatinib (RTKs)
BIBF-1120 (RTKs)
PD173074 (FGFR1, 3)
Talazoparib (PARP1, 2)
H28
GSK1904529A (IGF1R)
H2869
GSK1904529A (IGF1R)
Linsitinib (IGF1R)
IST-MES1
PD173074 (FGFR1, 3)
BMS-536924 (IGF1R)
Veliparib (PARP1, 2)
Ponatinib (RTKs)
BIBF-1120 (RTKs)
Amuvatinib (RTKs)
Dasatinib (RTKs)
Motesanib (RTKs)
MPP-89
Dasatinib (RTKs)
CCell Line IC50 Z-score
Drugs (ranked by cell line sensitivity)

Fig. S3. MPM cells are highly sensitive to inhibitors of RTK signaling.

Drug sensitivity profiling of MPM cell lines (n=14). Each dot represents a specific inhibitor, with the red dot indicating resistance (high IC_{50} Z-score) of the indicated MPM cells to an inhibitor and the blue dot indicating sensitivity (low IC_{50} Z-score) to an inhibitor. Inhibitors targeting receptor tyrosine kinases (RTKs) are highlighted. Data were downloaded from Genomics of Drug Sensitivity in Cancer (GDSC).

Fig. S4

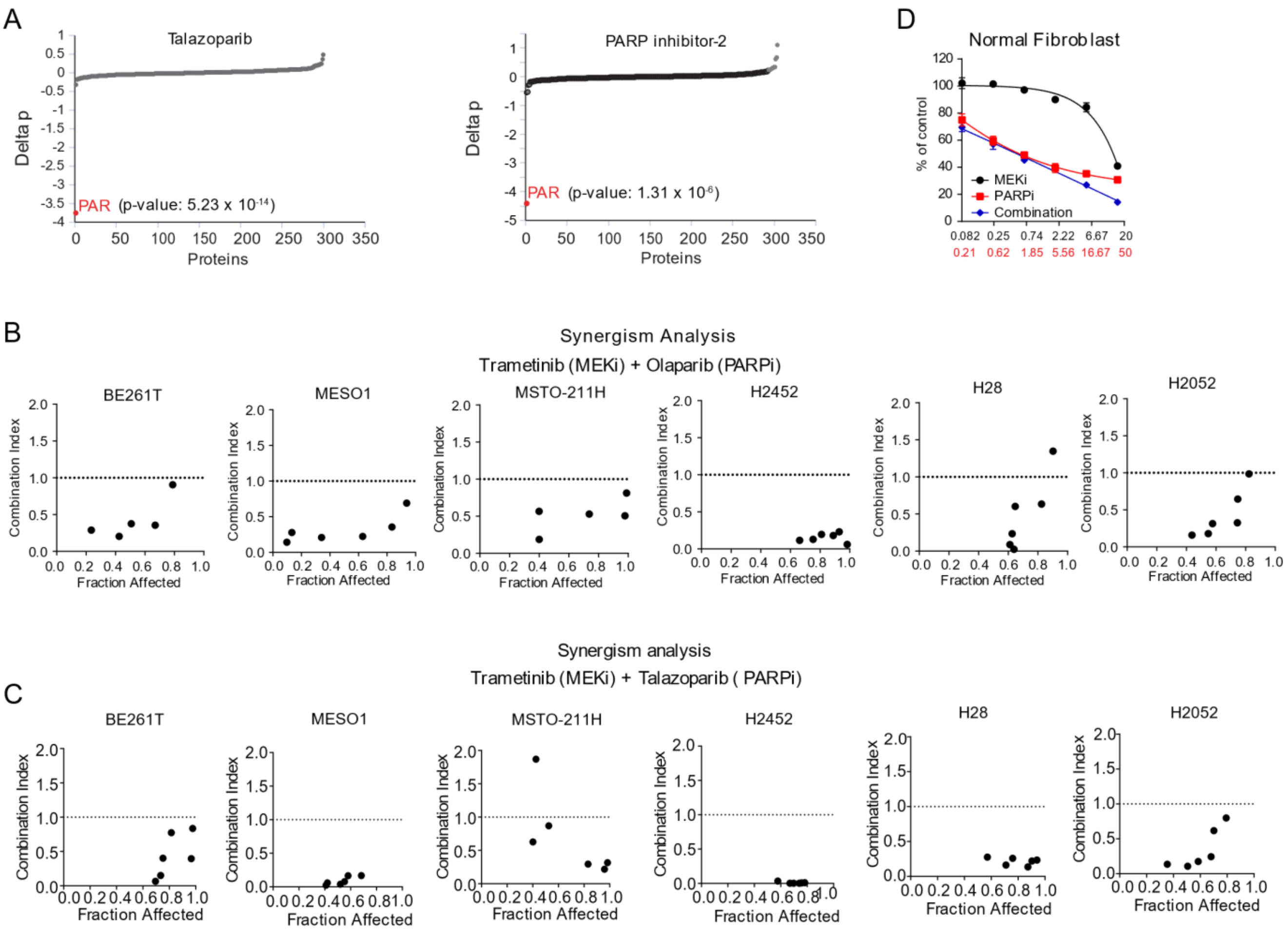

Fig. S4. Synergistic effects of MEKi and PARPi in MPM cells.

A, PAR (product of PARP) levels are most significantly correlated with the sensitivity to PARP inhibitors (Talazoparib; PARP inhibitor-2) in cancer cells. Delta p: pairwise correlation of protein response. Drug sensitivity data of pan-cancer cell lines were downloaded from the Cancer Proteomics Atlas (https://tcpaportal.org/cppa/#/analysis).

B, C, Synergy analysis of trametinib (MEKi) with olaparib (PARPi) (B), and trametinib (MEKi) with talazoparib (PARPi) (C). Fraction affected (Fa) and combination index (CI) are determined by the CompuSyn software, with CI < 1 indicating drug synergy.

D, Dose-response curves of normal fibroblast cells treated with trametinib and talazoparib (PARPi). Data are shown as mean ± s.d. (n=3).

Fig. S5

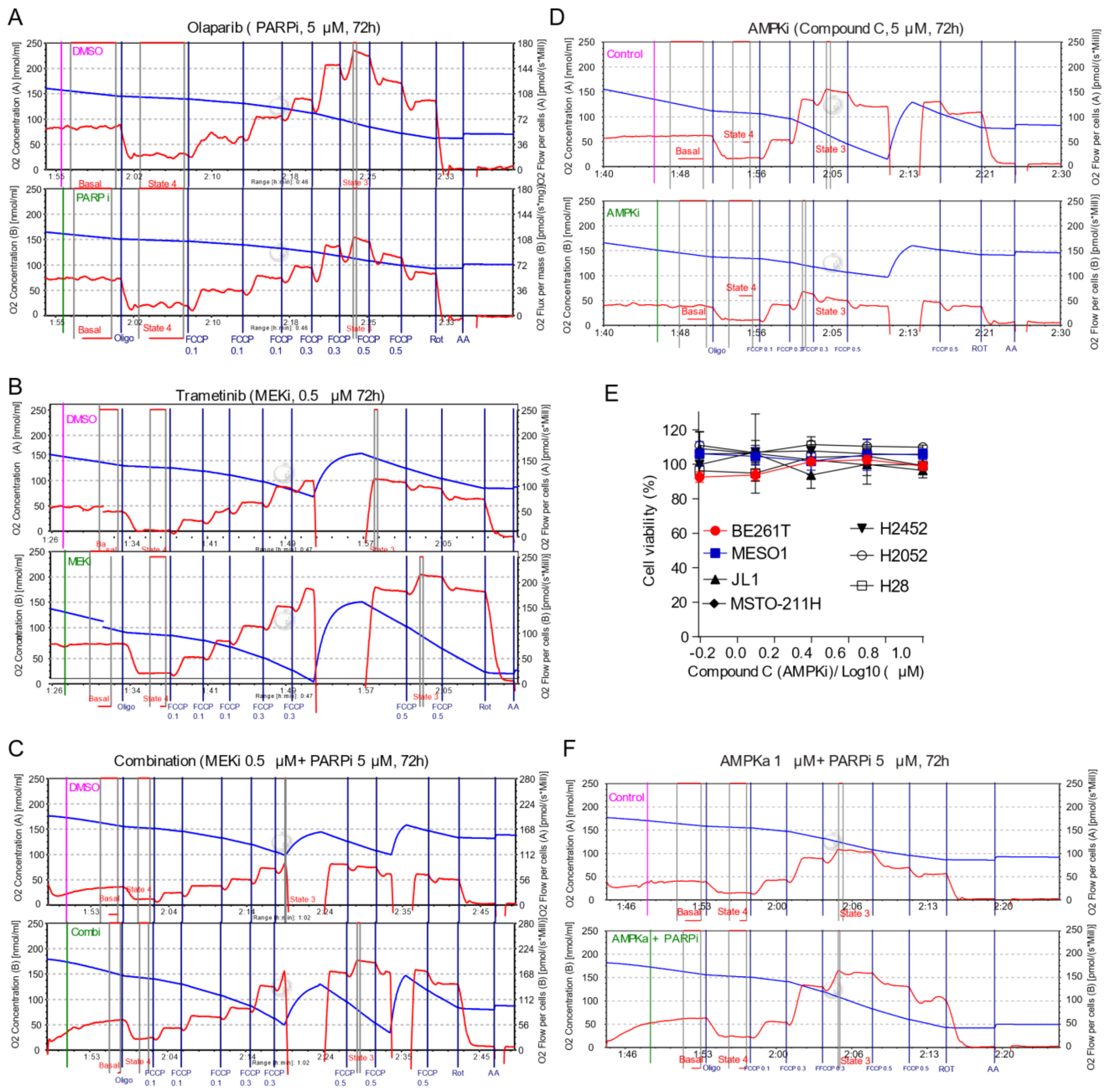

Fig. S5. PARP and MEK cooperate to regulate mitochondrial functions in MPM.

A-C, High-resolution respirometry (OROBOROS) shows real-time oxygen consumption rate (OCR) of MESO-1 cells treated with DMSO, PARPi (olaparib), MEKi (trametinib), and the combination, with horizontal axis indicating time (hour: min), the left vertical axis indicating oxygen (O_2) consumption rate (red line) and the right vertical axis indicating O_2 concentration in the chamber (blue line). Oligo, oligomycin, an inhibitor of complex V (ATP synthase); FCCP, mitochondrial oxidative phosphorylation uncoupler, with FCCP 0.1, FCCP 0.3, and FCCP 0.5 indicating 0.1 μM FCCP, 0.3 μM, and 0.5 μM at final concentration, respectively;

Rot, Rotenone, complex I inhibitor; AA, Antimycin A, complex III inhibitor. Shown in the upper panel are MESO-1 cells treated with DMSO and the lower panel are MESO-1 cells treated with the indicated drugs.

D, Real-time OCR of MESO1 cells treated for 72h with DMSO, AMPK inhibitor (AMPKi; 5 µM compound C).

E, Dose-response curves of the indicated MPM cells treated with different doses of compound C (AMPKi). Data are shown as mean ± s.d. of three biological replicates (n=3).

F, Real-time OCR of MESO1 cells treated for 72h with DMSO, AMPK activator (AMPKa; 1 mM) in combination with PARPi (5 µM olaparib).

Fig. S6

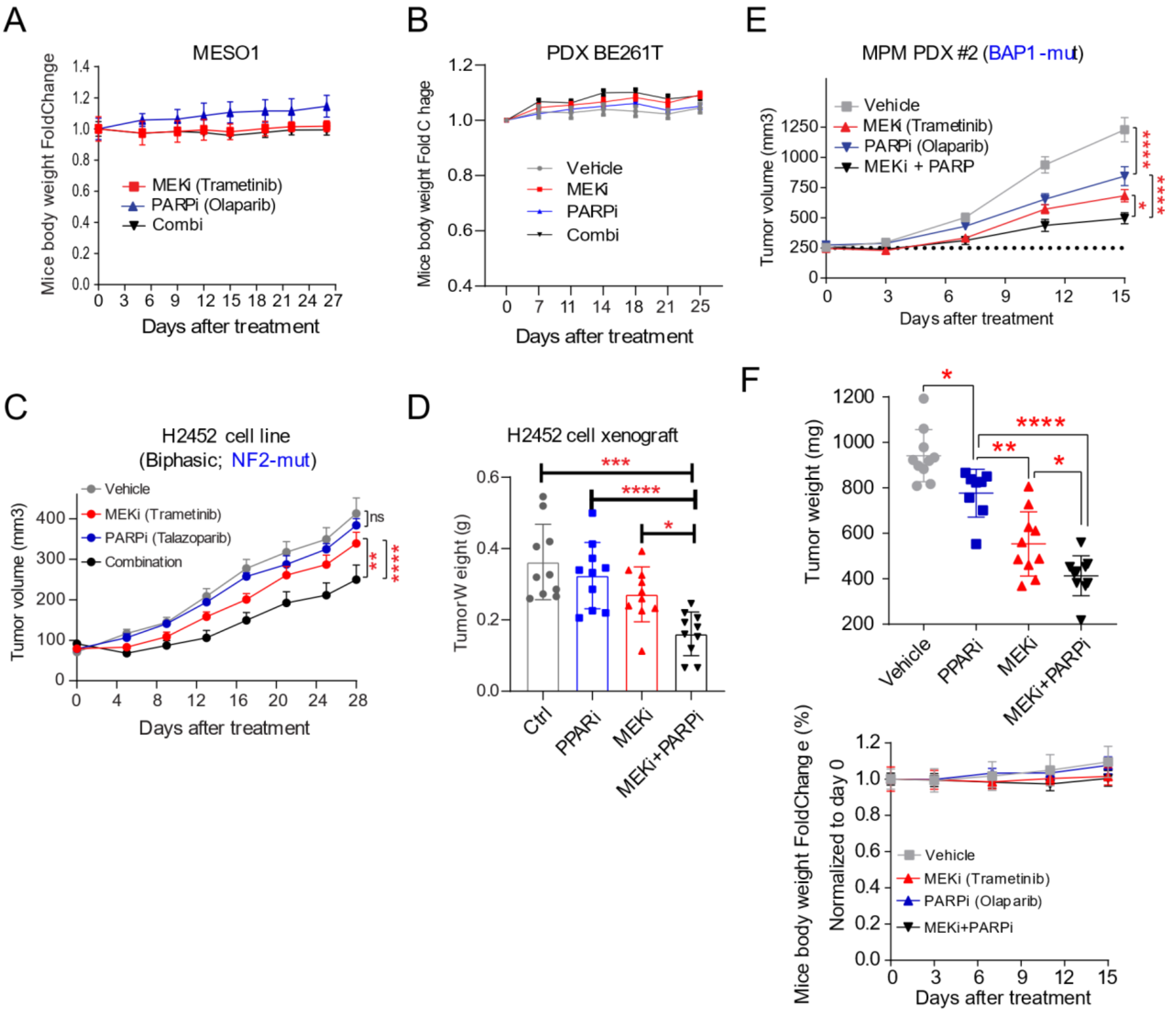

Fig. S6. *In vivo* efficacy of MEKi/PARPi.

A, B, Mice body weights of MESO1 xenograft (A) and PDX BE261T (B) models during the treatment. Data were normalized to the body weight of day 0 and presented as mean ± SEM. **C, D,** Tumor development during the treatment (C) and tumor weight (D) at the end of the treatment in H2452 (*NF2*-mutant) xenografts. **E, F,** Tumor development (E), tumor weight and mouse body weight (F) of a patient-derived xenograft (PDX) model (*BAP1*-mutant). Data are shown as mean ± SEM. *p<0.05, ****p<0.0001 by two-way ANOVA.

Table S1. Cell lines used in this study

**Cell**	**Histology subtype**	***CDKN2A/2B***	***BAP1***	***NF2***	***TP53***
BE261T (Primary)	Biphasic	Homozygous deletion	Homozygous deletion	unknown	unknown
MESO1	Epithelioid	Homozygous deletion	Wild-type	Nonsense_Mutation;p.Gln389Ter (c.1165C>T)	Wild-type
JL1	Epithelioid	Homozygous deletion	Homozygous deletion	Homozygousdeletion	Wild-type
H2452	Biphasic	Homozygous deletion	Homozygous for BAP1 p.Ala95Asp (c.284C>A)/Missense	Wild-type	Wild-type
MSTO-211H	Biphasic	Homozygous deletion	Wild-type	Wild-type	Wild-type
H28	Sarcomatoid	Homozygous deletion	Homozygous for BAP1 p.Arg146Alafs*48; Splice_Site	Wild-type	Wild-type
H2052	Sarcomatoid	Homozygous deletion	Wild-type	Wild-type	Wild-type
hFb16Lu	Fibroblast	Wild-type	Wild-type	Wild-type	Wild-type

Table S2. Compounds used in this study

Inhibitor	Target	Developmental Stage	Company	Catalog No.
Trametinib	MEK1/2	Approved	ChemScene	CS-0060
Olaparib	PARP1/2	Approved	ChemScene	CS-0075
Talazoparib	PARP1/2	Approved	ChemScene	CS-0937
Sorafenib	C-RAF (RAF1), B-RAF, VEGFR-2	Approved	ChemScene	CS-1590
Linsitinib	IGF-1R	Approved	ChemScene	CS-0242
Dovitinib	Multiple RTKs	Approved	ChemScene	CS-6230
Ponatinib	Multiple RTKs	Approved	ChemScene	CS-0204
Compound C	AMPK		ChemScene	CS-2487
Necrostatin-1	Necroptosis		Selleckchem	S8037
Fer-1	Ferroptosis		Selleckchem	S7243
Q-VD-Oph	Pan-caspase		Selleckchem	S7311
N-acetylcysteineamide (NAC)	ROS scavenger		Selleckchem	S5804
Hydroxychloroquine(HCQ)	Autophagy	Approved	Selleckchem	S4430
**Activator**	**Target**	**Developmental Stage**	**Company**	**Catalog No.**
AICAR	AMPK	Phase 3	ChemScene	CS-1951

Table S3. Antibodies used in this study

Antibody	Source	Catalog No.
Monoclonal p44/42 MAPK (Erk1/2) Antibody	Cell Signaling Technology	4695T
Monoclonal Phospho-p44/42 MAPK (Erk1/2) (Thr202/Tyr204)	Cell Signaling Technology	4377S
Monoclonal anti-E-Cadherin (4A2) Antibody	Cell Signaling Technology	14472S
Monoclonal anti-Vimentin (D21H3) Antibody	Cell Signaling Technology	5741
Monoclonal anti-MEK1/2 Antibody	Cell Signaling Technology	9122S
Monoclonal Phospho-MEK1/2 (Ser217/221) Antibody	Cell Signaling Technology	9154T
Monoclonal anti-Cleaved Caspase-7 (Asp198) Antibody	Cell Signaling Technology	8438P
Monoclonal anti-beta-Actin (8H10D10) Antibody	Cell Signaling Technology	3700S
Monoclonal anti-Bcl-2 Antibody	Cell Signaling Technology	15071
Monoclonal anti-Bcl-xL Antibody	Cell Signaling Technology	2764
Monoclonal anti-BRCA1 antibody	Cell Signaling Technology	9010
Monoclonal anti-MRE11 antibody	Cell Signaling Technology	4847T
Monoclonal anti-Phospho-Histone H2A.X (Ser139) antibody	Cell Signaling Technology	9718
anti-Poly/Mono-ADP Ribose antibody	Cell Signaling Technology	83732
Monoclonal anti-AMPKα Antibody	Cell Signaling Technology	2793
Monoclonal anti-Phospho-AMPKα (Thr172) Antibody	Cell Signaling Technology	50081
Monoclonal anti-Phospho-Acetyl-CoA Carboxylase (Ser79) antibody	Cell Signaling Technology	3661S
800CW Donkey anti- IgG (H + L) Secondary Antibody	LI-COR Biosciences	925-32213
800CW Goat anti- IgG (H + L) Secondary Antibody	LI-COR Biosciences	926-32210
Bim (C34C5) Rabbit mAb	Cell Signaling Technology	2933T

Table S4: p-MEK1 (Ser217/221) correlated proteins in MPM

**Positive correlation**			
**Protein A**	**Protein B**	**Spearman correlation coefficient**	**p-value**
MEK1_pS217_S221	Rictor_pT1135	0.784346906	7.59E-14
MEK1_pS217_S221	Stathmin	0.781491274	1.07E-13
MEK1_pS217_S221	Bid	0.745108408	5.73E-12
MEK1_pS217_S221	Rad51	0.73442623	1.63E-11
MEK1_pS217_S221	Bak	0.729772607	2.52E-11
MEK1_pS217_S221	Bcl2A1	0.702485457	2.80E-10
MEK1_pS217_S221	LKB1	0.690481227	7.41E-10
MEK1_pS217_S221	Collagen VI	0.688894765	8.39E-10
MEK1_pS217_S221	TTF1	0.684769963	1.16E-09
MEK1_pS217_S221	HSP70	0.655050238	1.02E-08
MEK1_pS217_S221	Rab11	0.641089371	2.61E-08
MEK1_pS217_S221	Mre11	0.640930724	2.64E-08
MEK1_pS217_S221	IGF1R_pY1135_Y1136	0.630248546	5.25E-08
MEK1_pS217_S221	Rb	0.599524061	3.30E-07
MEK1_pS217_S221	Chk1_pS296	0.588313062	6.16E-07
MEK1_pS217_S221	TIGAR	0.585404548	7.22E-07
MEK1_pS217_S221	TAZ	0.57678477	1.14E-06
MEK1_pS217_S221	ER-alpha	0.5755156	1.22E-06
MEK1_pS217_S221	Cyclin D1	0.572501322	1.43E-06
MEK1_pS217_S221	CD31	0.568958223	1.72E-06
MEK1_pS217_S221	X14-3-3_beta	0.561819143	2.46E-06
MEK1_pS217_S221	p90RSK_pT359_S363	0.552511898	3.90E-06
MEK1_pS217_S221	MAPK_pT202_Y204	0.552247488	3.95E-06
MEK1_pS217_S221	LCK	0.542622951	6.26E-06
MEK1_pS217_S221	C-Raf_pS338	0.527763088	1.24E-05
MEK1_pS217_S221	p27_pT198	0.522527763	1.56E-05
MEK1_pS217_S221	Caspase_8	0.513537811	2.31E-05
MEK1_pS217_S221	Heregulin	0.509095717	2.79E-05
MEK1_pS217_S221	Chk2_pT68	0.483236383	7.99E-05
MEK1_pS217_S221	Myosin IIa_pS1943	0.47831835	9.66E-05
MEK1_pS217_S221	eIF4E	0.47704918	0.000101
MEK1_pS217_S221	MEK1	0.476255949	0.000105
MEK1_pS217_S221	p38_pT180_Y182	0.4377578	0.000419
MEK1_pS217_S221	MYH11	0.42781597	0.000584
MEK1_pS217_S221	AR	0.426388154	0.000612
MEK1_pS217_S221	c-Myc	0.422897938	0.000685
MEK1_pS217_S221	c_Met_pY1235	0.412162877	0.000965
MEK1_pS217_S221	Bcl-xL	0.410312004	0.001023
MEK1_pS217_S221	PR	0.408566896	0.00108
MEK1_pS217_S221	ACVRL1	0.399629825	0.001419
MEK1_pS217_S221	p70S6K_pT389	0.398519302	0.001467

MEK1_pS217_S221	Akt_pS473	0.396879958	0.001541
MEK1_pS217_S221	ERK2	0.394764675	0.001642
MEK1_pS217_S221	CDK1	0.385351666	0.002164
MEK1_pS217_S221	YAP	0.384981491	0.002187
MEK1_pS217_S221	FOXO3a	0.376044421	0.00282
MEK1_pS217_S221	YAP_pS127	0.370967742	0.003248
MEK1_pS217_S221	DJ-1	0.363405605	0.003993
MEK1_pS217_S221	Notch1	0.363352723	0.003999
MEK1_pS217_S221	MIG-6	0.358011634	0.004613
MEK1_pS217_S221	Snail	0.35383395	0.00515
MEK1_pS217_S221	Fibronectin	0.351665785	0.00545
MEK1_pS217_S221	Transglutaminase	0.329032258	0.00962
MEK1_pS217_S221	Caveolin-1	0.326493919	0.010228
MEK1_pS217_S221	p38_MAPK	0.325859334	0.010384
MEK1_pS217_S221	INPP4B	0.323109466	0.011089
MEK1_pS217_S221	X14-3-3-zeta	0.315230037	0.013341
MEK1_pS217_S221	IGFBP2	0.30586991	0.016518
MEK1_pS217_S221	Paxillin	0.303966155	0.017238
MEK1_pS217_S221	p16_INK4a	0.302326811	0.017879
MEK1_pS217_S221	Akt_pT308	0.299312533	0.019112
MEK1_pS217_S221	Src_pY527	0.299259651	0.019134
MEK1_pS217_S221	IRS1	0.297250132	0.019996
MEK1_pS217_S221	N-RAS	0.289635114	0.023568
MEK1_pS217_S221	Bax	0.287678477	0.024569
MEK1_pS217_S221	CD49b	0.282760444	0.027243
MEK1_pS217_S221	SETD2	0.281226864	0.028126
MEK1_pS217_S221	p27_pT157	0.280116341	0.028779
MEK1_pS217_S221	PD-L1	0.267054469	0.037474
MEK1_pS217_S221	HER3	0.261872025	0.04148
MEK1_pS217_S221	JNK_pT183_pY185	0.254944474	0.04738
MEK1_pS217_S221	N-Cadherin	0.254151243	0.048098
**Negative correlation**			
**Protein A**	**Protein B**	**Spearman correlation coefficient**	**p-value**
MEK1_pS217_S221	mTOR	-0.693601269	5.78E-10
MEK1_pS217_S221	eIF4G	-0.690957166	7.13E-10
MEK1_pS217_S221	RBM15	-0.668006346	4.07E-09
MEK1_pS217_S221	B_Raf	-0.640719196	2.67E-08
MEK1_pS217_S221	S6	-0.63902697	2.99E-08
MEK1_pS217_S221	BRD4	-0.619619249	1.01E-07
MEK1_pS217_S221	ER_alpha_pS118	-0.600846113	3.06E-07
MEK1_pS217_S221	Tuberin	-0.59746166	3.71E-07
MEK1_pS217_S221	Jak2	-0.588207298	6.20E-07
MEK1_pS217_S221	beta_Catenin	-0.584135378	7.73E-07
MEK1_pS217_S221	STAT5_alpha	-0.570703332	1.57E-06
MEK1_pS217_S221	Cyclin_E2	-0.568059228	1.80E-06

MEK1_pS217_S221	MSH6	-0.563881544	2.22E-06
MEK1_pS217_S221	ACC1	-0.55790587	2.99E-06
MEK1_pS217_S221	XRCC1	-0.546377578	5.24E-06
MEK1_pS217_S221	Chk1_pS345	-0.544843998	5.63E-06
MEK1_pS217_S221	HER2	-0.54362771	5.97E-06
MEK1_pS217_S221	ACC_pS79	-0.538762559	7.49E-06
MEK1_pS217_S221	ARID1A	-0.535536753	8.70E-06
MEK1_pS217_S221	FASN	-0.523109466	1.52E-05
MEK1_pS217_S221	p62_LCK_ligand	-0.516023268	2.08E-05
MEK1_pS217_S221	ERCC1	-0.503807509	3.49E-05
MEK1_pS217_S221	Ku80	-0.499788472	4.12E-05
MEK1_pS217_S221	ATM	-0.471549445	0.000125
MEK1_pS217_S221	PARP1	-0.445214172	0.000324
MEK1_pS217_S221	PKC_delta_pS664	-0.445108408	0.000325
MEK1_pS217_S221	eEF2K	-0.44082496	0.000377
MEK1_pS217_S221	COG3	-0.440507668	0.000381
MEK1_pS217_S221	Chk2	-0.435378107	0.000454
MEK1_pS217_S221	Dvl3	-0.419777895	0.000758
MEK1_pS217_S221	X53BP1	-0.416869381	0.000832
MEK1_pS217_S221	CD20	-0.397620307	0.001508
MEK1_pS217_S221	TFRC	-0.390534109	0.00186
MEK1_pS217_S221	Caspase_3	-0.390111052	0.001884
MEK1_pS217_S221	PEA15_pS116	-0.387784241	0.002016
MEK1_pS217_S221	p90RSK	-0.386462189	0.002095
MEK1_pS217_S221	Cyclin_B1	-0.386197779	0.002111
MEK1_pS217_S221	Rb_pS807_S811	-0.372818614	0.003086
MEK1_pS217_S221	PI3K_p110_alpha	-0.36964569	0.003369
MEK1_pS217_S221	Rad50	-0.362876785	0.004051
MEK1_pS217_S221	VEGFR2	-0.348439979	0.005925
MEK1_pS217_S221	ADAR1	-0.341353781	0.007096
MEK1_pS217_S221	alpha_Catenin	-0.325647805	0.010437
MEK1_pS217_S221	PKC_alpha_pS657	-0.318085669	0.012483
MEK1_pS217_S221	p70S6K	-0.317186674	0.012748
MEK1_pS217_S221	mTOR_pS2448	-0.305129561	0.016795
MEK1_pS217_S221	GAB2	-0.302591222	0.017775
MEK1_pS217_S221	EGFR	-0.288736118	0.024024
MEK1_pS217_S221	X4E_BP1_pS65	-0.281491274	0.027972
MEK1_pS217_S221	PKC_pan_BetaII_pS660	-0.277207827	0.030552
MEK1_pS217_S221	B_Raf_pS445	-0.274352195	0.032381
MEK1_pS217_S221	Smac	-0.267001586	0.037513
MEK1_pS217_S221	cIAP	-0.265415124	0.038705
MEK1_pS217_S221	C_Raf	-0.265150714	0.038907
MEK1_pS217_S221	A_Raf	-0.258276044	0.044462
MEK1_pS217_S221	MSH2	-0.257112639	0.045464
MEK1_pS217_S221	PCNA	-0.254257007	0.048001

Table S5: ROS responsive gene signature

gene_symbol	padj	P.Value	t	B	log2FoldChange
LYVE1	5.45E-08	1.69E-12	45.812485	18.382896	5.0613415
CPA4	5.83E-08	1.72E-11	35.863809	16.654911	4.0205419
CD109	4.82E-07	1.07E-09	23.130202	13.001456	3.604835
MMP3	5.83E-08	9.11E-12	38.349601	17.154199	3.4939516
RAB31	5.83E-08	5.57E-12	40.389624	17.52721	3.4899815
UCA1	3.55E-07	6.46E-10	24.418658	13.482341	3.4515098
RAB3B	2.55E-06	1.43E-08	17.52768	10.459061	3.4488634
MYOF	1.11E-07	6.87E-11	30.975023	15.501969	3.4303659
IL18	2.84E-07	3.78E-10	25.853008	13.981294	3.3749784
ANXA3	5.83E-08	1.81E-11	35.666787	16.61305	3.3381758
AREG	8.10E-08	4.26E-11	32.582828	15.909013	3.3228094
TSPAN1	6.76E-08	2.81E-11	34.047609	16.255241	3.3190468
LIPH	1.21E-07	8.95E-11	30.120064	15.273068	3.2968108
TSPAN5	8.10E-08	4.05E-11	32.754131	15.950672	3.1661119
CTSE	8.10E-08	4.17E-11	32.658237	15.927391	3.0804131
RBP1	3.44E-07	5.98E-10	24.621531	13.555154	3.0491497
MRGPRX4	6.76E-08	2.93E-11	33.901251	16.221642	2.9427921
GLIPR1	5.83E-08	8.36E-12	38.697289	17.219997	2.9141527
VNN1	1.75E-06	7.97E-09	18.664601	11.044692	2.8727749
S100A11	2.40E-07	2.67E-10	26.823694	14.299079	2.8600142
CYP24A1	2.48E-06	1.35E-08	17.637534	10.517468	2.8573548
TLR4	9.64E-08	5.37E-11	31.795376	15.713347	2.8473897
PLP1	5.84E-07	1.57E-09	22.219565	12.64114	2.8248197
CAPN2	2.94E-07	4.18E-10	25.576543	13.88793	2.8159375
TNS4	1.28E-07	1.00E-10	29.759992	15.173923	2.7761182
C14orf105	3.74E-06	2.39E-08	16.577619	9.9365334	2.7303592
ANXA1	1.43E-07	1.30E-10	28.959781	14.947517	2.7246137
FRMD5	1.14E-07	8.06E-11	30.456606	15.364249	2.7126108
TUBA1A	6.94E-07	1.98E-09	21.673462	12.416335	2.660212
IGKC	9.20E-05	2.40E-06	9.9545727	5.124378	2.6545283
HSD17B3	2.40E-07	2.58E-10	26.922982	14.330731	2.6111201
SERPINE2	6.63E-07	1.85E-09	21.830565	12.481701	2.5182635
QPCT	3.44E-07	6.02E-10	24.600957	13.547805	2.478713
SRPX2	1.10E-07	6.47E-11	31.171376	15.553283	2.4567935
NABP1	1.28E-07	1.07E-10	29.549548	15.115203	2.4035783
SETD7	8.53E-07	2.95E-09	20.762532	12.025826	2.3987384
CDA	1.33E-07	1.15E-10	29.317614	15.049813	2.3969781
LY96	9.29E-06	8.51E-08	14.442051	8.63463	2.3647218
F2RL1	5.83E-08	1.66E-11	35.996695	16.682948	2.3641697
CDKN1A	5.83E-08	1.42E-11	36.586623	16.805517	2.3580363
KITLG	5.28E-07	1.25E-09	22.751245	12.853659	2.2938904

S100A3	1.44E-07	1.38E-10	28.761681	14.890137	2.2898766
LAPTM5	1.14E-07	8.08E-11	30.448365	15.362033	2.2686651
SEL1L3	2.55E-06	1.42E-08	17.543525	10.46751	2.2581679
RASSF2	3.21E-06	1.95E-08	16.949238	10.144722	2.251592
IGFBP7	5.83E-08	1.98E-11	35.324495	16.539478	2.2321601
EMP3	7.43E-07	2.23E-09	21.396458	12.299686	2.2299458
ABRACL	2.40E-07	2.46E-10	27.060785	14.374408	2.227999
VCAN	1.07E-04	3.00E-06	9.7045197	4.887831	2.2223301
TEX15	3.33E-07	5.11E-10	25.034474	13.701036	2.2157789
MR1	5.83E-08	1.26E-11	37.043623	16.898388	2.1998062
TIGAR	3.96E-06	2.61E-08	16.421231	9.8474065	2.1905562
SERPINB8	3.44E-07	6.07E-10	24.579134	13.540001	2.168163
CCND2	3.64E-07	6.96E-10	24.223943	13.411733	2.1565533
MOSPD1	7.21E-07	2.10E-09	21.53634	12.358816	2.1401571
PLA2G2A	3.59E-05	6.10E-07	11.620228	6.5761657	2.1382105
PLK2	4.11E-07	8.14E-10	23.824878	13.264766	2.1257717
GULP1	8.09E-07	2.65E-09	21.005252	12.131839	2.1173517
SLC16A4	2.60E-07	3.06E-10	26.439429	14.175106	2.0955037
SUSD2	1.43E-07	1.32E-10	28.896315	14.929193	2.0943147
KRTAP3-1	3.89E-07	7.58E-10	24.00772	13.332483	2.0908894
DGKA	4.71E-06	3.34E-08	15.989876	9.5967643	2.0907195
GCNT3	2.40E-07	2.48E-10	27.038424	14.367341	2.0904206
UBD	4.28E-05	7.89E-07	11.289554	6.3038872	2.07388
TMEM27	3.33E-07	5.46E-10	24.861502	13.640306	2.0465136
TIMP1	3.55E-07	6.48E-10	24.410642	13.479448	2.0322161
ZNF114	2.67E-07	3.47E-10	26.089776	14.060229	2.0232291
CORO2B	3.64E-07	6.98E-10	24.216885	13.40916	2.0201252
ANKRD1	2.40E-06	1.24E-08	17.800391	10.603319	2.01931
CD24	1.36E-04	4.22E-06	9.3313743	4.5248153	2.010869
CLDN6	5.75E-07	1.42E-09	22.455552	12.736223	2.0038543
