# Supplementary figures and images for: MEK1 drives oncogenic signaling and interacts with PARP1 for genomic and metabolic homeostasis in malignant pleural mesothelioma
Source: Cell Death Discov. 2023 Feb 10;9:55. doi: 10.1038/s41420-023-01307-2 (PMC9918536; doi:10.1038/s41420-023-01307-2)

**Fig. 1E**

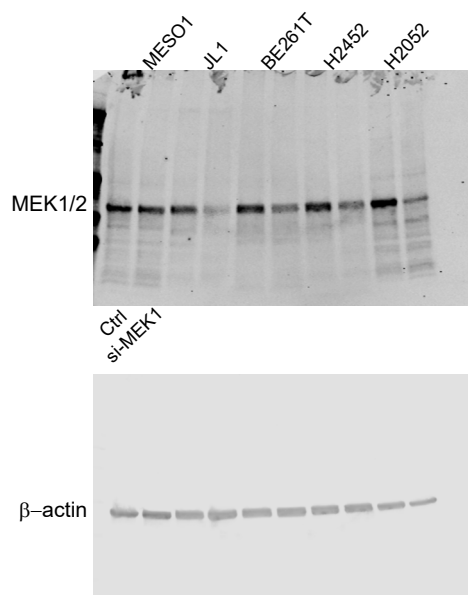

**Fig. 3E**

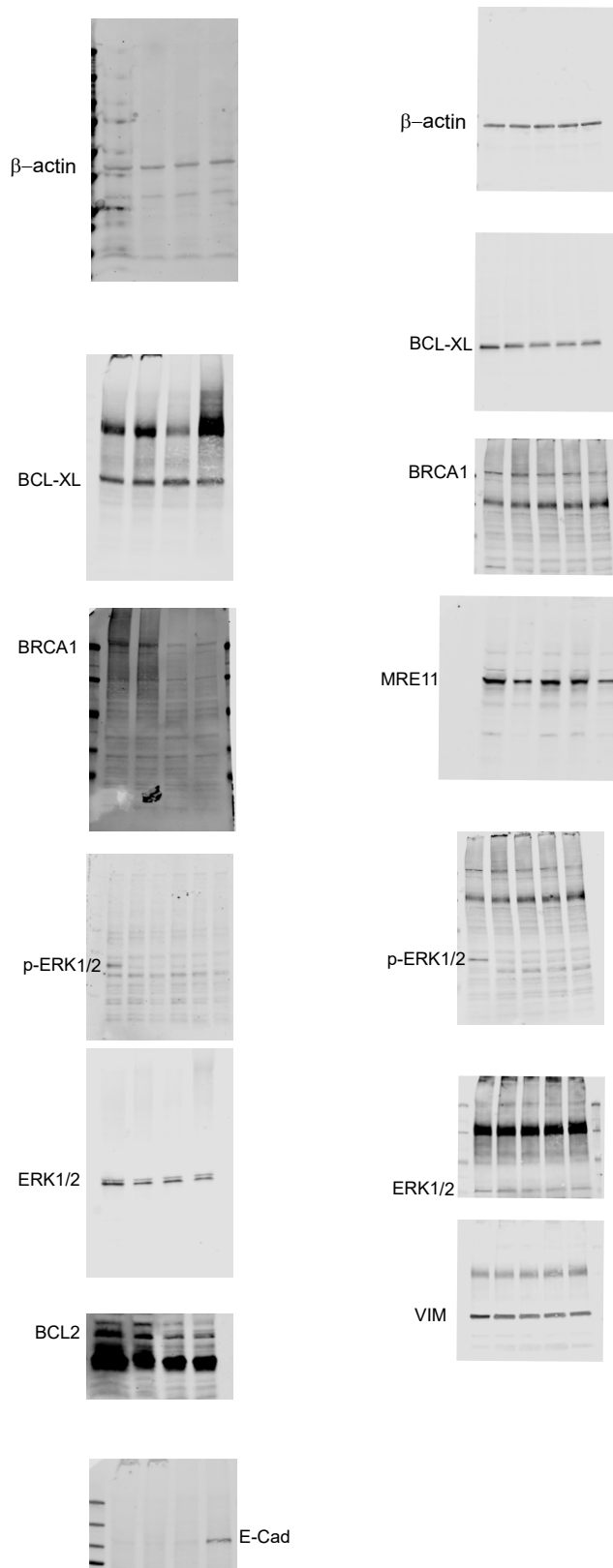

**Fig. 2F**

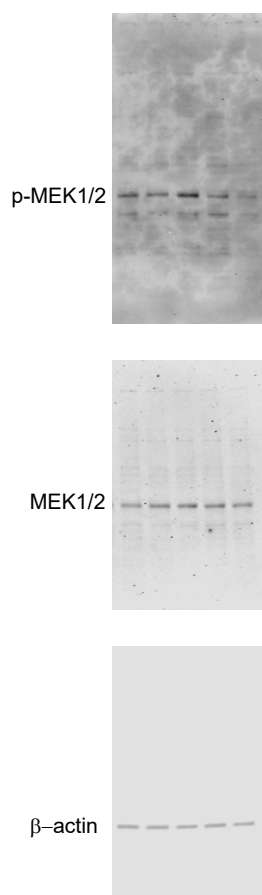

**Fig. 3F**

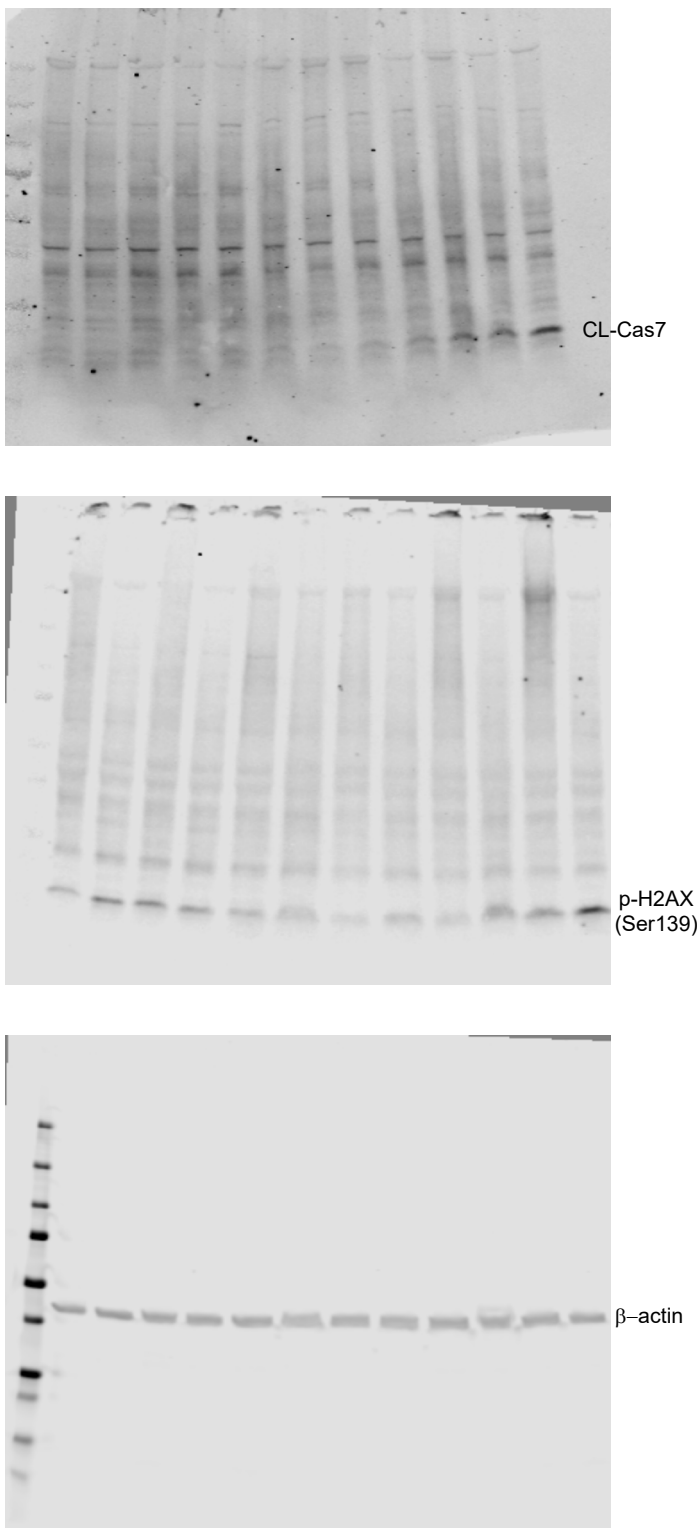

**Fig. 3G**

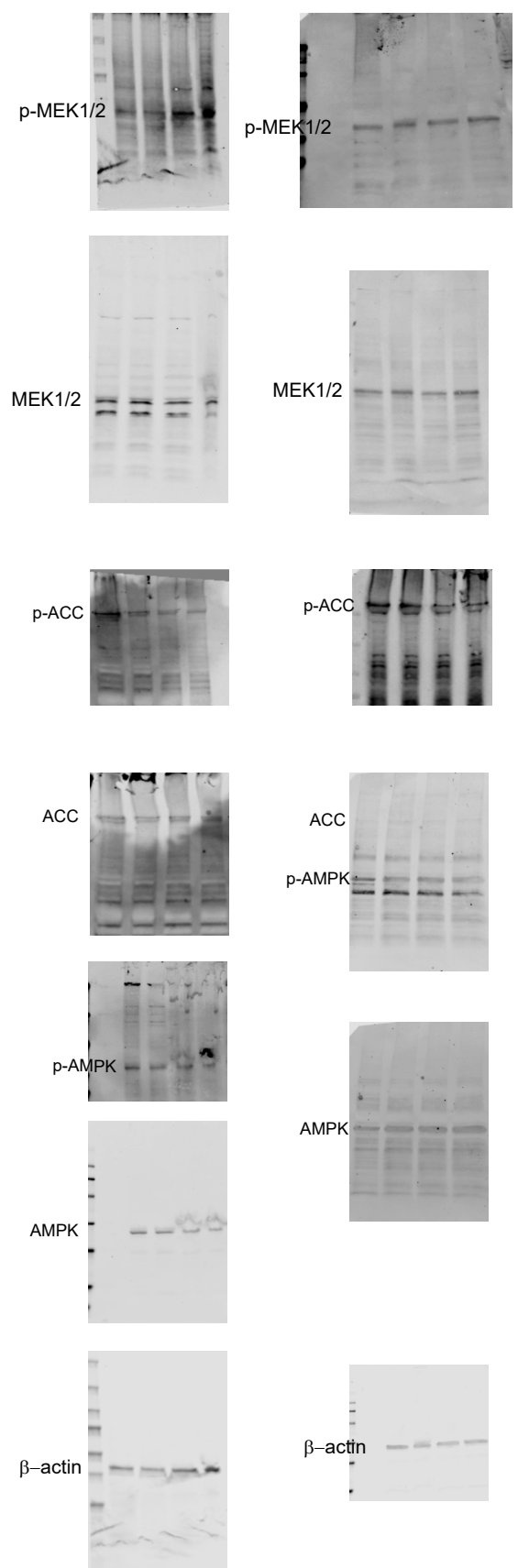

**Fig. 4F**

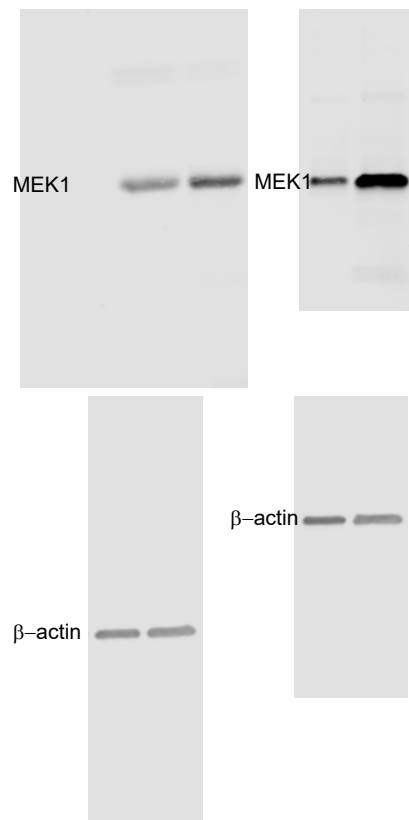

**Fig. 5I**

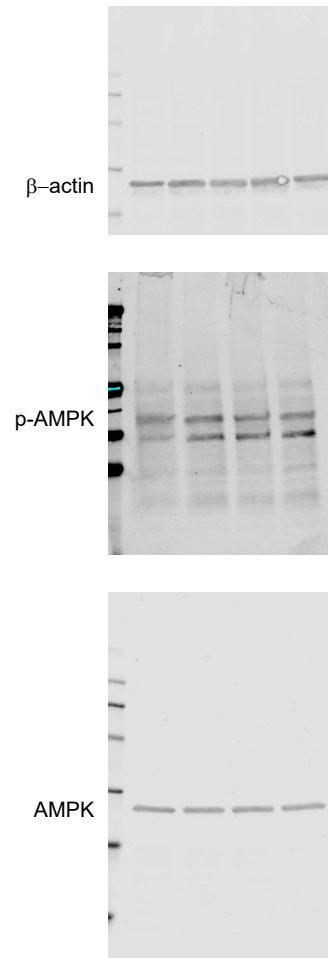

Supplement: Supplementary file 2 — Original Data File [file 41420_2023_1307_MOESM2_ESM.pdf]
