# Supplementary material for: MEK1 drives oncogenic signaling and interacts with PARP1 for genomic and metabolic homeostasis in malignant pleural mesothelioma
Source: Cell Death Discov. 2023 Feb 10;9:55. doi: 10.1038/s41420-023-01307-2 (PMC9918536; doi:10.1038/s41420-023-01307-2)
Supplement: Supplementary file 3 — Authorship change agreement [file 41420_2023_1307_MOESM3_ESM.pdf]

Peng, Renwang

From: Haitang YANG <yanghaitang@shchest.org>
Sent: Friday, December 23, 2022 7:55 AM
To: Peng, Renwang
Subject: 回复:FW: CDDISCOVERY-22-5205R1 Initial Quality Check

Great. I agree with the authorship.

Best,

Haitang

该邮件从移动设备发送

--------------原始邮件--------------
发件人："Peng, Renwang "<Renwang.Peng@insel.ch>;
发送时间：2022 年 12 月 23 日(星期五) 凌晨 1:08
收件人："Haitang YANG" <yanghaitang@shchest.org>;"yanyun.gao" <yanyun.gao@dbmr.unibe.ch>;"xuduo" <xuduo@jsph.org.cn>;"许可" <xukeqaz1997@126.com>;"Liang, Shun-Qing" <Shun-Qing.Liang@umassmed.edu>;"fjxh_zhang2021@163.com" <fjxh_zhang2021@163.com>;"Hall, Sean" <Sean.Hall@wyss.harvard.edu>;"AminaScherz" <amina.scherz@icloud.com>;"Forster, Stefan (DBMR)" <stefan.forster@dbmr.unibe.ch>;"Berezowska Sabina" <Sabina.Berezowska@chuv.ch>;"Feng Yao" <yaofeng6796678@126.com>;"Ochsenbein,Adrian" <Adrian.Ochsenbein@insel.ch>;"Marti, Thomas Michael" <Thomas.Marti@insel.ch>;"gregor.kocher@hin.ch" <gregor.kocher@hin.ch>;"Ralph Alexander Schmid" <ralph_a_schmid@hotmail.com>;"Dorn, Patrick" <Patrick.Dorn@insel.ch>;
主题：FW: CDDISCOVERY-22-5205R1 Initial Quality Check

Dear co-authors,

Good news: **our manuscript has been provisionally accepted!**

As indicated in the email below, Mr. Ke XU (Shanghai Chest Hospital), who performed the in vivo mice experiments for the revision, has been added as a new author.

So one more step towards full acceptance is still to come:

@All of you: please reply to me if you agree with this change.

@Prof. Schmid and Patrick: please link your ORCID with your accounts on the MTS as described in the email.

Thank you very much for the fruitful collaboration! I wish you have happy holidays and a prosperous 2023.

Ren

From: cddiscovery@springernature.com <cddiscovery@springernature.com>
Sent: Thursday, December 22, 2022 5:01 PM
To: Peng, Renwang <Renwang.Peng@insel.ch>
Subject: CDDISCOVERY-22-5205R1 Initial Quality Check

Manuscript Number: CDDISCOVERY-22-5205R1

Title: MEK1 drives oncogenic signalling and interacts with PARP1 for genomic and metabolic homeostasis in malignant pleural mesothelioma

Authors: Ren-Wang Peng, Haitang Yang, Yanyun Gao, Duo Xu, Ke Xu, Shunqing Liang, Zhang Yang, Amina Scherz, Sean Hall, Stefan Forster, Sabina Berezowska, Feng Yao, Adrian Ochsenbein, Thomas Marti, Gregor Kocher, Ralph Schmid, Patrick Dorn

Dear Professor Peng,

After carrying out a quality check of your manuscript submitted to *Cell Death Discovery*, it has come to our attention that the following points need to be addressed before we are able to pass it onto the Editors for their consideration:

1. It has come to our attention that your most recent author list differs from the one in your original submission.

Please request agreement from all authors including additions and deletions, these can be collected in the following way:Email your co-authors with the change, and ask them to reply to your email confirming that they agree to these changes. Once you have collected these replies, please combine all of the co-authors' email responses in one document and upload this file to your submission.

2.Please make sure all corresponding authors link their own ORCID.
As part of our efforts to improve transparency in authorship, we now request that all authors identified as 'corresponding author' create and link their Open Researcher and Contributor Identifier (ORCID) with their account on the Manuscript Tracking System (MTS) prior to acceptance. For more information please visit http://www.springernature.com/orcid

Please follow the steps below to link your ORCID to your account on our MTS. Please ensure the ORCID is linked to your account associated with this manuscript, and not to any other account you may have on our system. If you do not yet have an ORCID you will be able to create one in minutes. If you are not listed as a corresponding author, please ensure that the corresponding author(s) comply.

1. From the home page of the MTS (http://mts-cddiscovery.nature.com/cgi-bin/main.plex) click on 'Modify my Springer Nature account' under 'General tasks'.
2. In the 'Personal profile' tab, click on 'ORCID Create/link an Open Researcher Contributor ID(ORCID)'. This will redirect you to the ORCID website.
3a. If you already have an ORCID account, enter your ORCID email and password and click on 'Authorize' to link your ORCID with your account on the MTS.

3b. If you don't yet have an ORCID, you can easily create one by providing the required information and then click on 'Authorize'. This will link your newly created ORCID with your account on the MTS.

Yours sincerely,

Editorial Office
Cell Death & Discovery
cddiscovery@springernature.com

This email has been sent through the Springer Nature Manuscript Tracking System NY-610A-NPG&MTS

Confidentiality Statement:

This e-mail is confidential and subject to copyright. Any unauthorised use or disclosure of its contents is prohibited. If you have received this email in error please notify our Manuscript Tracking System Helpdesk team at http://platformsupport.nature.com .

Details of the confidentiality and pre-publicity policy may be found here http://www.nature.com/authors/policies/confidentiality.html

Privacy Policy | Update Profile

Peng, Renwang

From: Gao, Yanyun (DBMR) <yanyun.gao@unibe.ch>
Sent: Friday, December 23, 2022 8:54 AM
To: Peng, Renwang; Haitang YANG; xuduo@jsph.org.cn; 许可; Liang, Shun-Qing; fjxh_zhang2021@163.com; Hall, Sean; Amina Scherz; Forster, Stefan (DBMR); Berezowska Sabina; Feng Yao; Ochsenbein, Adrian; Marti, Thomas Michael; gregor.kocher@hin.ch; Ralph Alexander Schmid; Dorn, Patrick
Subject: RE: CDDISCOVERY-22-5205R1 Initial Quality Check [HIN secured]

Hi Ren,

I agree with the authorship change.

Best regards,
Yanyun

From: Peng, Renwang <Renwang.Peng@insel.ch>
Sent: Thursday, December 22, 2022 6:08 PM
To: Haitang YANG <yanghaitang@shchest.org>; Gao, Yanyun (DBMR) <yanyun.gao@unibe.ch>; xuduo@jsph.org.cn; 许可 <xukeqaz1997@126.com>; Liang, Shun-Qing <Shun-Qing.Liang@umassmed.edu>; fjxh_zhang2021@163.com; Hall, Sean <Sean.Hall@wyss.harvard.edu>; Amina Scherz <amina.scherz@icloud.com>; Forster, Stefan (DBMR) <stefan.forster@unibe.ch>; Berezowska Sabina <Sabina.Berezowska@chuv.ch>; Feng Yao <yaofeng6796678@126.com>; Ochsenbein, Adrian <Adrian.Ochsenbein@insel.ch>; Marti, Thomas Michael <Thomas.Marti@insel.ch>; gregor.kocher@hin.ch; Ralph Alexander Schmid <ralph_a_schmid@hotmail.com>; Dorn, Patrick <Patrick.Dorn@insel.ch>
Subject: FW: CDDISCOVERY-22-5205R1 Initial Quality Check

Dear co-authors,

Good news: **our manuscript has been provisionally accepted!**

As indicated in the email below, Mr. Ke XU (Shanghai Chest Hospital), who performed the in vivo mice experiments for the revision, has been added as a new author.

So one more step towards full acceptance is still to come:

@All of you: please reply to me if you agree with this change.

@Prof. Schmid and Patrick: please link your ORCID with your accounts on the MTS as described in the email.

Thank you very much for the fruitful collaboration! I wish you have happy holidays and a prosperous 2023.

Ren

From: cddiscovery@springernature.com <cddiscovery@springernature.com>
Sent: Thursday, December 22, 2022 5:01 PM

To: Peng, Renwang <Renwang.Peng@insel.ch>
Subject: CDDISCOVERY-22-5205R1 Initial Quality Check

Manuscript Number: CDDISCOVERY-22-5205R1

Title: MEK1 drives oncogenic signalling and interacts with PARP1 for genomic and metabolic homeostasis in malignant pleural mesothelioma

Authors: Ren-Wang Peng, Haitang Yang, Yanyun Gao, Duo Xu, Ke Xu, Shunqing Liang, Zhang Yang, Amina Scherz, Sean Hall, Stefan Forster, Sabina Berezowska, Feng Yao, Adrian Ochsenbein, Thomas Marti, Gregor Kocher, Ralph Schmid, Patrick Dorn

Dear Professor Peng,

After carrying out a quality check of your manuscript submitted to *Cell Death Discovery*, it has come to our attention that the following points need to be addressed before we are able to pass it onto the Editors for their consideration:

1. It has come to our attention that your most recent author list differs from the one in your original submission.

Please request agreement from all authors including additions and deletions, these can be collected in the following way:Email your co-authors with the change, and ask them to reply to your email confirming that they agree to these changes. Once you have collected these replies, please combine all of the co-authors' email responses in one document and upload this file to your submission.

2.Please make sure all corresponding authors link their own ORCID.
As part of our efforts to improve transparency in authorship, we now request that all authors identified as 'corresponding author' create and link their Open Researcher and Contributor Identifier (ORCID) with their account on the Manuscript Tracking System (MTS) prior to acceptance. For more information please visit http://www.springernature.com/orcid
Please follow the steps below to link your ORCID to your account on our MTS. Please ensure the ORCID is linked to your account associated with this manuscript, and not to any other account you may have on our system. If you do not yet have an ORCID you will be able to create one in minutes. If you are not listed as a corresponding author, please ensure that the corresponding author(s) comply.

1. From the home page of the MTS (http://mts-cddiscovery.nature.com/cgi-bin/main.plex) click on 'Modify my Springer Nature account' under 'General tasks'.
2. In the 'Personal profile' tab, click on 'ORCID Create/link an Open Researcher Contributor ID(ORCID)'. This will redirect you to the ORCID website.
3a. If you already have an ORCID account, enter your ORCID email and password and click on 'Authorize' to link your ORCID with your account on the MTS.

3b. If you don't yet have an ORCID, you can easily create one by providing the required information and then click on 'Authorize'. This will link your newly created ORCID with your account on the MTS.

Yours sincerely,

Editorial Office
Cell Death & Discovery
cddiscovery@springernature.com

This email has been sent through the Springer Nature Manuscript Tracking System NY-610A-NPG&MTS

Confidentiality Statement:

This e-mail is confidential and subject to copyright. Any unauthorised use or disclosure of its contents is prohibited. If you have received this email in error please notify our Manuscript Tracking System Helpdesk team at http://platformsupport.nature.com .

Details of the confidentiality and pre-publicity policy may be found here http://www.nature.com/authors/policies/confidentiality.html

Privacy Policy | Update Profile

Peng, Renwang

From: 许多 <xuduo@jsph.org.cn>
Sent: Saturday, December 24, 2022 2:37 AM
To: Liang, Shun-Qing
Cc: Berezowska Sabina; Peng, Renwang; Haitang YANG; Gao, Yanyun (DBMR) (yanyun.gao@dbmr.unibe.ch) (yanyun.gao@dbmr.unibe.ch); 许可; fjxh_zhang2021@163.com; Hall, Sean; Amina Scherz; Forster, Stefan (DBMR); Feng Yao; Ochsenbein, Adrian; Marti, Thomas Michael; gregor.kocher@hin.ch; Ralph Alexander Schmid; Dorn, Patrick
Subject: Re: CDDISCOVERY-22-5205R1 Initial Quality Check

Dear Ren,

I agree with the authorship.

Merry Christmas and happy new year！

Best Regards

Duo

Duo Xu, MD-PhD

Attending Physician;Postdoctoral Fellow

Department of Oncology, The First Affiliated Hospital of Nanjing Medical University

No.300 Guangzhou Road, 210029, Nanjing, Jiangsu
China

E-Mail:xuduo@jsph.org.cn

---- Replied Message ----

From	Liang, Shun-Qing<shun-qing.liang@umassmed.edu>
Date	12/23/2022 19:37
To	Berezowska Sabina <sabina.berezowska@chuv.ch>,<br>Peng, Renwang<renwang.peng@insel.ch>,<br>Haitang YANG <yanghaitang@shchest.org>,<br>Gao, Yanyun (DBMR) (yanyun.gao@dbmr.unibe.ch) (yanyun.gao@dbmr.unibe.ch)<yanyun.gao@dbmr.unibe.ch>,<br>xuduo@jsph.org.cn<xuduo@jsph.org.cn>,<br>许可<xukeqaz1997@126.com>,<br>fjxh_zhang2021@163.com<fjxh_zhang2021@163.com>,<br>Hall, Sean<sean.hall@wyss.harvard.edu>,<br>Amina Scherz <amina.scherz@icloud.com>,<br>Forster, Stefan (DBMR)<stefan.forster@dbmr.unibe.ch>,<br>Feng Yao <yaofeng6796678@126.com>,<br>Ochsenbein, Adrian<adrian.ochsenbein@insel.ch>,<br>Marti, Thomas Michael<thomas.marti@insel.ch>,<br>gregor.kocher@hin.ch<gregor.kocher@hin.ch>,

Ralph Alexander Schmid <ralph_a_schmid@hotmail.com>,
Dorn, Patrick<patrick.dorn@insel.ch>

Subject Re: CDDISCOVERY-22-5205R1 Initial Quality Check

Dear Ren,

I agree with the authorship.
Merry Christmas to all.

Best Regards
Simon

From: Berezowska Sabina <Sabina.Berezowska@chuv.ch>
Sent: Friday, December 23, 2022 3:43:43 AM
To: Peng, Renwang <Renwang.Peng@insel.ch>; Haitang YANG <yanghaitang@shchest.org>; Gao, Yanyun (DBMR) (yanyun.gao@dbmr.unibe.ch) (yanyun.gao@dbmr.unibe.ch) <yanyun.gao@dbmr.unibe.ch>; xuduo@jsph.org.cn <xuduo@jsph.org.cn>; 许可 <xukeqaz1997@126.com>; Liang, Shun-Qing <Shun-Qing.Liang@umassmed.edu>; fjxh_zhang2021@163.com <fjxh_zhang2021@163.com>; Hall, Sean <Sean.Hall@wyss.harvard.edu>; Amina Scherz <amina.scherz@icloud.com>; Forster, Stefan (DBMR) <stefan.forster@dbmr.unibe.ch>; Feng Yao <yaofeng6796678@126.com>; Ochsenbein, Adrian <Adrian.Ochsenbein@insel.ch>; Marti, Thomas Michael <Thomas.Marti@insel.ch>; gregor.kocher@hin.ch <gregor.kocher@hin.ch>; Ralph Alexander Schmid <ralph_a_schmid@hotmail.com>; Dorn, Patrick <Patrick.Dorn@insel.ch>
Subject: RE: CDDISCOVERY-22-5205R1 Initial Quality Check

Dear Ren
Congratulation ! Impressive, and a really a nice Christmas present!
Of course, I agree with the authorship change.

Wishing you all a very merry holiday season and All the Best for 2013!
Sabina

De : Peng, Renwang <Renwang.Peng@insel.ch>
Envoyé : jeudi, 22 décembre 2022 18:08
À : Haitang YANG <yanghaitang@shchest.org>; Gao, Yanyun (DBMR) (yanyun.gao@dbmr.unibe.ch) (yanyun.gao@dbmr.unibe.ch) <yanyun.gao@dbmr.unibe.ch>; xuduo@jsph.org.cn; 许可 <xukeqaz1997@126.com>; Liang, Shun-Qing <Shun-Qing.Liang@umassmed.edu>; fjxh_zhang2021@163.com; Hall, Sean <Sean.Hall@wyss.harvard.edu>; Amina Scherz <amina.scherz@icloud.com>; Forster, Stefan (DBMR) <stefan.forster@dbmr.unibe.ch>; Berezowska Sabina <Sabina.Berezowska@chuv.ch>; Feng Yao <yaofeng6796678@126.com>; Ochsenbein, Adrian <Adrian.Ochsenbein@insel.ch>; Marti, Thomas Michael <Thomas.Marti@insel.ch>; gregor.kocher@hin.ch; Ralph Alexander Schmid <ralph_a_schmid@hotmail.com>; Dorn, Patrick <Patrick.Dorn@insel.ch>
Objet : FW: CDDISCOVERY-22-5205R1 Initial Quality Check [HIN secured]

Dear co-authors,

Good news: **our manuscript has been provisionally accepted!**

As indicated in the email below, Mr. Ke XU (Shanghai Chest Hospital), who performed the in vivo mice experiments for the revision, has been added as a new author.

So one more step towards full acceptance is still to come:

@All of you: please reply to me if you agree with this change.

@Prof. Schmid and Patrick: please link your ORCID with your accounts on the MTS as described in the email.

Thank you very much for the fruitful collaboration! I wish you have happy holidays and a prosperous 2023.

Ren

From: cddiscovery@springernature.com <cddiscovery@springernature.com>
Sent: Thursday, December 22, 2022 5:01 PM
To: Peng, Renwang <Renwang.Peng@insel.ch>
Subject: CDDISCOVERY-22-5205R1 Initial Quality Check

Manuscript Number: CDDISCOVERY-22-5205R1

Title: MEK1 drives oncogenic signalling and interacts with PARP1 for genomic and metabolic homeostasis in malignant pleural mesothelioma

Authors: Ren-Wang Peng, Haitang Yang, Yanyun Gao, Duo Xu, Ke Xu, Shunqing Liang, Zhang Yang, Amina Scherz, Sean Hall, Stefan Forster, Sabina Berezowska, Feng Yao, Adrian Ochsenbein, Thomas Marti, Gregor Kocher, Ralph Schmid, Patrick Dorn

Dear Professor Peng,

After carrying out a quality check of your manuscript submitted to *Cell Death Discovery*, it has come to our attention that the following points need to be addressed before we are able to pass it onto the Editors for their consideration:

1. It has come to our attention that your most recent author list differs from the one in your original submission.

Please request agreement from all authors including additions and deletions, these can be collected in the following way:Email your co-authors with the change, and ask them to reply to your email confirming that they agree to these changes. Once you have collected these replies, please combine all of the co-authors' email responses in one document and upload this file to your submission.

2.Please make sure all corresponding authors link their own ORCID.
As part of our efforts to improve transparency in authorship, we now request that all authors identified as 'corresponding author' create and link their Open Researcher and Contributor Identifier (ORCID) with their account on the Manuscript Tracking System (MTS) prior to acceptance. For more information please visit http://www.springernature.com/orcid
Please follow the steps below to link your ORCID to your account on our MTS. Please ensure the ORCID is linked to your account associated with this manuscript, and not to any other account you may have on our system. If you do not yet have an ORCID you will be able to create one in minutes. If you are not listed as a corresponding author, please ensure that the corresponding author(s) comply.

1. From the home page of the MTS (http://mts-cddiscovery.nature.com/cgi-bin/main.plex) click on 'Modify my Springer Nature account' under 'General tasks'.
2. In the 'Personal profile' tab, click on 'ORCID Create/link an Open Researcher Contributor ID(ORCID)'.

This will redirect you to the ORCID website.
3a. If you already have an ORCID account, enter your ORCID email and password and click on 'Authorize' to link your ORCID with your account on the MTS.

3b. If you don't yet have an ORCID, you can easily create one by providing the required information and then click on 'Authorize'. This will link your newly created ORCID with your account on the MTS.

Yours sincerely,

Editorial Office
Cell Death & Discovery
cddiscovery@springernature.com

This email has been sent through the Springer Nature Manuscript Tracking System NY-610A-NPG&MTS

Confidentiality Statement:

This e-mail is confidential and subject to copyright. Any unauthorised use or disclosure of its contents is prohibited. If you have received this email in error please notify our Manuscript Tracking System Helpdesk team at http://platformsupport.nature.com .

Details of the confidentiality and pre-publicity policy may be found here http://www.nature.com/authors/policies/confidentiality.html

Privacy Policy | Update Profile

Peng, Renwang

From: 许可 <xukeqaz@sjtu.edu.cn>
Sent: Saturday, December 24, 2022 10:05 AM
To: Peng, Renwang
Cc: yaofeng@shsmu.edu.cn
Subject: Re: CDDISCOVERY-22-5205R1 Initial Quality Check

Dear Prof. Ren-Wang Peng:

Thank you for adding me as a new author. It is my great honor to participate in this great work! Really appreciate your help!

I agree with this change！

Thanks again and happy new year！
Best regards,

Xu Ke

----- 原始邮件 -----
发件人: "Peng, Renwang" <Renwang.Peng@insel.ch>
收件人: yaofeng@shsmu.edu.cn, xukeqaz@sjtu.edu.cn
发送时间: 星期六, 2022 年 12 月 24 日 下午 4:50:51
主题: FW: CDDISCOVERY-22-5205R1 Initial Quality Check

From: Peng, Renwang
Sent: Thursday, December 22, 2022 6:08 PM
To: 'Haitang YANG' <yanghaitang@shchest.org>; Gao, Yanyun (DBMR) (yanyun.gao@dbmr.unibe.ch) (yanyun.gao@dbmr.unibe.ch) <yanyun.gao@dbmr.unibe.ch>; 'xuduo@jsph.org.cn' <xuduo@jsph.org.cn>; '许可' <xukeqaz1997@126.com>; 'Liang, Shun-Qing' <Shun-Qing.Liang@umassmed.edu>; 'fjxh_zhang2021@163.com' <fjxh_zhang2021@163.com>; 'Hall, Sean' <Sean.Hall@wyss.harvard.edu>; Amina Scherz <amina.scherz@icloud.com>; Forster, Stefan (DBMR) <stefan.forster@dbmr.unibe.ch>; 'Berezowska Sabina' <Sabina.Berezowska@chuv.ch>; Feng Yao <yaofeng6796678@126.com>; Ochsenbein, Adrian <Adrian.Ochsenbein@insel.ch>; Marti, Thomas Michael <Thomas.Marti@insel.ch>; 'gregor.kocher@hin.ch' <gregor.kocher@hin.ch>; Ralph Alexander Schmid <ralph_a_schmid@hotmail.com>; Dorn, Patrick <Patrick.Dorn@insel.ch>
Subject: FW: CDDISCOVERY-22-5205R1 Initial Quality Check

Dear co-authors,

Good news: our manuscript has been provisionally accepted!

As indicated in the email below, Mr. Ke XU (Shanghai Chest Hospital), who performed the in vivo mice experiments for the revision, has been added as a new author.

So one more step towards full acceptance is still to come:

@All of you: please reply to me if you agree with this change.

@Prof. Schmid and Patrick: please link your ORCID with your accounts on the MTS as described in the email.

Thank you very much for the fruitful collaboration! I wish you have happy holidays and a prosperous 2023.

Ren

From: cddiscovery@springernature.com<mailto:cddiscovery@springernature.com>
<cddiscovery@springernature.com<mailto:cddiscovery@springernature.com>>
Sent: Thursday, December 22, 2022 5:01 PM
To: Peng, Renwang <Renwang.Peng@insel.ch<mailto:Renwang.Peng@insel.ch>>
Subject: CDDISCOVERY-22-5205R1 Initial Quality Check

Manuscript Number: CDDISCOVERY-22-5205R1

Title: MEK1 drives oncogenic signalling and interacts with PARP1 for genomic and metabolic homeostasis in malignant pleural mesothelioma

Authors: Ren-Wang Peng, Haitang Yang, Yanyun Gao, Duo Xu, Ke Xu, Shunqing Liang, Zhang Yang, Amina Scherz, Sean Hall, Stefan Forster, Sabina Berezowska, Feng Yao, Adrian Ochsenbein, Thomas Marti, Gregor Kocher, Ralph Schmid, Patrick Dorn

Dear Professor Peng,

After carrying out a quality check of your manuscript submitted to Cell Death Discovery, it has come to our attention that the following points need to be addressed before we are able to pass it onto the Editors for their consideration:

1. It has come to our attention that your most recent author list differs from the one in your original submission.

Please request agreement from all authors including additions and deletions, these can be collected in the following way:Email your co-authors with the change, and ask them to reply to your email confirming that they agree to these changes. Once you have collected these replies, please combine all of the co-authors' email responses in one document and upload this file to your submission.

2.Please make sure all corresponding authors link their own ORCID.
As part of our efforts to improve transparency in authorship, we now request that all authors identified as 'corresponding author' create and link their Open Researcher and Contributor Identifier (ORCID) with their account on the Manuscript Tracking System (MTS) prior to acceptance. For more information please visit http://www.springernature.com/orcid
Please follow the steps below to link your ORCID to your account on our MTS. Please ensure the ORCID is linked to your account associated with this manuscript, and not to any other account you may have on our system. If you do not yet have an ORCID you will be able to create one in minutes. If you are not listed as a corresponding author, please ensure that the corresponding author(s) comply.
1. From the home page of the MTS (http://mts-cddiscovery.nature.com/cgi-bin/main.plex) click on 'Modify my Springer Nature account' under 'General tasks'.
2. In the 'Personal profile' tab, click on 'ORCID Create/link an Open Researcher Contributor ID(ORCID)'. This will redirect you to the ORCID website.
3a. If you already have an ORCID account, enter your ORCID email and password and click on 'Authorize' to link your ORCID with your account on the MTS.

3b. If you don't yet have an ORCID, you can easily create one by providing the required information and then click on 'Authorize'. This will link your newly created ORCID with your account on the MTS.

Yours sincerely,

Editorial Office
Cell Death & Discovery
cddiscovery@springernature.com<mailto:cddiscovery@springernature.com>

This email has been sent through the Springer Nature Manuscript Tracking System NY-610A-NPG&MTS

Confidentiality Statement:

This e-mail is confidential and subject to copyright. Any unauthorised use or disclosure of its contents is prohibited. If you have received this email in error please notify our Manuscript Tracking System Helpdesk team at http://platformsupport.nature.com .

Details of the confidentiality and pre-publicity policy may be found here http://www.nature.com/authors/policies/confidentiality.html

Privacy Policy<http://www.nature.com/info/privacy.html> | Update Profile<https://mts-cdd.nature.com>

Peng, Renwang

From: Liang, Shun-Qing <Shun-Qing.Liang@umassmed.edu>
Sent: Friday, December 23, 2022 12:38 PM
To: Berezowska Sabina; Peng, Renwang; Haitang YANG; Gao, Yanyun (DBMR) (yanyun.gao@dbmr.unibe.ch) (yanyun.gao@dbmr.unibe.ch); xuduo@jsph.org.cn; 许可; fjxh_zhang2021@163.com; Hall, Sean; Amina Scherz; Forster, Stefan (DBMR); Feng Yao; Ochsenbein, Adrian; Marti, Thomas Michael; gregor.kocher@hin.ch; Ralph Alexander Schmid; Dorn, Patrick
Subject: Re: CDDISCOVERY-22-5205R1 Initial Quality Check

Dear Ren,

I agree with the authorship.
Merry Christmas to all.

Best Regards
Simon

From: Berezowska Sabina <Sabina.Berezowska@chuv.ch>
Sent: Friday, December 23, 2022 3:43:43 AM
To: Peng, Renwang <Renwang.Peng@insel.ch>; Haitang YANG <yanghaitang@shchest.org>; Gao, Yanyun (DBMR) (yanyun.gao@dbmr.unibe.ch) (yanyun.gao@dbmr.unibe.ch) <yanyun.gao@dbmr.unibe.ch>; xuduo@jsph.org.cn <xuduo@jsph.org.cn>; 许可 <xukeqaz1997@126.com>; Liang, Shun-Qing <Shun-Qing.Liang@umassmed.edu>; fjxh_zhang2021@163.com <fjxh_zhang2021@163.com>; Hall, Sean <Sean.Hall@wyss.harvard.edu>; Amina Scherz <amina.scherz@icloud.com>; Forster, Stefan (DBMR) <stefan.forster@dbmr.unibe.ch>; Feng Yao <yaofeng6796678@126.com>; Ochsenbein, Adrian <Adrian.Ochsenbein@insel.ch>; Marti, Thomas Michael <Thomas.Marti@insel.ch>; gregor.kocher@hin.ch <gregor.kocher@hin.ch>; Ralph Alexander Schmid <ralph_a_schmid@hotmail.com>; Dorn, Patrick <Patrick.Dorn@insel.ch>
Subject: RE: CDDISCOVERY-22-5205R1 Initial Quality Check

Dear Ren
Congratulation ! Impressive, and a really a nice Christmas present!
Of course, I agree with the authorship change.

Wishing you all a very merry holiday season and All the Best for 2013!
Sabina

De : Peng, Renwang <Renwang.Peng@insel.ch>
Envoyé : jeudi, 22 décembre 2022 18:08
À : Haitang YANG <yanghaitang@shchest.org>; Gao, Yanyun (DBMR) (yanyun.gao@dbmr.unibe.ch) (yanyun.gao@dbmr.unibe.ch) <yanyun.gao@dbmr.unibe.ch>; xuduo@jsph.org.cn; 许可 <xukeqaz1997@126.com>; Liang, Shun-Qing <Shun-Qing.Liang@umassmed.edu>; fjxh_zhang2021@163.com; Hall, Sean <Sean.Hall@wyss.harvard.edu>; Amina Scherz <amina.scherz@icloud.com>; Forster, Stefan (DBMR) <stefan.forster@dbmr.unibe.ch>; Berezowska Sabina <Sabina.Berezowska@chuv.ch>; Feng Yao <yaofeng6796678@126.com>; Ochsenbein, Adrian <Adrian.Ochsenbein@insel.ch>; Marti, Thomas Michael <Thomas.Marti@insel.ch>; gregor.kocher@hin.ch; Ralph Alexander Schmid <ralph_a_schmid@hotmail.com>; Dorn, Patrick <Patrick.Dorn@insel.ch>
Objet : FW: CDDISCOVERY-22-5205R1 Initial Quality Check [HIN secured]

Dear co-authors,

Good news: **our manuscript has been provisionally accepted!**

As indicated in the email below, Mr. Ke XU (Shanghai Chest Hospital), who performed the in vivo mice experiments for the revision, has been added as a new author.

So one more step towards full acceptance is still to come:

@All of you: please reply to me if you agree with this change.

@Prof. Schmid and Patrick: please link your ORCID with your accounts on the MTS as described in the email.

Thank you very much for the fruitful collaboration! I wish you have happy holidays and a prosperous 2023.

Ren

From: cddiscovery@springernature.com <cddiscovery@springernature.com>
Sent: Thursday, December 22, 2022 5:01 PM
To: Peng, Renwang <Renwang.Peng@insel.ch>
Subject: CDDISCOVERY-22-5205R1 Initial Quality Check

Manuscript Number: CDDISCOVERY-22-5205R1

Title: MEK1 drives oncogenic signalling and interacts with PARP1 for genomic and metabolic homeostasis in malignant pleural mesothelioma

Authors: Ren-Wang Peng, Haitang Yang, Yanyun Gao, Duo Xu, Ke Xu, Shunqing Liang, Zhang Yang, Amina Scherz, Sean Hall, Stefan Forster, Sabina Berezowska, Feng Yao, Adrian Ochsenbein, Thomas Marti, Gregor Kocher, Ralph Schmid, Patrick Dorn

Dear Professor Peng,

After carrying out a quality check of your manuscript submitted to *Cell Death Discovery*, it has come to our attention that the following points need to be addressed before we are able to pass it onto the Editors for their consideration:

1. It has come to our attention that your most recent author list differs from the one in your original submission.

Please request agreement from all authors including additions and deletions, these can be collected in the following way:Email your co-authors with the change, and ask them to reply to your email confirming that they agree to these changes. Once you have collected these replies, please combine all of the co-authors' email responses in one document and upload this file to your submission.

2.Please make sure all corresponding authors link their own ORCID.
As part of our efforts to improve transparency in authorship, we now request that all authors identified as 'corresponding author' create and link their Open Researcher and Contributor Identifier (ORCID) with their account on the Manuscript Tracking System (MTS) prior to acceptance. For more information please visit

http://www.springernature.com/orcid
Please follow the steps below to link your ORCID to your account on our MTS. Please ensure the ORCID is linked to your account associated with this manuscript, and not to any other account you may have on our system. If you do not yet have an ORCID you will be able to create one in minutes. If you are not listed as a corresponding author, please ensure that the corresponding author(s) comply.

1. From the home page of the MTS (http://mts-cddiscovery.nature.com/cgi-bin/main.plex) click on 'Modify my Springer Nature account' under 'General tasks'.
2. In the 'Personal profile' tab, click on 'ORCID Create/link an Open Researcher Contributor ID(ORCID)'. This will redirect you to the ORCID website.
3a. If you already have an ORCID account, enter your ORCID email and password and click on 'Authorize' to link your ORCID with your account on the MTS.

3b. If you don't yet have an ORCID, you can easily create one by providing the required information and then click on 'Authorize'. This will link your newly created ORCID with your account on the MTS.

Yours sincerely,

Editorial Office
Cell Death & Discovery
cddiscovery@springernature.com

This email has been sent through the Springer Nature Manuscript Tracking System NY-610A-NPG&MTS

Confidentiality Statement:

This e-mail is confidential and subject to copyright. Any unauthorised use or disclosure of its contents is prohibited. If you have received this email in error please notify our Manuscript Tracking System Helpdesk team at http://platformsupport.nature.com .

Details of the confidentiality and pre-publicity policy may be found here http://www.nature.com/authors/policies/confidentiality.html

Privacy Policy | Update Profile

Peng, Renwang

From: ZHANG YANG <fjxh_zhang2021@163.com>
Sent: Monday, December 26, 2022 2:40 PM
To: Peng, Renwang
Cc: Haitang YANG; Gao, Yanyun (DBMR) (yanyun.gao@dbmr.unibe.ch) (yanyun.gao@dbmr.unibe.ch); xuduo@jsph.org.cn; 许可; Liang, Shun-Qing; Hall, Sean; Amina Scherz; Forster, Stefan (DBMR); Berezowska Sabina; Feng Yao; Ochsenbein, Adrian; Marti, Thomas Michael; gregor.kocher@hin.ch; Ralph Alexander Schmid; Dorn, Patrick
Subject: Re:FW: CDDISCOVERY-22-5205R1 Initial Quality Check

I agree!

At 2022-12-23 01:08:00, "Peng, Renwang" <Renwang.Peng@insel.ch> wrote:

> Dear co-authors,
>
> Good news: **our manuscript has been provisionally accepted!**
>
> As indicated in the email below, Mr. Ke XU (Shanghai Chest Hospital), who performed the in vivo mice experiments for the revision, has been added as a new author.
>
> So one more step towards full acceptance is still to come:
>
> @All of you: please reply to me if you agree with this change.
>
> @Prof. Schmid and Patrick: please link your ORCID with your accounts on the MTS as described in the email.
>
> Thank you very much for the fruitful collaboration! I wish you have happy holidays and a prosperous 2023.
>
> Ren

From: cddiscovery@springernature.com <cddiscovery@springernature.com>
Sent: Thursday, December 22, 2022 5:01 PM
To: Peng, Renwang <Renwang.Peng@insel.ch>
Subject: CDDISCOVERY-22-5205R1 Initial Quality Check

Manuscript Number: CDDISCOVERY-22-5205R1

Title: MEK1 drives oncogenic signalling and interacts with PARP1 for genomic and metabolic homeostasis in malignant pleural mesothelioma

Authors: Ren-Wang Peng, Haitang Yang, Yanyun Gao, Duo Xu, Ke Xu, Shunqing Liang, Zhang Yang, Amina Scherz, Sean Hall, Stefan Forster, Sabina Berezowska, Feng Yao, Adrian Ochsenbein, Thomas Marti, Gregor Kocher, Ralph Schmid, Patrick Dorn

Dear Professor Peng,

After carrying out a quality check of your manuscript submitted to *Cell Death Discovery*, it has come to our attention that the following points need to be addressed before we are able to pass it onto the Editors for their consideration:

1. It has come to our attention that your most recent author list differs from the one in your original submission.

Please request agreement from all authors including additions and deletions, these can be collected in the following way:Email your co-authors with the change, and ask them to reply to your email confirming that they agree to these changes. Once you have collected these replies, please combine all of the co-authors' email responses in one document and upload this file to your submission.

2.Please make sure all corresponding authors link their own ORCID.
As part of our efforts to improve transparency in authorship, we now request that all authors identified as 'corresponding author' create and link their Open Researcher and Contributor Identifier (ORCID) with their account on the Manuscript Tracking System (MTS) prior to acceptance. For more information please visit http://www.springernature.com/orcid
Please follow the steps below to link your ORCID to your account on our MTS. Please ensure the ORCID is linked to your account associated with this manuscript, and not to any other account you may have on our system. If you do not yet have an ORCID you will be able to create one in minutes. If you are not listed as a corresponding author, please ensure that the corresponding author(s) comply.

1. From the home page of the MTS (http://mts-cddiscovery.nature.com/cgi-bin/main.plex) click on 'Modify my Springer Nature account' under 'General tasks'.
2. In the 'Personal profile' tab, click on 'ORCID Create/link an Open Researcher Contributor ID(ORCID)'. This will redirect you to the ORCID website.
3a. If you already have an ORCID account, enter your ORCID email and password and click on 'Authorize' to link your ORCID with your account on the MTS.

3b. If you don't yet have an ORCID, you can easily create one by providing the required information and then click on 'Authorize'. This will link your newly created ORCID with your account on the MTS.

Yours sincerely,

Editorial Office
Cell Death & Discovery

cddiscovery@springernature.com

This email has been sent through the Springer Nature Manuscript Tracking System NY-610A-NPG&MTS

Confidentiality Statement:

This e-mail is confidential and subject to copyright. Any unauthorised use or disclosure of its contents is prohibited. If you have received this email in error please notify our Manuscript Tracking System Helpdesk team at http://platformsupport.nature.com .

Details of the confidentiality and pre-publicity policy may be found here http://www.nature.com/authors/policies/confidentiality.html

Privacy Policy | Update Profile

Peng, Renwang

From: Scherz Amina <amina.scherz@spital.so.ch>
Sent: Friday, December 23, 2022 4:50 PM
To: Peng, Renwang
Subject: AW: [Externe Email] FW: CDDISCOVERY-22-5205R1 Initial Quality Check [HIN secured]

Dear Ren

Congratulations! Yes, agree with the change of authorship.
Wish you and your family happy holidays and a happy new year.
Best regards, Amina

Dr. med. Amina Scherz
Leitende Ärztin
Zentrum für Onkologie & Hämatologie

solothurner spitäler soH

Solothurner Spitäler AG | Bürgerspital Solothurn | Schöngrünstrasse 42 | 4500 Solothurn | T 032 627 4716 |
amina.scherz@spital.so.ch

Von: Peng, Renwang <Renwang.Peng@insel.ch>
Gesendet: Freitag, 23. Dezember 2022 16:15
An: Scherz Amina <amina.scherz@spital.so.ch>
Betreff: [Externe Email] FW: CDDISCOVERY-22-5205R1 Initial Quality Check [HIN secured]

ACHTUNG: Diese Email kommt von einem externen Sender ausserhalb der soH. Bitte klicken Sie nicht auf Links und öffnen Sie keine Anhänge, wenn der Sender unbekannt ist oder Sie nicht sicher sind, dass der Inhalt vertrauenswürdig ist.

Dear Amina,

Please reply to this email if you agree with the change of authorship.

Wish you all the best!

KR, Ren

From: Peng, Renwang
Sent: Thursday, December 22, 2022 6:08 PM
To: 'Haitang YANG' <yanghaitang@shchest.org>; Gao, Yanyun (DBMR) (yanyun.gao@dbmr.unibe.ch) (yanyun.gao@dbmr.unibe.ch) <yanyun.gao@dbmr.unibe.ch>; 'xuduo@jsph.org.cn' <xuduo@jsph.org.cn>; '许可' <xukeqaz1997@126.com>; 'Liang, Shun-Qing' <Shun-Qing.Liang@umassmed.edu>; 'fjxh_zhang2021@163.com' <fjxh_zhang2021@163.com>; 'Hall, Sean' <Sean.Hall@wyss.harvard.edu>; Amina Scherz <amina.scherz@icloud.com>; Forster, Stefan (DBMR) <stefan.forster@dbmr.unibe.ch>; 'Berezowska Sabina' <Sabina.Berezowska@chuv.ch>; Feng Yao <yaofeng6796678@126.com>; Ochsenbein, Adrian <Adrian.Ochsenbein@insel.ch>; Marti, Thomas Michael <Thomas.Marti@insel.ch>; 'gregor.kocher@hin.ch' <gregor.kocher@hin.ch>; Ralph Alexander Schmid <ralph_a_schmid@hotmail.com>; Dorn, Patrick <Patrick.Dorn@insel.ch>
Subject: FW: CDDISCOVERY-22-5205R1 Initial Quality Check

Dear co-authors,

Good news: **our manuscript has been provisionally accepted!**

As indicated in the email below, Mr. Ke XU (Shanghai Chest Hospital), who performed the in vivo mice experiments for the revision, has been added as a new author.

So one more step towards full acceptance is still to come:

@All of you: please reply to me if you agree with this change.

@Prof. Schmid and Patrick: please link your ORCID with your accounts on the MTS as described in the email.

Thank you very much for the fruitful collaboration! I wish you have happy holidays and a prosperous 2023.

Ren

From: cddiscovery@springernature.com <cddiscovery@springernature.com>
Sent: Thursday, December 22, 2022 5:01 PM
To: Peng, Renwang <Renwang.Peng@insel.ch>
Subject: CDDISCOVERY-22-5205R1 Initial Quality Check

Manuscript Number: CDDISCOVERY-22-5205R1

Title: MEK1 drives oncogenic signalling and interacts with PARP1 for genomic and metabolic homeostasis in malignant pleural mesothelioma

Authors: Ren-Wang Peng, Haitang Yang, Yanyun Gao, Duo Xu, Ke Xu, Shunqing Liang, Zhang Yang, Amina Scherz, Sean Hall, Stefan Forster, Sabina Berezowska, Feng Yao, Adrian Ochsenbein, Thomas Marti, Gregor Kocher, Ralph Schmid, Patrick Dorn

Dear Professor Peng,

After carrying out a quality check of your manuscript submitted to *Cell Death Discovery*, it has come to our attention that the following points need to be addressed before we are able to pass it onto the Editors for their consideration:

1. It has come to our attention that your most recent author list differs from the one in your original submission.

Please request agreement from all authors including additions and deletions, these can be collected in the following way:Email your co-authors with the change, and ask them to reply to your email confirming that they agree to these changes. Once you have collected these replies, please combine all of the co-authors' email responses in one document and upload this file to your submission.

2.Please make sure all corresponding authors link their own ORCID.
As part of our efforts to improve transparency in authorship, we now request that all authors identified as 'corresponding author' create and link their Open Researcher and Contributor Identifier (ORCID) with their account on the Manuscript Tracking System (MTS) prior to acceptance. For more information please visit

http://www.springernature.com/orcid
Please follow the steps below to link your ORCID to your account on our MTS. Please ensure the ORCID is linked to your account associated with this manuscript, and not to any other account you may have on our system. If you do not yet have an ORCID you will be able to create one in minutes. If you are not listed as a corresponding author, please ensure that the corresponding author(s) comply.

1. From the home page of the MTS (http://mts-cddiscovery.nature.com/cgi-bin/main.plex) click on ‘Modify my Springer Nature account’ under ‘General tasks’.
2. In the ‘Personal profile’ tab, click on ‘ORCID Create/link an Open Researcher Contributor ID(ORCID)’. This will redirect you to the ORCID website.
3a. If you already have an ORCID account, enter your ORCID email and password and click on ‘Authorize’ to link your ORCID with your account on the MTS.

3b. If you don’t yet have an ORCID, you can easily create one by providing the required information and then click on ‘Authorize’. This will link your newly created ORCID with your account on the MTS.

Yours sincerely,

Editorial Office
Cell Death & Discovery
cddiscovery@springernature.com

This email has been sent through the Springer Nature Manuscript Tracking System NY-610A-NPG&MTS

Confidentiality Statement:

This e-mail is confidential and subject to copyright. Any unauthorised use or disclosure of its contents is prohibited. If you have received this email in error please notify our Manuscript Tracking System Helpdesk team at http://platformsupport.nature.com .

Details of the confidentiality and pre-publicity policy may be found here http://www.nature.com/authors/policies/confidentiality.html

Privacy Policy | Update Profile

Peng, Renwang

From: Sean <srrhall@protonmail.com>
Sent: Friday, December 23, 2022 3:08 PM
To: Peng, Renwang
Subject: Re: FW: CDDISCOVERY-22-5205R1 Initial Quality Check

Congratulations Ren. I agree .

Kind regards
Sean

Sean Hall

Sent with Proton Mail secure email.

------- Original Message -------
On Friday, December 23rd, 2022 at 8:03 AM, Peng, Renwang <Renwang.Peng@insel.ch> wrote:

From: Peng, Renwang
Sent: Thursday, December 22, 2022 6:08 PM
To: 'Haitang YANG' <yanghaitang@shchest.org>; Gao, Yanyun (DBMR) (yanyun.gao@dbmr.unibe.ch) (yanyun.gao@dbmr.unibe.ch) <yanyun.gao@dbmr.unibe.ch>; 'xuduo@jsph.org.cn' <xuduo@jsph.org.cn>; '许可' <xukeqaz1997@126.com>; 'Liang, Shun-Qing' <Shun-Qing.Liang@umassmed.edu>; 'fjxh_zhang2021@163.com' <fjxh_zhang2021@163.com>; 'Hall, Sean' <Sean.Hall@wyss.harvard.edu>; Amina Scherz <amina.scherz@icloud.com>; Forster, Stefan (DBMR) <stefan.forster@dbmr.unibe.ch>; 'Berezowska Sabina' <Sabina.Berezowska@chuv.ch>; Feng Yao <yaofeng6796678@126.com>; Ochsenbein, Adrian <Adrian.Ochsenbein@insel.ch>; Marti, Thomas Michael <Thomas.Marti@insel.ch>; 'gregor.kocher@hin.ch' <gregor.kocher@hin.ch>; Ralph Alexander Schmid <ralph_a_schmid@hotmail.com>; Dorn, Patrick <Patrick.Dorn@insel.ch>
Subject: FW: CDDISCOVERY-22-5205R1 Initial Quality Check

Dear co-authors,

Good news: **our manuscript has been provisionally accepted!**

As indicated in the email below, Mr. Ke XU (Shanghai Chest Hospital), who performed the in vivo mice experiments for the revision, has been added as a new author.

So one more step towards full acceptance is still to come:

@All of you: please reply to me if you agree with this change.

@Prof. Schmid and Patrick: please link your ORCID with your accounts on the MTS as described in the email.

Thank you very much for the fruitful collaboration! I wish you have happy holidays and a prosperous 2023.

Ren

From: cddiscovery@springernature.com <cddiscovery@springernature.com>
Sent: Thursday, December 22, 2022 5:01 PM
To: Peng, Renwang <Renwang.Peng@insel.ch>
Subject: CDDISCOVERY-22-5205R1 Initial Quality Check

Manuscript Number: CDDISCOVERY-22-5205R1

Title: MEK1 drives oncogenic signalling and interacts with PARP1 for genomic and metabolic homeostasis in malignant pleural mesothelioma

Authors: Ren-Wang Peng, Haitang Yang, Yanyun Gao, Duo Xu, Ke Xu, Shunqing Liang, Zhang Yang, Amina Scherz, Sean Hall, Stefan Forster, Sabina Berezowska, Feng Yao, Adrian Ochsenbein, Thomas Marti, Gregor Kocher, Ralph Schmid, Patrick Dorn

Dear Professor Peng,

After carrying out a quality check of your manuscript submitted to *Cell Death Discovery*, it has come to our attention that the following points need to be addressed before we are able to pass it onto the Editors for their consideration:

1. It has come to our attention that your most recent author list differs from the one in your original submission.

Please request agreement from all authors including additions and deletions, these can be collected in the following way:Email your co-authors with the change, and ask them to reply to your email confirming that they agree to these changes. Once you have collected these replies, please combine all of the co-authors' email responses in one document and upload this file to your submission.

2.Please make sure all corresponding authors link their own ORCID.
As part of our efforts to improve transparency in authorship, we now request that all authors identified as 'corresponding author' create and link their Open Researcher and Contributor Identifier (ORCID) with their account on the Manuscript Tracking System (MTS) prior to acceptance. For more information please visit http://www.springernature.com/orcid
Please follow the steps below to link your ORCID to your account on our MTS. Please ensure the ORCID is linked to your account associated with this manuscript, and not to any other account you may have on our system. If you do not yet have an ORCID you will be able to create one in minutes. If you are not listed as a corresponding author, please ensure that the corresponding author(s) comply.

1. From the home page of the MTS (http://mts-cddiscovery.nature.com/cgi-bin/main.plex) click on 'Modify my Springer Nature account' under 'General tasks'.
2. In the 'Personal profile' tab, click on 'ORCID Create/link an Open Researcher Contributor ID(ORCID)'. This will redirect you to the ORCID website.
3a. If you already have an ORCID account, enter your ORCID email and password and click on 'Authorize' to link your ORCID with your account on the MTS.

3b. If you don't yet have an ORCID, you can easily create one by providing the required information and then click on 'Authorize'. This will link your newly created ORCID with your account on the MTS.

Yours sincerely,

Editorial Office
Cell Death & Discovery
cddiscovery@springernature.com

This email has been sent through the Springer Nature Manuscript Tracking System NY-610A-NPG&MTS

Confidentiality Statement:

This e-mail is confidential and subject to copyright. Any unauthorised use or disclosure of its contents is prohibited. If you have received this email in error please notify our Manuscript Tracking System Helpdesk team at http://platformsupport.nature.com .

Details of the confidentiality and pre-publicity policy may be found here http://www.nature.com/authors/policies/confidentiality.html

Privacy Policy | Update Profile

Peng, Renwang

From: Forster, Stefan (DBMR) <stefan.forster@unibe.ch>
Sent: Thursday, December 22, 2022 7:53 PM
To: Peng, Renwang
Subject: AW: CDDISCOVERY-22-5205R1 Initial Quality Check [HIN secured]

Dear Ren,

Congratulations to this great achievement.

I completely accept the according changes.

Thank you very much.

Best regards,
Stefan

Von: Peng, Renwang <Renwang.Peng@insel.ch>
Gesendet: Donnerstag, 22. Dezember 2022 18:08:00
An: Haitang YANG; Gao, Yanyun (DBMR); xuduo@jsph.org.cn; 许可; Liang, Shun-Qing; fjxh_zhang2021@163.com; Hall, Sean; Amina Scherz; Forster, Stefan (DBMR); Berezowska Sabina; Feng Yao; Ochsenbein, Adrian; Marti, Thomas Michael; gregor.kocher@hin.ch; Ralph Alexander Schmid; Dorn, Patrick
Betreff: FW: CDDISCOVERY-22-5205R1 Initial Quality Check

Dear co-authors,

Good news: **our manuscript has been provisionally accepted!**

As indicated in the email below, Mr. Ke XU (Shanghai Chest Hospital), who performed the in vivo mice experiments for the revision, has been added as a new author.

So one more step towards full acceptance is still to come:

@All of you: please reply to me if you agree with this change.

@Prof. Schmid and Patrick: please link your ORCID with your accounts on the MTS as described in the email.

Thank you very much for the fruitful collaboration! I wish you have happy holidays and a prosperous 2023.

Ren

From: cddiscovery@springernature.com <cddiscovery@springernature.com>
Sent: Thursday, December 22, 2022 5:01 PM
To: Peng, Renwang <Renwang.Peng@insel.ch>
Subject: CDDISCOVERY-22-5205R1 Initial Quality Check

Manuscript Number: CDDISCOVERY-22-5205R1

Title: MEK1 drives oncogenic signalling and interacts with PARP1 for genomic and metabolic homeostasis in malignant pleural mesothelioma

Authors: Ren-Wang Peng, Haitang Yang, Yanyun Gao, Duo Xu, Ke Xu, Shunqing Liang, Zhang Yang, Amina Scherz, Sean Hall, Stefan Forster, Sabina Berezowska, Feng Yao, Adrian Ochsenbein, Thomas Marti, Gregor Kocher, Ralph Schmid, Patrick Dorn

Dear Professor Peng,

After carrying out a quality check of your manuscript submitted to *Cell Death Discovery*, it has come to our attention that the following points need to be addressed before we are able to pass it onto the Editors for their consideration:

1. It has come to our attention that your most recent author list differs from the one in your original submission.

Please request agreement from all authors including additions and deletions, these can be collected in the following way:Email your co-authors with the change, and ask them to reply to your email confirming that they agree to these changes. Once you have collected these replies, please combine all of the co-authors' email responses in one document and upload this file to your submission.

2.Please make sure all corresponding authors link their own ORCID.
As part of our efforts to improve transparency in authorship, we now request that all authors identified as 'corresponding author' create and link their Open Researcher and Contributor Identifier (ORCID) with their account on the Manuscript Tracking System (MTS) prior to acceptance. For more information please visit http://www.springernature.com/orcid
Please follow the steps below to link your ORCID to your account on our MTS. Please ensure the ORCID is linked to your account associated with this manuscript, and not to any other account you may have on our system. If you do not yet have an ORCID you will be able to create one in minutes. If you are not listed as a corresponding author, please ensure that the corresponding author(s) comply.

1. From the home page of the MTS (http://mts-cddiscovery.nature.com/cgi-bin/main.plex) click on 'Modify my Springer Nature account' under 'General tasks'.
2. In the 'Personal profile' tab, click on 'ORCID Create/link an Open Researcher Contributor ID(ORCID)'. This will redirect you to the ORCID website.
3a. If you already have an ORCID account, enter your ORCID email and password and click on 'Authorize' to link your ORCID with your account on the MTS.

3b. If you don't yet have an ORCID, you can easily create one by providing the required information and then click on 'Authorize'. This will link your newly created ORCID with your account on the MTS.

Yours sincerely,

Editorial Office
Cell Death & Discovery
cddiscovery@springernature.com

This email has been sent through the Springer Nature Manuscript Tracking System NY-610A-NPG&MTS

Confidentiality Statement:

This e-mail is confidential and subject to copyright. Any unauthorised use or disclosure of its contents is prohibited. If you have received this email in error please notify our Manuscript Tracking System Helpdesk team at http://platformsupport.nature.com .

Details of the confidentiality and pre-publicity policy may be found here http://www.nature.com/authors/policies/confidentiality.html

Privacy Policy | Update Profile

Peng, Renwang

From: Berezowska Sabina <Sabina.Berezowska@chuv.ch>
Sent: Friday, December 23, 2022 9:44 AM
To: Peng, Renwang; Haitang YANG; Gao, Yanyun (DBMR) (yanyun.gao@dbmr.unibe.ch) (yanyun.gao@dbmr.unibe.ch); xuduo@jsph.org.cn; 许可; Liang, Shun-Qing; fjxh_zhang2021@163.com; Hall, Sean; Amina Scherz; Forster, Stefan (DBMR); Feng Yao; Ochsenbein, Adrian; Marti, Thomas Michael; gregor.kocher@hin.ch; Ralph Alexander Schmid; Dorn, Patrick
Subject: RE: CDDISCOVERY-22-5205R1 Initial Quality Check [HIN secured]

Dear Ren
Congratulation ! Impressive, and a really a nice Christmas present!
Of course, I agree with the authorship change.

Wishing you all a very merry holiday season and All the Best for 2013!
Sabina

De : Peng, Renwang <Renwang.Peng@insel.ch>
Envoyé : jeudi, 22 décembre 2022 18:08
À : Haitang YANG <yanghaitang@shchest.org>; Gao, Yanyun (DBMR) (yanyun.gao@dbmr.unibe.ch) (yanyun.gao@dbmr.unibe.ch) <yanyun.gao@dbmr.unibe.ch>; xuduo@jsph.org.cn; 许可 <xukeqaz1997@126.com>; Liang, Shun-Qing <Shun-Qing.Liang@umassmed.edu>; fjxh_zhang2021@163.com; Hall, Sean <Sean.Hall@wyss.harvard.edu>; Amina Scherz <amina.scherz@icloud.com>; Forster, Stefan (DBMR) <stefan.forster@dbmr.unibe.ch>; Berezowska Sabina <Sabina.Berezowska@chuv.ch>; Feng Yao <yaofeng6796678@126.com>; Ochsenbein, Adrian <Adrian.Ochsenbein@insel.ch>; Marti, Thomas Michael <Thomas.Marti@insel.ch>; gregor.kocher@hin.ch; Ralph Alexander Schmid <ralph_a_schmid@hotmail.com>; Dorn, Patrick <Patrick.Dorn@insel.ch>
Objet : FW: CDDISCOVERY-22-5205R1 Initial Quality Check [HIN secured]

Dear co-authors,

Good news: **our manuscript has been provisionally accepted!**

As indicated in the email below, Mr. Ke XU (Shanghai Chest Hospital), who performed the in vivo mice experiments for the revision, has been added as a new author.

So one more step towards full acceptance is still to come:

@All of you: please reply to me if you agree with this change.

@Prof. Schmid and Patrick: please link your ORCID with your accounts on the MTS as described in the email.

Thank you very much for the fruitful collaboration! I wish you have happy holidays and a prosperous 2023.

Ren

From: cddiscovery@springernature.com <cddiscovery@springernature.com>
Sent: Thursday, December 22, 2022 5:01 PM
To: Peng, Renwang <Renwang.Peng@insel.ch>
Subject: CDDISCOVERY-22-5205R1 Initial Quality Check

Manuscript Number: CDDISCOVERY-22-5205R1

Title: MEK1 drives oncogenic signalling and interacts with PARP1 for genomic and metabolic homeostasis in malignant pleural mesothelioma

Authors: Ren-Wang Peng, Haitang Yang, Yanyun Gao, Duo Xu, Ke Xu, Shunqing Liang, Zhang Yang, Amina Scherz, Sean Hall, Stefan Forster, Sabina Berezowska, Feng Yao, Adrian Ochsenbein, Thomas Marti, Gregor Kocher, Ralph Schmid, Patrick Dorn

Dear Professor Peng,

After carrying out a quality check of your manuscript submitted to *Cell Death Discovery*, it has come to our attention that the following points need to be addressed before we are able to pass it onto the Editors for their consideration:

1. It has come to our attention that your most recent author list differs from the one in your original submission.

Please request agreement from all authors including additions and deletions, these can be collected in the following way:Email your co-authors with the change, and ask them to reply to your email confirming that they agree to these changes. Once you have collected these replies, please combine all of the co-authors' email responses in one document and upload this file to your submission.

2.Please make sure all corresponding authors link their own ORCID.
As part of our efforts to improve transparency in authorship, we now request that all authors identified as 'corresponding author' create and link their Open Researcher and Contributor Identifier (ORCID) with their account on the Manuscript Tracking System (MTS) prior to acceptance. For more information please visit http://www.springernature.com/orcid
Please follow the steps below to link your ORCID to your account on our MTS. Please ensure the ORCID is linked to your account associated with this manuscript, and not to any other account you may have on our system. If you do not yet have an ORCID you will be able to create one in minutes. If you are not listed as a corresponding author, please ensure that the corresponding author(s) comply.

1. From the home page of the MTS (http://mts-cddiscovery.nature.com/cgi-bin/main.plex) click on 'Modify my Springer Nature account' under 'General tasks'.
2. In the 'Personal profile' tab, click on 'ORCID Create/link an Open Researcher Contributor ID(ORCID)'. This will redirect you to the ORCID website.
3a. If you already have an ORCID account, enter your ORCID email and password and click on 'Authorize' to link your ORCID with your account on the MTS.

3b. If you don't yet have an ORCID, you can easily create one by providing the required information and then click on 'Authorize'. This will link your newly created ORCID with your account on the MTS.

Yours sincerely,

Editorial Office
Cell Death & Discovery
cddiscovery@springernature.com

This email has been sent through the Springer Nature Manuscript Tracking System NY-610A-NPG&MTS

Confidentiality Statement:

This e-mail is confidential and subject to copyright. Any unauthorised use or disclosure of its contents is prohibited. If you have received this email in error please notify our Manuscript Tracking System Helpdesk team at http://platformsupport.nature.com .

Details of the confidentiality and pre-publicity policy may be found here http://www.nature.com/authors/policies/confidentiality.html

Privacy Policy | Update Profile

From: 姚烽 <728001275@shsmu.edu.cn>
Sent: Saturday, December 24, 2022 10:11 AM
To: Peng, Renwang
Cc: xukeqaz@sjtu.edu.cn
Subject: Re:FW: CDDISCOVERY-22-5205R1 Initial Quality Check

Dear Prof. Ren-Wang Peng:

I agree with this change！

Merry Christmas and happy new year！

Best regards,
Feng Yao

Prof. Dr. Feng Yao, MD-PhD

West Huaihai 241, Xuhui District

Department of Thoracic Surgery

Shanghai Chest Hospital, Shanghai Jiao Tong University
Shanghai, 200030, China

Shanghai Chest Hospital (shxkyy.com)

发件人："Peng, Renwang" <Renwang.Peng@insel.ch>
发送日期：2022-12-24 16:50:51
收件人："yaofeng@shsmu.edu.cn" <yaofeng@shsmu.edu.cn>,"xukeqaz@sjtu.edu.cn" <xukeqaz@sjtu.edu.cn>
主题：FW: CDDISCOVERY-22-5205R1 Initial Quality Check

From: Peng, Renwang
Sent: Thursday, December 22, 2022 6:08 PM
To: 'Haitang YANG' <yanghaitang@shchest.org>; Gao, Yanyun (DBMR) (yanyun.gao@dbmr.unibe.ch) (yanyun.gao@dbmr.unibe.ch) <yanyun.gao@dbmr.unibe.ch>; 'xuduo@jsph.org.cn' <xuduo@jsph.org.cn>; '许可' <xukeqaz1997@126.com>; 'Liang, Shun-Qing' <Shun-Qing.Liang@umassmed.edu>; 'fjxh_zhang2021@163.com' <fjxh_zhang2021@163.com>; 'Hall, Sean' <Sean.Hall@wyss.harvard.edu>; Amina Scherz <amina.scherz@icloud.com>; Forster, Stefan (DBMR) <stefan.forster@dbmr.unibe.ch>; 'Berezowska Sabina' <Sabina.Berezowska@chuv.ch>; Feng Yao <yaofeng6796678@126.com>; Ochsenbein, Adrian <Adrian.Ochsenbein@insel.ch>; Marti, Thomas Michael <Thomas.Marti@insel.ch>; 'gregor.kocher@hin.ch' <gregor.kocher@hin.ch>; Ralph Alexander Schmid <ralph_a_schmid@hotmail.com>; Dorn, Patrick <Patrick.Dorn@insel.ch>
Subject: FW: CDDISCOVERY-22-5205R1 Initial Quality Check

Dear co-authors,

Good news: **our manuscript has been provisionally accepted!**

As indicated in the email below, Mr. Ke XU (Shanghai Chest Hospital), who performed the in vivo mice experiments for the revision, has been added as a new author.

So one more step towards full acceptance is still to come:

@All of you: please reply to me if you agree with this change.

@Prof. Schmid and Patrick: please link your ORCID with your accounts on the MTS as described in the email.

Thank you very much for the fruitful collaboration! I wish you have happy holidays and a prosperous 2023.

Ren

From: cddiscovery@springernature.com <cddiscovery@springernature.com>
Sent: Thursday, December 22, 2022 5:01 PM
To: Peng, Renwang <Renwang.Peng@insel.ch>
Subject: CDDISCOVERY-22-5205R1 Initial Quality Check

Manuscript Number: CDDISCOVERY-22-5205R1

Title: MEK1 drives oncogenic signalling and interacts with PARP1 for genomic and metabolic homeostasis in malignant pleural mesothelioma

Authors: Ren-Wang Peng, Haitang Yang, Yanyun Gao, Duo Xu, Ke Xu, Shunqing Liang, Zhang Yang, Amina Scherz, Sean Hall, Stefan Forster, Sabina Berezowska, Feng Yao, Adrian Ochsenbein, Thomas Marti, Gregor Kocher, Ralph Schmid, Patrick Dorn

Dear Professor Peng,

After carrying out a quality check of your manuscript submitted to *Cell Death Discovery*, it has come to our attention that the following points need to be addressed before we are able to pass it onto the Editors for their consideration:

1. It has come to our attention that your most recent author list differs from the one in your original submission.

Please request agreement from all authors including additions and deletions, these can be collected in the following way:Email your co-authors with the change, and ask them to reply to your email confirming that they agree to these changes. Once you have collected these replies, please combine all of the co-authors' email responses in one document and upload this file to your submission.

2.Please make sure all corresponding authors link their own ORCID.
As part of our efforts to improve transparency in authorship, we now request that all authors identified as 'corresponding author' create and link their Open Researcher and Contributor Identifier (ORCID) with their account on the Manuscript Tracking System (MTS) prior to acceptance. For more information please visit http://www.springernature.com/orcid
Please follow the steps below to link your ORCID to your account on our MTS. Please ensure the ORCID is linked to your account associated with this manuscript, and not to any other account you may have on our system. If you do not yet have an ORCID you will be able to create one in minutes. If you are not listed as a corresponding author, please ensure that the corresponding author(s) comply.

1. From the home page of the MTS (http://mts-cddiscovery.nature.com/cgi-bin/main.plex) click on 'Modify my Springer Nature account' under 'General tasks'.
2. In the 'Personal profile' tab, click on 'ORCID Create/link an Open Researcher Contributor ID(ORCID)'. This will redirect you to the ORCID website.
3a. If you already have an ORCID account, enter your ORCID email and password and click on 'Authorize' to link your ORCID with your account on the MTS.

3b. If you don't yet have an ORCID, you can easily create one by providing the required information and then click on 'Authorize'. This will link your newly created ORCID with your account on the MTS.

Yours sincerely,

Editorial Office
Cell Death & Discovery
cddiscovery@springernature.com

This email has been sent through the Springer Nature Manuscript Tracking System NY-610A-NPG&MTS

Confidentiality Statement:

This e-mail is confidential and subject to copyright. Any unauthorised use or disclosure of its contents is prohibited. If you have received this email in error please notify our Manuscript Tracking System Helpdesk team at http://platformsupport.nature.com .

Details of the confidentiality and pre-publicity policy may be found here http://www.nature.com/authors/policies/confidentiality.html

Privacy Policy | Update Profile

Peng, Renwang

From: Ochsenbein, Adrian
Sent: Friday, December 23, 2022 3:14 PM
To: Peng, Renwang
Subject: AW: CDDISCOVERY-22-5205R1 Initial Quality Check

Dear Ren,

congratulations! And thanks a lot for including me as a co-author.
I agree with the changes.

Merry Christmas and a Happy New Year.

Adrian

Adrian Ochsenbein, *MD, EMBA*
Prof. Dr. med.
Klinikdirektor und Chefarzt
INSELSPITAL, Universitätsspital Bern
Universitätsklinik für Medizinische Onkologie
Freiburgstrasse 10
3010 Bern
Telefon: +41 (0)31 632 84 42
Fax: +41 (0)31 632 41 19
E-Mail: adrian.ochsenbein@insel.ch
www.ochsenbeinlab.ch
www.insel.ch

Von: Peng, Renwang <Renwang.Peng@insel.ch>
Gesendet: Donnerstag, 22. Dezember 2022 18:08
An: Haitang YANG <yanghaitang@shchest.org>; Gao, Yanyun (DBMR) (yanyun.gao@dbmr.unibe.ch) (yanyun.gao@dbmr.unibe.ch) <yanyun.gao@dbmr.unibe.ch>; xuduo@jsph.org.cn; 许可 <xukeqaz1997@126.com>; Liang, Shun-Qing <Shun-Qing.Liang@umassmed.edu>; fjxh_zhang2021@163.com; Hall, Sean <Sean.Hall@wyss.harvard.edu>; Amina Scherz <amina.scherz@icloud.com>; Forster, Stefan (DBMR) <stefan.forster@dbmr.unibe.ch>; Berezowska Sabina <Sabina.Berezowska@chuv.ch>; Feng Yao <yaofeng6796678@126.com>; Ochsenbein, Adrian <Adrian.Ochsenbein@insel.ch>; Marti, Thomas Michael <Thomas.Marti@insel.ch>; gregor.kocher@hin.ch; Ralph Alexander Schmid <ralph_a_schmid@hotmail.com>; Dorn, Patrick <Patrick.Dorn@insel.ch>
Betreff: FW: CDDISCOVERY-22-5205R1 Initial Quality Check

Dear co-authors,

Good news: **our manuscript has been provisionally accepted!**

As indicated in the email below, Mr. Ke XU (Shanghai Chest Hospital), who performed the in vivo mice experiments for the revision, has been added as a new author.

So one more step towards full acceptance is still to come:

@All of you: please reply to me if you agree with this change.

@Prof. Schmid and Patrick: please link your ORCID with your accounts on the MTS as described in the email.

Thank you very much for the fruitful collaboration! I wish you have happy holidays and a prosperous 2023.

Ren

From: cddiscovery@springernature.com <cddiscovery@springernature.com>
Sent: Thursday, December 22, 2022 5:01 PM
To: Peng, Renwang <Renwang.Peng@insel.ch>
Subject: CDDISCOVERY-22-5205R1 Initial Quality Check

Manuscript Number: CDDISCOVERY-22-5205R1

Title: MEK1 drives oncogenic signalling and interacts with PARP1 for genomic and metabolic homeostasis in malignant pleural mesothelioma

Authors: Ren-Wang Peng, Haitang Yang, Yanyun Gao, Duo Xu, Ke Xu, Shunqing Liang, Zhang Yang, Amina Scherz, Sean Hall, Stefan Forster, Sabina Berezowska, Feng Yao, Adrian Ochsenbein, Thomas Marti, Gregor Kocher, Ralph Schmid, Patrick Dorn

Dear Professor Peng,

After carrying out a quality check of your manuscript submitted to *Cell Death Discovery*, it has come to our attention that the following points need to be addressed before we are able to pass it onto the Editors for their consideration:

1. It has come to our attention that your most recent author list differs from the one in your original submission.

Please request agreement from all authors including additions and deletions, these can be collected in the following way:Email your co-authors with the change, and ask them to reply to your email confirming that they agree to these changes. Once you have collected these replies, please combine all of the co-authors' email responses in one document and upload this file to your submission.

2.Please make sure all corresponding authors link their own ORCID.
As part of our efforts to improve transparency in authorship, we now request that all authors identified as 'corresponding author' create and link their Open Researcher and Contributor Identifier (ORCID) with their account on the Manuscript Tracking System (MTS) prior to acceptance. For more information please visit http://www.springernature.com/orcid
Please follow the steps below to link your ORCID to your account on our MTS. Please ensure the ORCID is linked to your account associated with this manuscript, and not to any other account you may have on our system. If you do not yet have an ORCID you will be able to create one in minutes. If you are not listed as a corresponding author, please ensure that the corresponding author(s) comply.

1. From the home page of the MTS (http://mts-cddiscovery.nature.com/cgi-bin/main.plex) click on 'Modify my Springer Nature account' under 'General tasks'.
2. In the 'Personal profile' tab, click on 'ORCID Create/link an Open Researcher Contributor ID(ORCID)'. This will redirect you to the ORCID website.
3a. If you already have an ORCID account, enter your ORCID email and password and click on 'Authorize' to link your ORCID with your account on the MTS.

3b. If you don't yet have an ORCID, you can easily create one by providing the required information and

then click on ‘Authorize’. This will link your newly created ORCID with your account on the MTS.

Yours sincerely,

Editorial Office
Cell Death & Discovery
cddiscovery@springernature.com

This email has been sent through the Springer Nature Manuscript Tracking System NY-610A-NPG&MTS

Confidentiality Statement:

This e-mail is confidential and subject to copyright. Any unauthorised use or disclosure of its contents is prohibited. If you have received this email in error please notify our Manuscript Tracking System Helpdesk team at http://platformsupport.nature.com .

Details of the confidentiality and pre-publicity policy may be found here http://www.nature.com/authors/policies/confidentiality.html

Privacy Policy | Update Profile

Peng, Renwang

**From:**	Marti, Thomas Michael
**Sent:**	Friday, December 23, 2022 9:11 AM
**To:**	Gao, Yanyun (DBMR); Peng, Renwang; Haitang YANG; xuduo@jsph.org.cn; 许可; Liang, Shun-Qing; fjxh_zhang2021@163.com; Hall, Sean; Amina Scherz; Forster, Stefan (DBMR); Berezowska Sabina; Feng Yao; Ochsenbein, Adrian; gregor.kocher@hin.ch; Ralph Alexander Schmid; Dorn, Patrick
**Subject:**	RE: CDDISCOVERY-22-5205R1 Initial Quality Check [HIN secured]

Hi Ren,

The perfect Christmas gift! ☺

I agree with the authorship change.

Merry Xmas, happy NY!
Tom

From: Gao, Yanyun (DBMR) <yanyun.gao@unibe.ch>
Sent: Freitag, 23. Dezember 2022 08:54
To: Peng, Renwang <Renwang.Peng@insel.ch>; Haitang YANG <yanghaitang@shchest.org>; xuduo@jsph.org.cn; 许可 <xukeqaz1997@126.com>; Liang, Shun-Qing <Shun-Qing.Liang@umassmed.edu>; fjxh_zhang2021@163.com; Hall, Sean <Sean.Hall@wyss.harvard.edu>; Amina Scherz <amina.scherz@icloud.com>; Forster, Stefan (DBMR) <stefan.forster@unibe.ch>; Berezowska Sabina <Sabina.Berezowska@chuv.ch>; Feng Yao <yaofeng6796678@126.com>; Ochsenbein, Adrian <Adrian.Ochsenbein@insel.ch>; Marti, Thomas Michael <Thomas.Marti@insel.ch>; gregor.kocher@hin.ch; Ralph Alexander Schmid <ralph_a_schmid@hotmail.com>; Dorn, Patrick <Patrick.Dorn@insel.ch>
Subject: RE: CDDISCOVERY-22-5205R1 Initial Quality Check [HIN secured]

Hi Ren,

I agree with the authorship change.

Best regards,
Yanyun

From: Peng, Renwang <Renwang.Peng@insel.ch>
Sent: Thursday, December 22, 2022 6:08 PM
To: Haitang YANG <yanghaitang@shchest.org>; Gao, Yanyun (DBMR) <yanyun.gao@unibe.ch>; xuduo@jsph.org.cn; 许可 <xukeqaz1997@126.com>; Liang, Shun-Qing <Shun-Qing.Liang@umassmed.edu>; fjxh_zhang2021@163.com; Hall, Sean <Sean.Hall@wyss.harvard.edu>; Amina Scherz <amina.scherz@icloud.com>; Forster, Stefan (DBMR) <stefan.forster@unibe.ch>; Berezowska Sabina <Sabina.Berezowska@chuv.ch>; Feng Yao <yaofeng6796678@126.com>; Ochsenbein, Adrian <Adrian.Ochsenbein@insel.ch>; Marti, Thomas Michael <Thomas.Marti@insel.ch>; gregor.kocher@hin.ch; Ralph Alexander Schmid <ralph_a_schmid@hotmail.com>; Dorn, Patrick <Patrick.Dorn@insel.ch>
Subject: FW: CDDISCOVERY-22-5205R1 Initial Quality Check

Dear co-authors,

Good news: **our manuscript has been provisionally accepted!**

As indicated in the email below, Mr. Ke XU (Shanghai Chest Hospital), who performed the in vivo mice experiments for the revision, has been added as a new author.

So one more step towards full acceptance is still to come:

@All of you: please reply to me if you agree with this change.

@Prof. Schmid and Patrick: please link your ORCID with your accounts on the MTS as described in the email.

Thank you very much for the fruitful collaboration! I wish you have happy holidays and a prosperous 2023.

Ren

From: cddiscovery@springernature.com <cddiscovery@springernature.com>
Sent: Thursday, December 22, 2022 5:01 PM
To: Peng, Renwang <Renwang.Peng@insel.ch>
Subject: CDDISCOVERY-22-5205R1 Initial Quality Check

Manuscript Number: CDDISCOVERY-22-5205R1

Title: MEK1 drives oncogenic signalling and interacts with PARP1 for genomic and metabolic homeostasis in malignant pleural mesothelioma

Authors: Ren-Wang Peng, Haitang Yang, Yanyun Gao, Duo Xu, Ke Xu, Shunqing Liang, Zhang Yang, Amina Scherz, Sean Hall, Stefan Forster, Sabina Berezowska, Feng Yao, Adrian Ochsenbein, Thomas Marti, Gregor Kocher, Ralph Schmid, Patrick Dorn

Dear Professor Peng,

After carrying out a quality check of your manuscript submitted to *Cell Death Discovery*, it has come to our attention that the following points need to be addressed before we are able to pass it onto the Editors for their consideration:

1. It has come to our attention that your most recent author list differs from the one in your original submission.

Please request agreement from all authors including additions and deletions, these can be collected in the following way:Email your co-authors with the change, and ask them to reply to your email confirming that they agree to these changes. Once you have collected these replies, please combine all of the co-authors' email responses in one document and upload this file to your submission.

2.Please make sure all corresponding authors link their own ORCID.
As part of our efforts to improve transparency in authorship, we now request that all authors identified as 'corresponding author' create and link their Open Researcher and Contributor Identifier (ORCID) with their account on the Manuscript Tracking System (MTS) prior to acceptance. For more information please visit http://www.springernature.com/orcid

Please follow the steps below to link your ORCID to your account on our MTS. Please ensure the ORCID is linked to your account associated with this manuscript, and not to any other account you may have on our system. If you do not yet have an ORCID you will be able to create one in minutes. If you are not listed as a corresponding author, please ensure that the corresponding author(s) comply.

1. From the home page of the MTS (http://mts-cddiscovery.nature.com/cgi-bin/main.plex) click on 'Modify my Springer Nature account' under 'General tasks'.
2. In the 'Personal profile' tab, click on 'ORCID Create/link an Open Researcher Contributor ID(ORCID)'. This will redirect you to the ORCID website.
3a. If you already have an ORCID account, enter your ORCID email and password and click on 'Authorize' to link your ORCID with your account on the MTS.

3b. If you don't yet have an ORCID, you can easily create one by providing the required information and then click on 'Authorize'. This will link your newly created ORCID with your account on the MTS.

Yours sincerely,

Editorial Office
Cell Death & Discovery
cddiscovery@springernature.com

This email has been sent through the Springer Nature Manuscript Tracking System NY-610A-NPG&MTS

Confidentiality Statement:

This e-mail is confidential and subject to copyright. Any unauthorised use or disclosure of its contents is prohibited. If you have received this email in error please notify our Manuscript Tracking System Helpdesk team at http://platformsupport.nature.com .

Details of the confidentiality and pre-publicity policy may be found here http://www.nature.com/authors/policies/confidentiality.html

Privacy Policy | Update Profile

Peng, Renwang

From: Gregor Kocher <gregor.kocher@hin.ch>
Sent: Thursday, December 22, 2022 6:42 PM
To: Peng, Renwang
Subject: Re: CDDISCOVERY-22-5205R1 Initial Quality Check [HIN secured]

Dear Ren
Of course ok - thanks for including me.
I hope that you, your research team and your family is well and that your research can thrive unhindered!

greetings also to the team!
br
Greg

On 22 Dec 2022, at 18:08, Peng, Renwang <Renwang.Peng@insel.ch> wrote:

Dear co-authors,

Good news: **our manuscript has been provisionally accepted!**

As indicated in the email below, Mr. Ke XU (Shanghai Chest Hospital), who performed the in vivo mice experiments for the revision, has been added as a new author.

So one more step towards full acceptance is still to come:

@All of you: please reply to me if you agree with this change.

@Prof. Schmid and Patrick: please link your ORCID with your accounts on the MTS as described in the email.

Thank you very much for the fruitful collaboration! I wish you have happy holidays and a prosperous 2023.

Ren

From: cddiscovery@springernature.com <cddiscovery@springernature.com>
Sent: Thursday, December 22, 2022 5:01 PM
To: Peng, Renwang <Renwang.Peng@insel.ch>
Subject: CDDISCOVERY-22-5205R1 Initial Quality Check

Manuscript Number: CDDISCOVERY-22-5205R1

Title: MEK1 drives oncogenic signalling and interacts with PARP1 for genomic and metabolic homeostasis in malignant pleural mesothelioma

Authors: Ren-Wang Peng, Haitang Yang, Yanyun Gao, Duo Xu, Ke Xu, Shunqing Liang, Zhang Yang, Amina Scherz, Sean Hall, Stefan Forster, Sabina Berezowska, Feng Yao, Adrian Ochsenbein, Thomas Marti, Gregor Kocher, Ralph Schmid, Patrick Dorn

Dear Professor Peng,

After carrying out a quality check of your manuscript submitted to *Cell Death Discovery*, it has come to our attention that the following points need to be addressed before we are able to pass it onto the Editors for their consideration:

1. It has come to our attention that your most recent author list differs from the one in your original submission.

Please request agreement from all authors including additions and deletions, these can be collected in the following way:Email your co-authors with the change, and ask them to reply to your email confirming that they agree to these changes. Once you have collected these replies, please combine all of the co-authors' email responses in one document and upload this file to your submission.

2.Please make sure all corresponding authors link their own ORCID.
As part of our efforts to improve transparency in authorship, we now request that all authors identified as 'corresponding author' create and link their Open Researcher and Contributor Identifier (ORCID) with their account on the Manuscript Tracking System (MTS) prior to acceptance. For more information please visit http://www.springernature.com/orcid
Please follow the steps below to link your ORCID to your account on our MTS. Please ensure the ORCID is linked to your account associated with this manuscript, and not to any other account you may have on our system. If you do not yet have an ORCID you will be able to create one in minutes. If you are not listed as a corresponding author, please ensure that the corresponding author(s) comply.

1. From the home page of the MTS (http://mts-cddiscovery.nature.com/cgi-bin/main.plex) click on 'Modify my Springer Nature account' under 'General tasks'.
2. In the 'Personal profile' tab, click on 'ORCID Create/link an Open Researcher Contributor ID(ORCID)'. This will redirect you to the ORCID website.
3a. If you already have an ORCID account, enter your ORCID email and password and click on 'Authorize' to link your ORCID with your account on the MTS.

3b. If you don't yet have an ORCID, you can easily create one by providing the required information and then click on 'Authorize'. This will link your newly created ORCID with your account on the MTS.

Yours sincerely,

Editorial Office
Cell Death & Discovery
cddiscovery@springernature.com

This email has been sent through the Springer Nature Manuscript Tracking System NY-610A-NPG&MTS

Confidentiality Statement:

This e-mail is confidential and subject to copyright. Any unauthorised use or disclosure of its contents is prohibited. If you have received this email in error please notify our Manuscript Tracking System Helpdesk team at http://platformsupport.nature.com .

Details of the confidentiality and pre-publicity policy may be found here http://www.nature.com/authors/policies/confidentiality.html

Privacy Policy | Update Profile

Peng, Renwang

**From:**	Ralph Schmid <ralph_a_schmid@hotmail.com>
**Sent:**	Saturday, December 24, 2022 11:43 AM
**To:**	Peng, Renwang
**Subject:**	Re: CDDISCOVERY-22-5205R1 Initial Quality Check

Fine with me !

Von meinem iPhone gesendet

Am 24.12.2022 um 09:46 schrieb Peng, Renwang <Renwang.Peng@insel.ch>:

Dear Prof. Schmid,

Merry Christmas!

One more thing, please reply to my original email that you agree with the authorship change: adding Ke Xu as a new author.

Best regards

Ren

From: Ralph Schmid <ralph_a_schmid@hotmail.com>
Sent: Thursday, December 22, 2022 8:50 PM
To: Peng, Renwang <Renwang.Peng@insel.ch>
Cc: Haitang YANG <yanghaitang@shchest.org>; Gao, Yanyun (DBMR) (yanyun.gao@dbmr.unibe.ch) (yanyun.gao@dbmr.unibe.ch) <yanyun.gao@dbmr.unibe.ch>; xuduo@jsph.org.cn; 许可 <xukeqaz1997@126.com>; Liang, Shun-Qing <Shun-Qing.Liang@umassmed.edu>; fjxh_zhang2021@163.com; Hall, Sean <Sean.Hall@wyss.harvard.edu>; Amina Scherz <amina.scherz@icloud.com>; Forster, Stefan (DBMR) <stefan.forster@dbmr.unibe.ch>; Berezowska Sabina <Sabina.Berezowska@chuv.ch>; Feng Yao <yaofeng6796678@126.com>; Ochsenbein, Adrian <Adrian.Ochsenbein@insel.ch>; Marti, Thomas Michael <Thomas.Marti@insel.ch>; gregor.kocher@hin.ch; Dorn, Patrick <Patrick.Dorn@insel.ch>
Subject: Re: CDDISCOVERY-22-5205R1 Initial Quality Check

Great congratulations ! Great achievement !!!
Happy new year
Ralph Schmid

Von meinem iPhone gesendet

Am 22.12.2022 um 18:08 schrieb Peng, Renwang <Renwang.Peng@insel.ch>:

Dear co-authors,

Good news: **our manuscript has been provisionally accepted!**

As indicated in the email below, Mr. Ke XU (Shanghai Chest Hospital), who performed the in vivo mice experiments for the revision, has been added as a new author.

So one more step towards full acceptance is still to come:

@All of you: please reply to me if you agree with this change.

@Prof. Schmid and Patrick: please link your ORCID with your accounts on the MTS as described in the email.

Thank you very much for the fruitful collaboration! I wish you have happy holidays and a prosperous 2023.

Ren

From: cddiscovery@springernature.com <cddiscovery@springernature.com>
Sent: Thursday, December 22, 2022 5:01 PM
To: Peng, Renwang <Renwang.Peng@insel.ch>
Subject: CDDISCOVERY-22-5205R1 Initial Quality Check

Manuscript Number: CDDISCOVERY-22-5205R1

Title: MEK1 drives oncogenic signalling and interacts with PARP1 for genomic and metabolic homeostasis in malignant pleural mesothelioma

Authors: Ren-Wang Peng, Haitang Yang, Yanyun Gao, Duo Xu, Ke Xu, Shunqing Liang, Zhang Yang, Amina Scherz, Sean Hall, Stefan Forster, Sabina Berezowska, Feng Yao, Adrian Ochsenbein, Thomas Marti, Gregor Kocher, Ralph Schmid, Patrick Dorn

Dear Professor Peng,

After carrying out a quality check of your manuscript submitted to *Cell Death Discovery*, it has come to our attention that the following points need to be addressed before we are able to pass it onto the Editors for their consideration:

1. It has come to our attention that your most recent author list differs from the one in your original submission.

Please request agreement from all authors including additions and deletions, these can be collected in the following way:Email your co-authors with the change, and ask them to reply to your email confirming that they agree to these changes. Once you have collected these replies, please combine all of the co-authors' email responses in one document and upload this file to your submission.

2.Please make sure all corresponding authors link their own ORCID.
As part of our efforts to improve transparency in authorship, we now request that all authors identified as 'corresponding author' create and link their Open Researcher and Contributor Identifier (ORCID) with their account on the Manuscript Tracking System (MTS) prior to acceptance. For more information please visit http://www.springernature.com/orcid
Please follow the steps below to link your ORCID to your account on our MTS. Please ensure the ORCID is linked to your account associated with this manuscript, and not to any other account you may have on our system. If you do not yet have an ORCID you will be able to create one in minutes. If you are not listed as a corresponding author, please ensure that the corresponding author(s) comply.

1. From the home page of the MTS (http://mts-cddiscovery.nature.com/cgi-bin/main.plex) click on 'Modify my Springer Nature account' under 'General tasks'.
2. In the 'Personal profile' tab, click on 'ORCID Create/link an Open Researcher Contributor ID(ORCID)'. This will redirect you to the ORCID website.
3a. If you already have an ORCID account, enter your ORCID email and password and click on 'Authorize' to link your ORCID with your account on the MTS.

3b. If you don't yet have an ORCID, you can easily create one by providing the required information and then click on 'Authorize'. This will link your newly created ORCID with your account on the MTS.

Yours sincerely,

Editorial Office
Cell Death & Discovery
cddiscovery@springernature.com

This email has been sent through the Springer Nature Manuscript Tracking System NY-610A-NPG&MTS

Confidentiality Statement:

This e-mail is confidential and subject to copyright. Any unauthorised use or disclosure of its contents is prohibited. If you have received this email in error please notify our Manuscript Tracking System Helpdesk team at http://platformsupport.nature.com .

Details of the confidentiality and pre-publicity policy may be found here http://www.nature.com/authors/policies/confidentiality.html

Privacy Policy | Update Profile

Peng, Renwang

From: Dorn, Patrick
Sent: Thursday, December 22, 2022 11:19 PM
To: Peng, Renwang
Subject: AW: CDDISCOVERY-22-5205R1 Initial Quality Check

Hi Ren

Congratulations! I agree By the way, I have already made the link with the ORCID last time. Did you get an email that it is still not done? It should actually work.

Thank you

Patrick

Von: Peng, Renwang <Renwang.Peng@insel.ch>
Gesendet: Donnerstag, 22. Dezember 2022 18:08
An: Haitang YANG <yanghaitang@shchest.org>; Gao, Yanyun (DBMR) (yanyun.gao@dbmr.unibe.ch) (yanyun.gao@dbmr.unibe.ch) <yanyun.gao@dbmr.unibe.ch>; xuduo@jsph.org.cn; 许可 <xukeqaz1997@126.com>; Liang, Shun-Qing <Shun-Qing.Liang@umassmed.edu>; fjxh_zhang2021@163.com; Hall, Sean <Sean.Hall@wyss.harvard.edu>; Amina Scherz <amina.scherz@icloud.com>; Forster, Stefan (DBMR) <stefan.forster@dbmr.unibe.ch>; Berezowska Sabina <Sabina.Berezowska@chuv.ch>; Feng Yao <yaofeng6796678@126.com>; Ochsenbein, Adrian <Adrian.Ochsenbein@insel.ch>; Marti, Thomas Michael <Thomas.Marti@insel.ch>; gregor.kocher@hin.ch; Ralph Alexander Schmid <ralph_a_schmid@hotmail.com>; Dorn, Patrick <Patrick.Dorn@insel.ch>
Betreff: FW: CDDISCOVERY-22-5205R1 Initial Quality Check

Dear co-authors,

Good news: **our manuscript has been provisionally accepted!**

As indicated in the email below, Mr. Ke XU (Shanghai Chest Hospital), who performed the in vivo mice experiments for the revision, has been added as a new author.

So one more step towards full acceptance is still to come:

@All of you: please reply to me if you agree with this change.

@Prof. Schmid and Patrick: please link your ORCID with your accounts on the MTS as described in the email.

Thank you very much for the fruitful collaboration! I wish you have happy holidays and a prosperous 2023.

Ren

From: cddiscovery@springernature.com <cddiscovery@springernature.com>
Sent: Thursday, December 22, 2022 5:01 PM
To: Peng, Renwang <Renwang.Peng@insel.ch>
Subject: CDDISCOVERY-22-5205R1 Initial Quality Check

Manuscript Number: CDDISCOVERY-22-5205R1

Title: MEK1 drives oncogenic signalling and interacts with PARP1 for genomic and metabolic homeostasis in malignant pleural mesothelioma

Authors: Ren-Wang Peng, Haitang Yang, Yanyun Gao, Duo Xu, Ke Xu, Shunqing Liang, Zhang Yang, Amina Scherz, Sean Hall, Stefan Forster, Sabina Berezowska, Feng Yao, Adrian Ochsenbein, Thomas Marti, Gregor Kocher, Ralph Schmid, Patrick Dorn

Dear Professor Peng,

After carrying out a quality check of your manuscript submitted to *Cell Death Discovery*, it has come to our attention that the following points need to be addressed before we are able to pass it onto the Editors for their consideration:

1. It has come to our attention that your most recent author list differs from the one in your original submission.

Please request agreement from all authors including additions and deletions, these can be collected in the following way:Email your co-authors with the change, and ask them to reply to your email confirming that they agree to these changes. Once you have collected these replies, please combine all of the co-authors' email responses in one document and upload this file to your submission.

2.Please make sure all corresponding authors link their own ORCID.
As part of our efforts to improve transparency in authorship, we now request that all authors identified as 'corresponding author' create and link their Open Researcher and Contributor Identifier (ORCID) with their account on the Manuscript Tracking System (MTS) prior to acceptance. For more information please visit http://www.springernature.com/orcid
Please follow the steps below to link your ORCID to your account on our MTS. Please ensure the ORCID is linked to your account associated with this manuscript, and not to any other account you may have on our system. If you do not yet have an ORCID you will be able to create one in minutes. If you are not listed as a corresponding author, please ensure that the corresponding author(s) comply.

1. From the home page of the MTS (http://mts-cddiscovery.nature.com/cgi-bin/main.plex) click on 'Modify my Springer Nature account' under 'General tasks'.
2. In the 'Personal profile' tab, click on 'ORCID Create/link an Open Researcher Contributor ID(ORCID)'. This will redirect you to the ORCID website.
3a. If you already have an ORCID account, enter your ORCID email and password and click on 'Authorize' to link your ORCID with your account on the MTS.

3b. If you don't yet have an ORCID, you can easily create one by providing the required information and then click on 'Authorize'. This will link your newly created ORCID with your account on the MTS.

Yours sincerely,

Editorial Office
Cell Death & Discovery
cddiscovery@springernature.com

This email has been sent through the Springer Nature Manuscript Tracking System NY-610A-NPG&MTS

Confidentiality Statement:

This e-mail is confidential and subject to copyright. Any unauthorised use or disclosure of its contents is prohibited. If you have received this email in error please notify our Manuscript Tracking System Helpdesk team at http://platformsupport.nature.com .

Details of the confidentiality and pre-publicity policy may be found here http://www.nature.com/authors/policies/confidentiality.html

Privacy Policy | Update Profile
